# *Pseudomonas aeruginosa* and *Staphylococcus aureus* Display Differential Proteomic Responses to the Silver(I) Compound, SBC3

**DOI:** 10.3390/antibiotics12020348

**Published:** 2023-02-08

**Authors:** Magdalena Piatek, Cillian O’Beirne, Zoe Beato, Matthias Tacke, Kevin Kavanagh

**Affiliations:** 1 SSPC Pharma Research Centre, Department of Biology, Maynooth University, W23 F2K8 Maynooth, Co. Kildare, Ireland; 2 School of Chemistry, University College Dublin, D04 V1W8 Belfield, Dublin 4, Ireland

**Keywords:** antimicrobial, proteome, silver, *Pseudomonas*, *Staphylococcus*

## Abstract

The urgent need to combat antibiotic resistance and develop novel antimicrobial therapies has triggered studies on novel metal-based formulations. *N*-heterocyclic carbene (NHC) complexes coordinate transition metals to generate a broad range of anticancer and/or antimicrobial agents, with ongoing efforts being made to enhance the lipophilicity and drug stability. The lead silver(I) acetate complex, 1,3-dibenzyl-4,5-diphenylimidazol-2-ylidene (NHC*) (SBC3), has previously demonstrated promising growth and biofilm-inhibiting properties. In this work, the responses of two structurally different bacteria to SBC3 using label-free quantitative proteomics were characterised. Multidrug-resistant *Pseudomonas aeruginosa* (Gram-negative) and *Staphylococcus aureus* (Gram-positive) are associated with cystic fibrosis lung colonisation and chronic wound infections, respectively. SBC3 increased the abundance of alginate biosynthesis, the secretion system and drug detoxification proteins in *P. aeruginosa*, whilst a variety of pathways, including anaerobic respiration, twitching motility and ABC transport, were decreased in abundance. This contrasted the affected pathways in *S. aureus*, where increased DNA replication/repair and cell redox homeostasis and decreased protein synthesis, lipoylation and glucose metabolism were observed. Increased abundance of cell wall/membrane proteins was indicative of the structural damage induced by SBC3 in both bacteria. These findings show the potential broad applications of SBC3 in treating Gram-positive and Gram-negative bacteria.

## 1. Introduction

The emergence of drug-resistant pathogens necessitates the development of novel antimicrobial agents with alternative modes of action to conventional antimicrobial agents [1]. The rise in drug resistance has been caused partly by the incorrect use of treatments, and this has led to reduced efficacy against Gram-positive and Gram-negative bacteria [2]. β-lactam derivatives such as cephalosporins, monobactams and carbapenems show enhanced broad-spectrum activity and have tackled the growing rates of drug resistance to a certain extent [3]. β-lactam antibiotics target the synthesis of peptidoglycan—a meshwork of polysaccharide strands and peptides that maintain cell structure and rigidity [4]. Gram-positive bacteria (such as *Staphylococcus aureus*) possess an inner cytoplasmic membrane and thick peptidoglycan layer functionalised with teichoic acids. These anionic glycopolymers play roles in regulating cell morphology and division, cell adhesion and defence against temperature, osmotic and toxic stresses [5]. Gram-negative bacteria (such as *Pseudomonas aeruginosa*) have a thin peptidoglycan layer sandwiched between an inner cytoplasmic membrane and outer lipopolysaccharide membrane. The latter component offers osmo-protection, regulating the permeability of the cell and the influx and efflux of nutrients and toxins, whilst the presence of outer membrane proteins, or porins, creates channels that permit the passage of molecules in and out of the cell [6]. Decreased production and/or mutations of porins reduce permeability and prevent the influx of drugs, which can confer resistance. Mutations in the expression of the substrate-specific porin, OprD, contribute to carbapenem resistance, which is enhanced when combined with increased efflux system expression [7]. In contrast, Gram-positive bacteria have two main resistance strategies through β-lactamase enzyme production to degrade the β-lactam ring and/or mutations of the drug target site—penicillin-binding proteins (PBPs) [8].

A decrease in antibiotic discovery since the 1970s has encouraged the search for novel therapeutic candidates [9]. *N*-heterocyclic carbene (NHC) complexes possess broad-spectrum activity against a range of bacterial, fungal and viral pathogens, including SARS-CoV-2 [10,11]. These versatile ligands offer stability, enhance bioavailability and are compatible with a variety of metals to create agents with antimicrobial and/or anticancer properties [12]. The antimicrobial activity of the NHC* metal complex 1,3-dibenzyl-4,5-diphenylimidazol-2-ylidine silver(I) acetate (SBC3) synthesised by the Tacke group has previously demonstrated in vitro and in vivo [13]. *Escherichia coli* thioredoxin reductase is an SBC3 target and contributor in deregulating redox homeostasis [14]. The antimicrobial properties of silver are well recognised and have been exploited for centuries [15]. Silver sulfadiazine, silver nitrate and colloidal silver are currently in use as topical skin treatments [16].

Proteomic analysis provides the enhanced identification of novel biomarkers with improved specificity and sensitivity, increasing our understanding of disease progression and, in this context, drug discovery, development and toxicity [17]. The identification and quantification of proteins in cells, tissues or whole organisms can provide an insight into the activity of novel therapeutics at a molecular level. Label-free proteomics is an attractive technique with reduced costs, sample preparation time and variability introduced by labelling techniques. An abundance of analytical software tools allows for the extensive interpretation of raw MS data. Here, liquid chromatography tandem mass spectrometry (LC MS/MS) was employed to gain an insight into the mode(s) of action of SBC3 and provide a comparison of the differential responses of Gram-positive and Gram-negative bacteria to SBC3.

## 2. Results

### 2.1. The Antimicrobial Effect of SBC3

**Figure 1:**
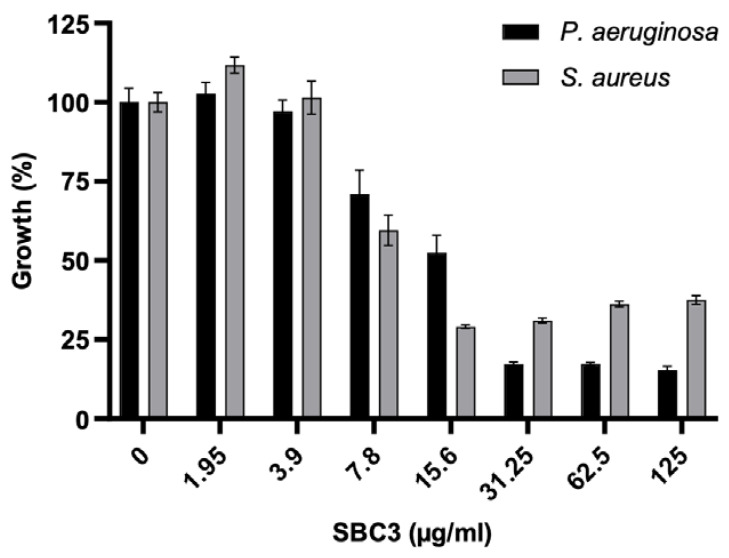
Evaluation of the effect of SBC3 on the growth of *P. aeruginosa* and *S. aureus*. All values are the mean ± S.E. of three independent experiments.

Evaluation of the effect of SBC3 on *P. aeruginosa* and *S. aureus* revealed up to 88.5% and 72.7% growth inhibition at 31.3 and 62.5 μg/mL, respectively. SBC3 demonstrated good inhibitory activity against both pathogens, with higher potency against *P. aeruginosa* (Figure 1). Based on the results in Figure 1, two concentrations of SBC3 that partly inhibited bacterial growth (*P. aeruginosa* (7.5 μg/mL) and *S. aureus* (12 μg/mL)) were identified and these concentrations allowed for sufficient cell density for protein extraction after six hours, and for cells to demonstrate a response to the compound.

### 2.2. Characterisation of the Proteomic Response of Bacteria to SBC3

Label-free quantitative (LFQ) mass spectrometry was employed to characterise changes in the proteomes of SBC3-treated and untreated bacteria. *P. aeruginosa* and *S. aureus* were exposed to the relevant SBC3 concentration for six hours, after which time proteins were extracted and analysed in order to determine the effect of SBC3 on the whole-cell proteomic response.

### 2.3. Characterisation of the Effect of SBC3 on the Proteome of P. aeruginosa

**Figure 2:**
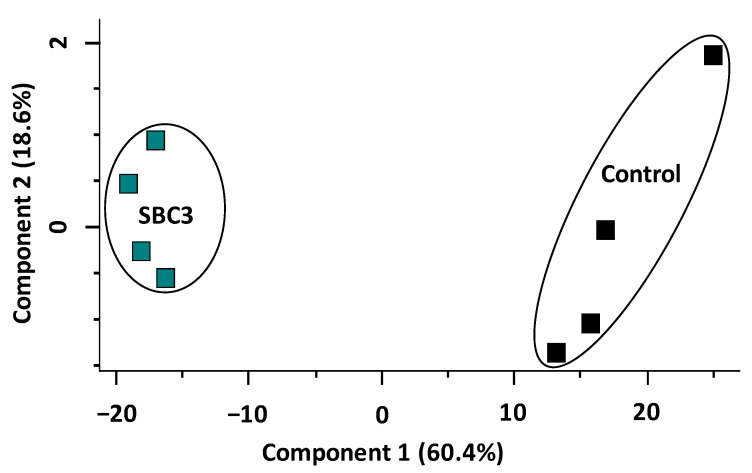
Principal component analysis (PCA) of SBC3-treated *P. aeruginosa* versus untreated control samples.

A total of 2526 *P. aeruginosa* proteins were identified initially, of which 1759 remained following the filtration of contaminants. The entire data set of filtered proteins is shown on the principal component analysis plot (PCA) (Figure 2), whereby all biological replicates (*n* = 4) are resolved into the corresponding sample groups. Distinct differences are evident between untreated and SBC3-exposed samples, with a combined variance of 79% resulting from Components 1 and 2. Subsequent two-sample *t*-tests (*p* < 0.05) were performed on 1759 proteins post-imputation and identified 362 statistically significant (*p* < 0.05) and differentially abundant (SSDA) proteins with a minimum fold change of 1.5.

**Figure 3:**
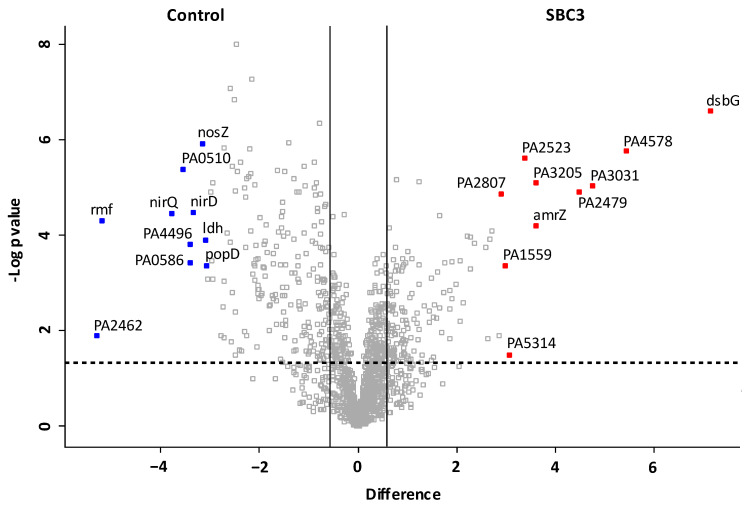
Volcano plot generated from two-sample *t*-tests showing the distribution of all identified proteins (post-filtration of contaminants). The *x*-axis denotes fold change (log2 LFQ intensity difference), and the *y*-axis denotes the *p* value (−log10 *p* value). Statistically significant proteins (*p* < 0.05) lie above the horizontal, dashed line, and proteins with a fold change of ≥1.5 are to the right and left of the vertical lines. The top ten most increased and decreased proteins in abundance are coloured red and blue, respectively. The corresponding gene names are annotated, and the protein products and corresponding functions are listed in Table 1.

The distribution of all filtered proteins (1631) is represented on a volcano plot (Figure 3) conducted via a pairwise Student’s *t*-test (*p* < 0.05), and the top ten most differentially abundant proteins (±fold change ≥ 1.5) are highlighted and annotated with associated gene names. The protein products are listed in Table 1. Thiol:disulfide interchange protein DsbG (+142-fold), transcription factor Amrz (+12-fold) required for environmental adaptation and probable two-component response regulator (+10-fold) for proteolysis and biofilm formation were increased in abundance. Haemagg_act domain-containing protein (−39-fold), ribosome modulation factor (−37-fold) and probable binding protein component of ATP-binding cassette (ABC) transporter (−11-fold) were decreased in abundance.

### 2.4. Characterisation of the Effect of SBC3 on the Proteome of S. aureus

**Figure 4:**
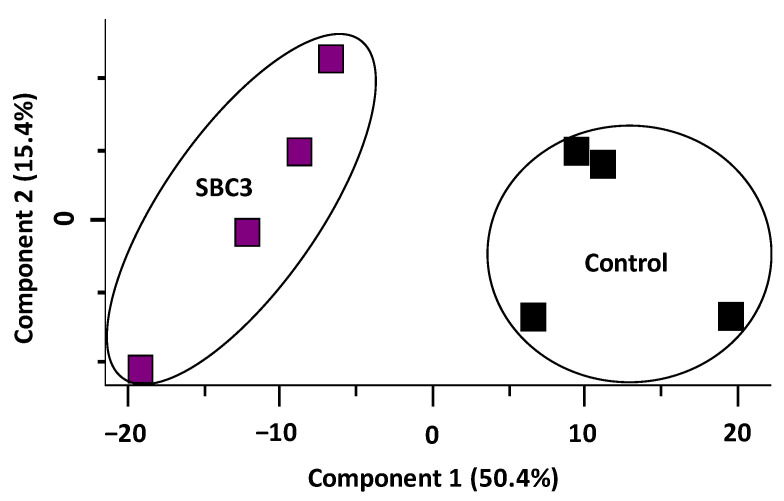
Principal component analysis (PCA) of SBC3-treated *S. aureus* versus untreated control samples.

Proteomic analysis of *S. aureus* treated with SBC3 identified 1271 proteins and 1183 filtered proteins. The PCA plot (Figure 4) summarises this entire data set, showing the contrast between untreated and SBC3-treated *S. aureus* proteomes. A combined variance of 65.8% arose from Components 1 and 2.

**Figure 5:**
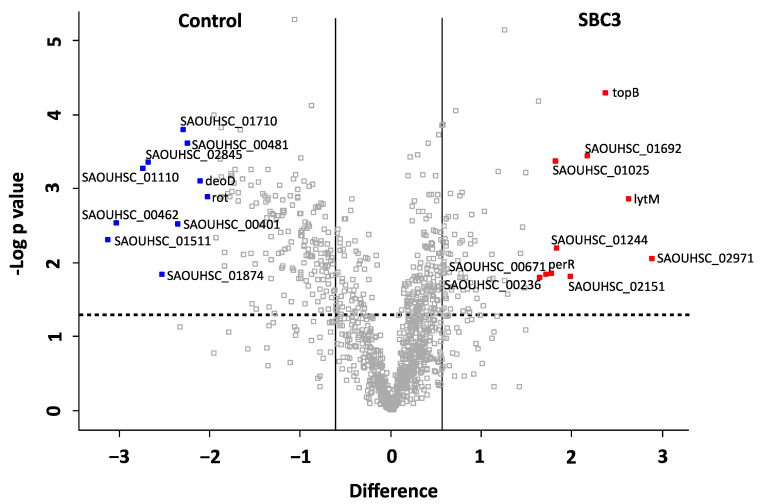
Volcano plot generated from a two-sample *t*-test (*p* < 0.05) of *S. aureus* treated with SBC3 (12 μg/mL) versus untreated control samples. All identified and filtered proteins are displayed based on *p* value (-log10 *p* value) on the *y*-axis and fold change (log_2_ mean LFQ intensity difference) on the *x*-axis. Statistically significant (*p* < 0.05) proteins are positioned above the horizontal, dashed line, and proteins with a fold change of ≥1.5 are to the right and left of the vertical lines. The top ten most differentially abundant proteins are highlighted and annotated with gene names, which are listed in Table 2, and including the protein products, functions and fold change.

A volcano plot was generated representing the distribution of all filtered proteins (Figure 5). The top ten most differentially abundant proteins are highlighted and annotated by the corresponding gene names. The protein products and relative fold changes are displayed in Table 2. A metalloproteinase (+7.4-fold) and proteins associated with cell wall organisation (glycyl-glycine endopeptidase LytM; +6.2-fold, probable autolysin SsaALP; +3.3-fold), DNA replication (DNA topoisomerase 3; +5.2-fold), ABC-type transporter activity (ABC transporter permease; +4-fold) and oxidative stress defence (peroxide-responsive repressor PerR; +3.4) were increased in abundance. Proteins associated with transcriptional regulation of virulence factors (HTH-type transcriptional regulator rot; −8.8-fold), deoxyribonuclease activity (hydrolase TatD; −8.2-fold) and cell redox homeostasis (thioredoxin domain-containing protein; −4.1-fold) were decreased in abundance.

### 2.5. Interaction Network Analysis on the Response of P. aeruginosa and S. aureus to SBC3

Protein interaction networks generated via STRING incorporated SSDA proteins derived from pairwise *t*-tests. Individual nodes representing proteins (annotated with the corresponding gene name or STRING identifier) are connected by lines denoting an interaction and the width of the line denotes confidence i.e., the strength of data support.

**Figure 6:**
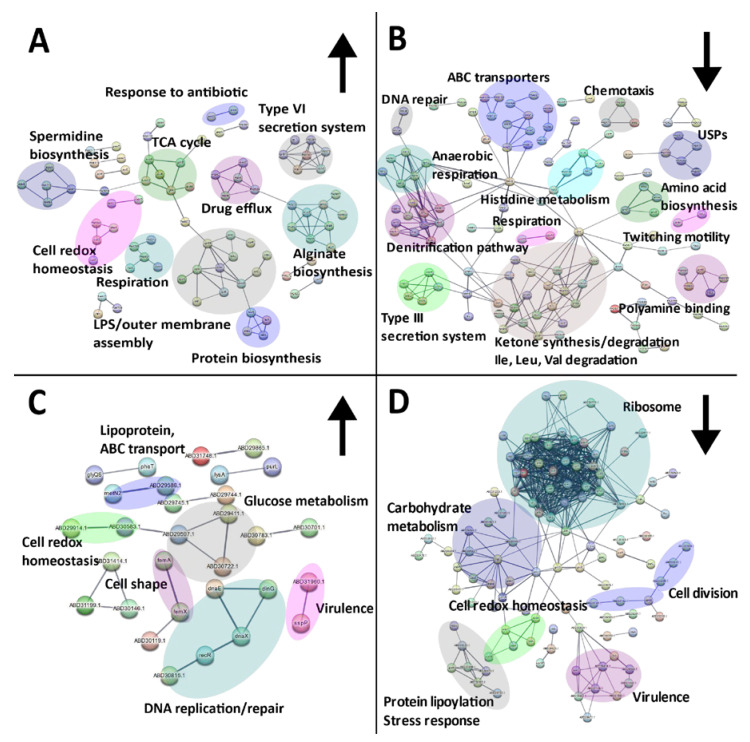
STRING network analyses of SSDA proteins derived from two-sample *t*-tests (*p* < 0.05) showing the responses of *P. aeruginosa* (**A**,**B**) and *S. aureus* (**C**,**D**) to SBC3. Nodes represent individual proteins (annotated with the corresponding gene name or STRING identifier) and are connected to represent an interaction. The thickness of the line represents the strength of data support. Interaction networks with proteins increased in abundance are displayed on the left (**A**,**C**) indicated by upward arrows and those decreased are shown on the right (**B**,**D**) indicated by downward arrows. Protein genes/IDs were searched via the UniProt database in addition to functional enrichment analysis on STRING to determine KEGG and Gene Ontology (GO) terms.

Exposure of *P. aeruginosa* to SBC3 resulted in 366 SSDA proteins with a log_2_ fold difference ranging from −5.2 to 7.2. Mapping of these proteins revealed a multitude of targeted protein pathways (Figure 6). Whilst some virulence and resistance mechanisms (alginate/spermidine biosynthesis, type VI secretion and drug efflux) were elevated (Figure 6A), a large proportion of clusters were decreased and these included ABC transporters, the type III secretion system, respiration and amino acid metabolism (Figure 6B).

*S. aureus* challenged with SBC3 generated 251 SSDAs with a log_2_ difference of −3.1 to 2.9. STRING protein clusters associated with cell wall organisation, cell redox homeostasis and DNA replication and repair were among the most elevated (Figure 6C). A substantial number of clusters, such as ribosome, lipoylation and glycolysis proteins, were decreased in treated samples (Figure 6D).

## 3. Discussion

*P. aeruginosa* and *S. aureus* are widespread nosocomial pathogens, displaying high levels of multidrug resistance [18]. Susceptible individuals are at risk of contracting chronic wound infections, sepsis, endocarditis and other infections introduced via indwelling medical devices (e.g., catheter-associated urinary tract infection) [19]. *P. aeruginosa* and *S. aureus* are the main causes of bacterial infection in cystic fibrosis (CF) patients. *S. aureus* typically precedes *P. aeruginosa* in the earlier stages of disease; however, incidents of co-infection enhance morbidity and mortality rates [20]. Biofilm formation is common between these species and offers high levels of protection and resistance against host immune clearance and antimicrobial agents [21].

### 3.1. SBC3 Compromises Cell Structural Integrity in Both S. aureus and P. aeruginosa

The onset of resistance to conventional antibiotics has prompted the development of improved silver formulations for the treatment of drug-resistant pathogens [22]. Positively charged silver (Ag^+^) ions bind to negatively charged cell membranes and induce cell membrane/wall leakage and/or rupture [23]. The proteomics results presented here revealed significant alterations in the abundance of proteins associated with cell wall structure in *S. aureus*. For example, the peptidoglycan biogenesis and degradation protein, glycyl-glycine endopeptidase LytM, was increased +6.2-fold following exposure to SBC3. Acyltransferase enzymes (endcoded by the *femA* and *femX* genes) utilised in cell wall organisation and peptidoglycan biosynthesis were also elevated [24]. Alterations in the abundance of outer membrane proteins and the lipopolysaccharide layer in *P. aeruginosa* provide further evidence of the roles of SBC3 in disrupting the cell’s structural integrity. The increased abundance of outer membrane protein assembly factor BamB (+2.9-fold) and outer membrane protein assembly factor BamD (+1.8-fold) is indicative of a stress response [25], whilst an array of lipopolysaccharide proteins identified within a STRING cluster could be indicative of the cell’s response in decreasing membrane permeability and limiting the entry of noxious compounds [26,27].

Elevated abundance of SpeH (+2-fold) and SpeE2 (+1.9-fold) was a further indication of the role of silver in damaging cell structural integrity. This compliments previous studies where the treatment of *P. aeruginosa* PAO1 with sublethal concentrations of polymyxin B (an outer-membrane-damaging agent) resulted in increased expression of SpeE [28].

### 3.2. The Proteomic Effects of SBC3 on P. aeruginosa

#### 3.2.1. Translocation and Biofilm Formation

Chronic infection by *P. aeruginosa* in CF patients is characterised by phenotypic switching from a non-mucoidal to mucoidal phenotype and concurs with alginate over-production [29]. Proteins identified within the STRING cluster were among some of the most differentially abundant. The highly conserved transcription factor AmrZ (*amrZ*) and activator of alginate biosynthesis were increased +12.2-fold, whereas PA3205 (identified as LTXXQ domain protein via UniProt) was also increased +12.2-fold, and this is hypothesised to detect membrane alterations and commence adherence and biofilm formation on abiotic surfaces [30]. Other key regulators in alginate biosynthesis, such as sigma factor AlgU negative regulatory protein (*mucA*, +1.9-fold), sigma factor AlgU regulatory protein MucB (*mucB*, +1.5-fold), periplasmic serine endoprotease DegP-like (*mucD*, +1.6-fold), alginate biosynthesis transcriptional regulatory protein AlgB (*algB*, +1.6-fold) and phosphomannomutase/phosphoglucomutase (*algC*, +1.9-fold) are also located within this cluster.

The successful colonisation and establishment of mature biofilms depends on a chemosensory system mediated by pili for translocation towards favourable environments [31], both of which were reduced post-exposure to SBC3 (Figure 6). Detection of chemical gradients by chemotaxis initiates adherence, whilst the type IV pilus system in *P. aeruginosa* consists of cell surface appendages that elongate and retract to further enable adhesion, biofilm formation, twitching motility and DNA uptake [32,33]. Pil proteins can be categorised into four subcomplexes, including the outer membrane secretin pore complex (PilQ, +1.6-fold), the inner membrane alignment subcomplex (PilM, −2.3-fold) and the cytoplasmic motor subcomplex (PilU, −1.8-fold) (Figure 6/Supplementary Dataset S1B). The fourth subcomplex is the pilus itself, requiring PilA and minor pilins. The non-pilin protein PilY1, which showed a −1.8-fold reduction in abundance, has roles in pilus retraction and as a mechanosensory element, which, upon attachment, induces an acute virulence phenotype [31,34,35,36].

#### 3.2.2. Virulence

The type III secretion system of *P. aeruginosa* injects effector proteins into host cell cytoplasm and exerts cytotoxicity activity and suppresses host immunity [37,38,39]. One such effector, responsible for disrupting phagocytosis, is the secreted exoenzyme S (*exoS*), and this was decreased in abundance by −6-fold following SBC3 treatment [40]. Translocator protein PopB (−5.8-fold) and translocator outer membrane protein PopD (−8.4-fold) comprise the pore and are linked to the needle tip type III secretion protein PcrV [41]. Transcription anti-activator ExsD (−1.7-fold) is a negative regulator of the type III secretion system regulon [42].

Upregulation of secretion systems can arise in response to antibiotic-induced stress [43]. Components of the type VI secretion system such as the type VI secretion system sheath protein TssB1 (+4.8-fold), type VI secretion system sheath protein TssC1 (+6.5-fold), type VI secretion system baseplate component TssK1 (+2.3-fold) and protein Hcp1 (+3-fold), which is an effector protein whose secretion is aided by AAA+ ATPase ClpV1 (*clpV1*) (+2.2-fold), were increased in abundance [44].

#### 3.2.3. Anaerobic Respiration/Stress Response

Thick mucus in the CF lung can generate an anaerobic environment to which *P. aeruginosa* must adapt for survival [45]. Anaerobic growth is mediated by two main pathways, (1) arginine fermentation and (2) the denitrification pathway, whilst pyruvate fermentation is implemented in nutrient-deprived environments. Although growth and metabolism are halted, the latter sustains long-term survival [46]. Universal stress proteins (USPs), such as PA3309 (encoded by *uspK*) and PA4352 (*uspN*), have roles in pyruvate fermentation that are essential for anaerobic stationary phase survival. Both proteins were decreased in abundance in response to SBC3 (−4.5-fold and −4.8-fold, respectively). In addition, PA1789 (*uspL*), PA4328 (*uspM*) and PA5207 (*uspO*) proteins were decreased −4.9-, −6.3- and −5.5-fold, respectively, and all of these are induced in oxygen-depleted conditions [47]. Transcription of these genes is controlled by the global anaerobic regulator Anr (encoded by *anr*) and interestingly was increased +1.8-fold (gene and protein products are located in Supplementary File S1) [48].

Denitrification is a multistep process involving nitrate reduction to ultimate nitrogen gas formation [49]. Heme *d*_1_ is an isobacteriochlorin co-factor of the nitrite reductase, NirS, which catalyses the reduction of nitrite to nitric oxide [50]. Synthesis of heme *d*_1_ requires enzymes encoded by the *nir* genes [51,52,53], several of which (and their protein products) were decreased: *nirD* (siroheme decarboxylase NirD subunit, −10.1-fold), *nirE* (uroporphyrinogen-III C-methyltransferase, −11.7-fold), *nirG* (siroheme decarboxylase NirG subunit, −7.8-fold), *nirL* (siroheme decarboxylase NirL subunit, −3-fold), *nirN* (dihydro-heme d1 dehydrogenase, −4.1-fold), *nirQ* (denitrification regulatory protein NirQ, −13.8-fold) and *nirS* (nitrite reductase, −5.9-fold). Also shown are the enzymatic complex NarGHI nitrate reductase genes utilised in anaerobic respiration, *narG* (nitrate reductase (quinone), −2.8-fold), *narH* (respiratory nitrate reductase beta chain, −2.4-fold) and *narJ* (respiratory nitrate reductase delta chain, −2.1-fold), along with *dnr* (transcriptional regulator Dnr, −3.3-fold) [54]. The disruption of these growth and/or anaerobic survival strategies highlights the potential of SBC3 for the treatment of recalcitrant infections.

ABC transporters belong to a superfamily of proteins driven by ATP hydrolysis to facilitate cellular import and export. The uptake of di- and tripeptides for nitrogen sources is mediated by the DppBCDF system consisting of DppA1-5 substrate-binding proteins (in *P. aeruginosa* PA14), which play a role in biofilm formation and swarming ability [55]. Homologous proteins belonging to this system were decreased in abundance following treatment with SBC3 (PA4497/dppA7 and PA4496/dppA9; −10.6- and −2.9-fold, respectively). The high-affinity branched-chain amino acid transporters within the same cluster, including high-affinity branched-chain amino acid transport ATP-binding protein BraG (−1.5-fold) and leucine-, isoleucine-, valine-, threonine- and alanine-binding protein (part of the high-affinity branched amino acid transport system LIV-1; −1.8-fold), were also among the same cluster of ABC transporters. Branched-chain amino acids (Ile, Leu and Val) are fundamental nutrients for protein synthesis that not only support growth, but also environmental adaptation and virulence [56]. Interference with amino acid metabolism is also shown through the decreased abundance of proteins associated with amino acid catabolism via the *liu* gene cluster (Ile, Leu and Val degradation), in addition to ketone body regulation, which serves as an alternative metabolic fuel source [57].

Putrescine and spermidine are linked to a multitude of functions, such as virulence, biofilm formation and antibiotic resistance [28]. PotABCD polyamine transporters for putrescine and spermidine uptake were altered by SBC3 treatment (potA/PA0603, −1.8-fold and potD, −2.7-fold). Another ATP-binding component of the ABC transporter protein encoded by PA1807 was increased within this cluster by two-fold. The exact function of this protein in *P. aeruginosa* is unclear but, as part of the YejABEF ABC transporter system in *Brucella melitensis*, these proteins conferred resistance to polymyxin B [58].

#### 3.2.4. Detoxification Mechanisms

Exposure to SBC3 increased the abundance of multidrug resistance proteins MexA and B, belonging to MFP and RND, respectively, by +2.2-fold and +1.9-fold. *P. aeruginosa* has a homologous efflux system, MexCD-OprJ, including RND multidrug efflux membrane fusion protein MexC, +1.9-fold, and efflux pump membrane transporter (encoded by *mexD*), +1.8-fold [59,60].

The increased abundance of cell redox homeostasis proteins such as catalase (*kat B*, +3.7-fold), thioredoxin reductase (*trxB2*, +2.3-fold), alkyl hydroperoxide reductase C (PA0848, +1.9-fold) and alkyl hydroperoxide reductase subunit F (*ahpF*, +1.6-fold) is an indicator of a stress response. Interestingly, the most differentially abundant *P. aeruginosa* protein identified here was thiol:disulfide interchange protein DsbG (+142.1-fold), which is also required for the maintenance of cell redox homeostasis. It is possible that this increase is a damage control measure against misfolded proteins, which reflects previous findings in response to copper stress [61].

#### 3.2.5. Aerobic Respiration

The ability to adapt to hostile environments is dependent on adequate energy supplies. The tricarboxylic acid (TCA) cycle is the predominant energy source for cells as part of aerobic respiration [62]. SBC3 exposure increased the abundance of several TCA cycle proteins and decreased two cytochrome-c oxidases (ccoO2/ccOP1/N1) in *P. aeruginosa* (Figure 6).

### 3.3. The Proteomic Response of S. aureus to SBC3

#### 3.3.1. Protein Synthesis

Inhibition of protein synthesis can have detrimental effects on cell proliferation [63]. This, along with structural differences to eukaryotic ribosomes, has been exploited in conventional antibiotics (aminoglycosides, macrolides and tetracyclines) to inhibit protein synthesis; however, increasing rates of antibiotic resistance demand alternative options [64]. Functional enrichment analysis via STRING revealed a significant reduction in *S. aureus* ribosomal proteins necessary for transcription and translation following SBC3 exposure. This is consistent with previous studies that examined the proteomic response of *Candida parapsilosis* to SBC3 [65].

#### 3.3.2. Protein Lipoylation

The functioning of several metabolic enzymes relies on post-translation modification, namely lipoylation [66]. *S. aureus* can acquire lipoic acid via de novo synthesis or retrieval from their host under nutrient-deficient conditions in the environment. This important co-factor is required by enzyme complexes used for metabolism and host immune suppression [67,68]. One such complex is the glycine cleavage system (GCS), which catalyses the degradation of glycine and comprises four proteins: P, H, T and L proteins. Lipoic acid synthesis commences with the transfer of octanoic acid to GcvH via an octanoyltransferase LipM (Supplementary Dataset S2B). This mechanism is an adaptive strategy to overcome nutrient scarcity [69]. Within this downregulated cluster, glutamine synthetase (encoded by *glnA*) was also identified. This enzyme has multifunctional roles as a transcriptional co-regulator and chaperone in ammonium assimilation, in addition to growth and biofilm formation. This may open avenues as a novel therapeutic target [70].

#### 3.3.3. Glucose Metabolism

The role of silver in targeting the glycolytic enzyme glyceraldehyde-3-phosphate dehydrogenase in *E. coli* has been characterised [71]. Silver’s disruption of glyceraldehyde-3-phosphate dehydrogenase preceded more recent studies on the inactivation of glycolytic enzymes in *S. aureus* in response to silver [72]. Treatment of *S. aureus* with SBC3 increased the abundance of some glycolytic enzymes (glucokinase and alpha amylase family and 6-phospo-beta-glucosidase proteins, putative), while a larger proportion of proteins associated with the pentose phosphate and glycolysis pathways was decreased. It is important to note that glucose-derived energy is essential for *S. aureus* (and other pathogens) to establish infection [73].

#### 3.3.4. Cell Redox Homeostasis

*S. aureus* challenged with a higher dose of SBC3 (21.1 μM) to *P. aeruginosa* proved more effective in disrupting the oxidative stress response. Whilst thioredoxin reductase and YpdA family bacillithiol disulfide reductase were elevated in abundance, thioredoxin, catalase, superoxide dismutases and alkyl hydroperoxide reductase C were significantly reduced in abundance.

## 4. Materials and Methods

### 4.1. Bacterial Culture Conditions

*P. aeruginosa* PAO1 and *S. aureus* ATCC 33591 cultures were grown in nutrient broth (Oxoid, Basingstoke, UK) at 37 °C in an orbital shaker at 200 rpm. Bacterial stocks were maintained on nutrient agar at 4 °C.

### 4.2. Antibacterial Susceptibility Assays

Bacterial cultures were grown overnight in nutrient broth (Oxoid, Basingstoke, UK) in an orbital shaker at 200 rpm at 37 °C. SBC3 (1 mg/mL) was dissolved in sterile nutrient broth with 5% dimethylsulfoxide (DMSO; Honeywell, SLS Scientific Laboratory Supplies, Ireland Ltd). Serial dilutions of the complexes were performed in 96-well plates (Corning^®^, Somerville, MA, USA) containing nutrient broth (100 μL/well) to give a concentration range of 0.49–125 μg/mL. The optical density (OD) of overnight cultures was measured at 600 nm (OD_600_) and cell suspensions were adjusted to 0.01 in nutrient broth. Aliquots of cells (100 μL) were added to each SBC3 concentration to obtain a final cell density of approximately 1 × 10^7^ cells/well (*S. aureus*) and 3 × 10^5^ cells/well (*P. aeruginosa*) (DMSO was added to untreated control samples at an equivalent concentration to the highest treatment dose (0.625%)). Plates were incubated in a static incubator at 37 °C for 18 h and read at 600 nm in a plate reader (Bio-Tek Synergy HT, Mason Technology, Dublin, Ireland) to measure bacterial growth.

### 4.3. Proteomic Analysis of P. aeruginosa Treated with SBC3

Sterile nutrient broth was inoculated with *P. aeruginosa* (~9 × 10^6^ CFUs/mL) and *S. aureus* (~7 × 10^8^ CFUs/mL) and grown in the presence of SBC3 (7.5 μg/mL and 12 μg/mL, respectively) at 37 °C in an orbital shaker at 200 rpm for 6 h until the mid-exponential growth phase. Proteins were extracted, digested and purified as described [64]. Dried samples were resuspended and loaded onto a Q Exactive Mass Spectrometer (2 μL containing 750 ng of protein) using a 133 min reverse phase gradient.

Protein quantification and label-free quantification (LFQ) normalisation were processed through MaxQuant software version 1.6.3.4 using *Staphylococcus aureus* NCT8325 (downloaded 12 October 2021; 2889 entries) and *Pseudomonas aeruginosa* PAO1 (downloaded 9 July 2021; 5564 entries) databases following previously described search parameters [74]. The mass spectrometry proteomics data have been deposited to the ProteomeXchange Consortium via the PRIDE [75] partner repository with the dataset identifier PXD038616.

### 4.4. Data Analysis

Statistical and graphical analyses were carried out by processing the resulting LFQ values generated via MaxQuant through the statistical software tool Perseus v.1.6.6.0, with all parameters set in accordance with previous methods [64].

The Search Tool for the Retrieval of Interacting Genes/Proteins (STRING) v11.5 www.string-db.org/ (accessed on 1 November 2022) was used to map statistically significant and differentially abundant (SSDA) proteins using their corresponding gene names retrieved from UniProt gene lists for *P. aeruginosa* PAO1 and *S. aureus* NCTC 8325. A high confidence score (0.7) was used to generate protein/protein interaction networks for treatment versus control sample groups. Disconnected nodes were omitted. Functional enrichment analysis was carried out along with manual searches using the UniProt database to determine individual protein and pathway functions.

## 5. Conclusions

Exposure of *S. aureus* and *P. aeruginosa* to SBC3 resulted in the inhibition of growth but differential proteomic responses. Both cell types showed alterations in the abundance of proteins associated with the cell wall or envelope. However, in *P. aeruginosa*, a multitude of pathways were affected, including alginate biosynthesis, secretion systems, drug detoxification and anaerobic respiration. This contrasted with the response of *S. aureus*, where pathways such as protein synthesis, lipoylation, glucose metabolism and cell redox homeostasis were affected. The results presented here demonstrate the broad-spectrum activity of SBC3 against Gram-positive and Gram-negative bacteria, with differing proteomic responses from both cell types. SBC3 may have applications in the treatment of Gram-positive and Gram-negative mono- or dual infections.

## Figures and Tables

**Table 1:** The top 20 differentially abundant proteins identified in SBC3-treated *P. aeruginosa*.

Gene Name	Protein Name	Protein ID	Function	Fold Change
dsbG	Thiol:disulfide interchange protein DsbG	Q9I106	Disulfide bond formation	+142.1
PA4578	Uncharacterised	Q9HVK6	Unknown	+43.7
PA3031	Uncharacterised	G3XCW5	Unknown	+27.1
PA2479	Probable two-component response regulator	Q9I103	DNA binding	+22.5
PA3205	Uncharacterised	Q9HZ35	Unfolded protein binding	+12.2
amrZ	Transcription factor AmrZ	G3XCY4	Transcription; transcription regulation	+12.2
PA2523	Probable two-component response regulator	G3XD16	Positive regulation of proteolysis; positive regulation of single-species biofilm formation on inanimate substrate	+10.4
PA5314	Cupin_3 domain-containing protein	Q9HTP0	Unknown	+8.4
PA1559	NAD_binding_4 domain-containing protein	Q9I3F8	Unknown	+7.9
PA2807	Azurin	Q9I036	Copper binding; electron transfer activity	+7.5
popD	Translocator outer membrane protein PopD	Q9I323	Chaperone binding; type III secretion system	−8.4
ldh	Leucine dehydrogenase	Q9HYI7	Cellular amino acid metabolic process; oxidoreductase	−8.6
nosZ	Nitrous-oxide reductase	Q9HYL2	Oxidoreductase; denitrification pathway	−8.9
nirD	Siroheme decarboxylase NirD subunit	P95412	Lyase activity; denitrification pathway	−10.1
PA0586	Uncharacterised	Q9I5V1	Unknown	−10.5
PA4496	Probable binding protein component of ABC transporter	Q9HVS5	Peptide transmembrane transporter activity	−10.6
PA0510	Uroporphyrinogen-III C-methyltransferase	G3XD80	Methyltransferase; cobalamin biosynthesis; porphyrin biosynthesis	−11.7
nirQ	Denitrification regulatory protein NirQ	Q51481	Transcription; transcription regulation	−13.8
rmf	Ribosome modulation factor	Q9HZF9	Translation regulation	−36.7
PA2462	Haemagg_act domain-containing protein	Q9I120	Macromolecule metabolic process; primary metabolic process; nitrogen compound metabolic process	−39.4

**Table 2:** The top 20 most differentially abundant proteins identified in *S. aureus* treated with SBC3.

Gene Name	Protein Name	Protein ID	Function	Fold Change
SAOUHSC_02971	Neutral metalloproteinase	Q2FUX4	Metal ion binding; protease	+7.4
lytM	Glycyl-glycine endopeptidase LytM	O33599	Cell wall biogenesis/degradation; virulence	+6.2
topB	DNA topoisomerase 3	Q2FW03	DNA topological change	+5.2
SAOUHSC_01692	CMP/dCMP-type deaminase domain-containing protein	Q2G2A1	Metal binding	+4.5
SAOUHSC_02151	ABC transporter permease	Q2FWX0	ABC-type transporter activity	+4.0
SAOUHSC_01244	YlxR domain-containing protein	Q2G2D1	Unknown	+3.6
SAOUHSC_01025	Membrane spanning protein	Q2FZH9	Unknown	+3.5
perR	Peroxide-responsive repressor PerR	Q2G282	Transcription of antioxidant-encoding proteins (katA, trxB, bcp, ahpCF)	+3.4
SAOUHSC_00671	Probable autolysin SsaALP	Q2G0D4	Cell wall biogenesis/degradation	+3.3
SAOUHSC_00236	6-phospho-beta-glucosidase, putative	Q2G1A9	Carbohydrate catabolic process	+3.1
SAOUHSC_02845	Thioredoxin domain-containing protein	Q2FV89	Cell redox homeostasis	−4.1
deoD	Purine nucleoside phosphorylase DeoD-type	Q2FWB8	Glycosyltransferase	−4.3
SAOUHSC_01874	Rhodanese domain-containing protein	Q2FXH3	Unknown	−4.7
SAOUHSC_01710	Acetyl-CoA carboxylase, biotin carboxyl carrier protein, putative	Q2FXX0	Unknown	−4.9
SAOUHSC_01511	Uncharacterised protein	Q2FY81	Unknown	−5.1
SAOUHSC_01110	Fibrinogen-binding protein	Q2FZC2	Complement binding	−5.8
SAOUHSC_00481	S4 domain-containing protein	Q2G0R5	RNA binding	−6.4
SAOUHSC_00401	Myeloperoxidase inhibitor SPIN	Q2G0X2	Host immune evasion	−6.7
SAOUHSC_00462	Hydrolase TatD	Q2G1S2	Deoxyribonuclease activity	−8.2
rot	HTH-type transcriptional regulator rot	Q9RFJ6	Negatively regulates the transcription of virulence factors (lipase, haemolysins, proteases). Positive regulation of cell surface adhesins.	−8.8

## Data Availability

The mass spectrometry proteomics data have been deposited to the ProteomeXchange Consortium via the PRIDE [75] partner repository with the dataset identifier PXD038616.

## Supplementary Materials

The following supporting information can be downloaded at: https://www.mdpi.com/article/10.3390/antibiotics12020348/s1, Supplementary dataset S1: All identified proteins in *P. aeruginosa* treated with SBC3.; Supplementary dataset S2: All identified proteins in *S. aureus* treated with SBC3.
