# Supplementary material for: Pseudomonas aeruginosa and Staphylococcus aureus Display Differential Proteomic Responses to the Silver(I) Compound, SBC3
Source: Antibiotics (Basel). 2023 Feb 8;12(2):348. doi: 10.3390/antibiotics12020348 (PMC9952281; doi:10.3390/antibiotics12020348)
Supplement: Supplementary file 1 [file antibiotics-12-00348-s001.zip › antibiotics-2175952-supplementary.pdf]

Supplementary dataset S1A. All identified proteins in *P. aeruginosa* treated with SBC3. All LFQ intensity replicates (rep.) are Log_2 transformed.

Protein name	Majority protein IDs	Gene name	Peptides	Sequence coverage [%]	Mol. weight [kDa]	Score	Intensity	MS/MS count	Sequence length	LFQ Intensity Control Rep. 1	LFQ Intensity Control Rep. 2	LFQ Intensity Control Rep. 3	LFQ Intensity Control Rep. 4	LFQ Intensity SBC3 Treatment Rep. 1	LFQ Intensity SBC3 Treatment Rep. 2	LFQ Intensity SBC3 Treatment Rep. 3	LFQ Intensity SBC3 Treatment Rep. 4
scriptional	G3XCU2	argR	8	41.6	36.854	34.972	4.92E+08	33	329	24.4782	23.9872	24.2421	24.4411	25.0127	25.1283	25.0573	25.0181
	G3XCW5	PA3031	2	35.6	8.0121	51.455	1.49E+09	13	73	23.1055	23.5233	22.6161	23.0348	28.4729	27.8122	27.732	27.6955
	G3XCW6	dnr	6	51.5	25.933	23.346	7.46E+08	20	227	25.6663	25.6685	25.5712	25.8873	24.1528	24.2318	22.9706	24.1863
	G3XCW7	PA1462	5	26	28.407	16.822	5.92E+08	35	262	25.0408	25.2075	25.6259	25.3799	24.5644	24.6521	24.3574	24.6538
	G3XCX1	narG	54	63.1	140.97	323.31	4.97E+10	446	1261	29.4995	29.5339	29.3235	29.4725	28.0877	27.7781	28.1243	27.8589
	G3XCX2	PA0624	5	66.7	12.523	78.617	9.68E+08	31	114	26.429	25.6516	25.1881	26.7667	23.8771	25.34	23.6904	23.332
	G3XCX3	pilU	14	52.9	42.532	79.43	8.69E+08	61	382	25.3424	25.3163	25.4802	25.0723	24.4442	24.4228	24.2897	24.7844
	G3XCX5	PA0618	4	22	31.958	18.797	3.15E+08	15	295	24.9256	24.9544	24.9527	24.949	23.5302	22.8535	23.2724	22.1102
	G3XCY4	amrZ	5	64.8	12.333	87.246	4.43E+09	34	108	24.6446	24.5881	23.5459	25.0051	27.981	27.851	28.595	27.7735
	G3XCY9	narJ	14	56.1	27.261	144.21	6.31E+09	93	246	28.1544	28.0031	28.046	28.3921	27.3179	27.1032	27.1534	26.8216
	G3XCZ4	PA0891	10	38.4	40.213	69.913	2.64E+09	59	370	26.0254	26.1787	25.3147	26.1006	26.9901	27.0574	26.9008	27.0284
	G3XCZ8	hemO	8	50	21.953	113.58	5.25E+09	69	198	26.2842	26.2507	25.3007	26.4529	27.873	27.585	29.4201	27.6895
	G3XD16	PA2523	7	40.2	25.788	47.514	1.01E+09	30	224	23.3823	23.5233	22.5262	23.6139	26.8558	26.5833	26.1642	26.4485
	G3XD20	mucD	22	58	50.321	316.56	4.7E+10	250	474	30.0562	29.8667	29.4574	29.8807	30.3772	30.7019	30.575	30.4108
	G3XD25	mexC	22	69	40.838	323.31	3.34E+10	192	387	28.1596	28.5207	27.7099	28.5525	29.3157	28.8959	29.0788	29.2924
	G3XD28	pilM	13	47.7	37.983	77.92	2.28E+09	61	354	26.121	26.425	26.6144	26.1469	25.7485	24.9251	24.8425	24.9719
	G3XD39	PA0622	14	58.5	41.209	165.49	2.14E+09	71	386	26.4523	26.5818	26.8572	26.2553	24.9802	24.8618	25.1235	25.0534
	G3XD40	PA3925	18	73.4	40.553	314.09	1.36E+10	196	391	28.4477	28.6217	28.6235	28.4528	27.7818	27.8053	27.8256	27.6786
	G3XD49	pcrV	6	34.7	32.283	37.315	4.43E+08	27	294	25.1918	25.5583	24.7379	25.2936	21.9624	22.4288	22.4284	23.193
	G3XD80	PA0510	11	67.7	29.654	130.14	3.36E+09	61	279	27.579	27.9327	27.6788	27.7741	23.9121	24.6441	23.7583	24.4602
	G3XD87	narH	36	78.4	58.105	323.31	2.67E+10	327	513	29.8729	29.8322	29.6535	29.7883	28.6742	28.3753	28.5599	28.3677
	G3XD93	PA3842	2	22.4	13.157	21.718	1.01E+09	19	116	26.887	25.8216	25.0273	27.1923	22.1913	24.9836	23.7427	24.8584
	G3XDA1	exoS	11	45	48.302	94.738	7.93E+08	30	453	25.8296	26.1813	26.1473	25.9096	23.2925	23.0381	22.5173	23.4628
-binding pr	G3XDA8	pstS	16	72.8	34.473	201.98	6.94E+09	118	323	26.9568	27.2493	25.5384	26.9009	28.0384	27.8171	27.6466	27.3905
moyltransf	P08308	arcB	23	76.8	38.108	308.42	3.06E+11	541	336	32.8033	32.9313	33.1026	32.6848	30.7432	30.4517	30.9536	30.4667
phatic amid	P11436	amiE	10	39.3	38.494	96.328	7.11E+08	43	346	25.0269	25.8158	24.8594	25.1212	23.5386	23.1654	22.9587	23.6347
membrane	P13794	oprF	14	51.7	37.639	173.46	1.8E+10	110	350	29.3568	29.6381	28.9615	29.6052	30.5408	29.9771	29.8971	30.1766
nine deimi	P13981	arcA	39	76.6	46.435	323.31	5.03E+11	822	418	33.4859	33.5383	33.8176	33.3219	31.5189	31.2599	31.5316	31.1306
bamate kin	P13982	arcC	19	53.9	33.08	274.55	1.46E+11	219	310	32.5664	32.5131	32.4283	32.5525	30.0217	29.7994	30.1004	29.7836
rate syntha	P14165	gltA	20	73.6	47.694	165.6	1.6E+10	192	428	27.9801	28.3086	27.4605	28.2249	28.7898	28.7139	28.6467	28.9949
me c551 pe	P14532	ccpA	16	66.5	37.403	160.22	2.63E+10	140	346	30.725	30.4261	30.4868	30.6641	28.1208	27.545	27.7689	27.9882
synthase c	P20580	trpE	13	38.8	54.555	82.417	1.06E+09	51	492	24.4884	24.8932	24.3344	24.345	25.1847	25.105	25.2081	25.4321
threonine-	P21175	braC	24	75.6	39.769	323.31	1.02E+11	352	373	31.3977	31.5576	31.446	31.3515	30.5926	30.4749	30.5973	30.5666
no acid tra	P21630	braG	8	50.2	25.591	114.74	2.13E+09	60	233	26.1586	26.3874	26.457	25.9404	25.769	25.7321	25.5847	25.4933
nscriptiona	P23747	algB	17	55.9	49.323	123.37	2.56E+09	67	449	25.5894	25.4173	25.6267	25.3316	25.8544	26.1115	26.4832	26.3084
nal activato	P23926	anr	9	38.1	27.129	66.696	2.75E+09	55	244	25.7994	25.8534	25.4536	25.8993	26.7929	26.7834	26.4895	26.482
rite reducta	P24474	nirS	37	69.4	62.653	323.31	6.75E+10	345	568	30.3261	30.6356	30.9052	30.2525	28.1113	27.8898	27.9244	27.9748
utase/pho	P26276	algC	24	57.7	50.295	277.26	1.06E+10	149	463	26.6574	26.7222	26.7898	26.4761	27.377	27.4641	27.8933	27.6385
expression-	P27017	amiC	10	48.6	42.807	41.431	6.55E+08	39	385	24.9835	25.0084	25.8822	25.038	24.0599	23.6342	22.4504	22.9448
erase sigma	P29248	fliA	10	58.7	27.522	90.733	5.76E+09	58	247	28.9885	28.8869	28.7077	29.0571	28.4917	28.1286	28.3967	28.0995
protein asse	P33641	bamD	8	39.6	38.615	35.046	6.27E+08	34	341	23.7194	24.0123	23.6651	24.2278	24.9989	24.4951	24.766	24.7529
ssembly pr	P34750	pilQ	23	40.1	77.378	166.32	3.72E+09	119	714	25.3122	25.5456	25.6516	25.3905	26.331	26.1814	26.0384	26.2143
negative r	P38107	mucA	10	72.7	20.998	185.91	2.7E+09	44	194	26.2144	26.6911	26.315	26.3613	27.4027	27.1538	27.4087	27.2234
U regulato	P38108	mucB	18	68	34.572	205.27	9.88E+09	141	316	26.9278	27.2521	27.1819	26.971	27.6053	27.543	27.9458	27.6014
anine-4-hy	P43334	phhA	8	48.5	30.321	63.31	2.14E+09	26	262	28.3761	28.1881	28.2642	28.0336	22.4816	26.8129	26.4059	26.0106
carbinolami	P43335	phhB	6	72	13.333	42.524	4.11E+09	31	118	28.5257	29.0924	28.2425	28.3037	28.2444	27.8783	27.3826	27.6965
hydrogena	P43904	aroE	12	52.6	29.484	67.534	3.11E+09	80	274	26.5338	26.8918	26.8248	26.733	26.0328	26.0297	26.049	25.8337
erase sigma	P45684	rpoS	11	44.9	38.235	155.27	4.1E+09	62	334	28.0109	27.5324	27.9635	27.5114	26.4787	26.057	26.3306	26.0787
Protein TolC	P50598	tolQ	6	28.6	25.282	46.68	8.96E+08	30	231	24.9604	25.257	24.2009	25.0595	25.9864	25.5887	25.7608	25.6649
esistance pr	P52002	mexB	12	20.9	112.79	44.063	5.61E+08	38	1046	23.2363	23.7424	23.2549	23.9439	25.149	24.5582	24.2152	24.8854
esistance pr	P52477	mexA	13	57.2	40.969	114.1	1.73E+09	61	383	25.1057	25.7881	24.732	25.648	26.7456	26.2721	26.2597	26.5104
coproporp	P77915	hemN	14	43	52.488	167.68	6.13E+09	73	460	28.1824	28.2765	28.4006	28.2575	25.8727	26.3863	26.0379	26.0127
Protein NirD	P95412	nirD	5	44	17.017	42.699	9.85E+08	24	150	26.424	26.683	26.3108	26.3935	23.0064	24.2535	23.2835	21.7359
Protein NirL	P95413	nirL	9	46	20.21	35.761	4.37E+08	14	174	24.7589	25.2969	25.2045	25.0244	22.567	22.1425	22.7266	24.178
Protein NirG	P95414	nirG	3	44.2	16.552	34.307	4.83E+08	15	147	26.1544	25.7946	25.5114	26.6424	22.7731	23.2698	22.9172	23.1221
Pyocin-S2	Q06584	pys2	4	9	73.853	16.727	1.88E+08	15	689	24.5943	23.1314	24.9739	24.4232	23.0672	22.418	23.0389	23.3228
	Q14T72	fdnG	9	15.2	114.16	37.207	3.23E+08	30	1026	23.995	24.1579	24.2173	23.9653	22.6169	23.6023	22.3396	23.3597
	Q14T74	pntAA	14	63.4	38.682	220.25	3.36E+09	66	372	26.7124	26.9285	27.1208	26.5662	26.151	26.1407	26.2036	26.0234
NA methyl	Q51385	rlmN	13	46.2	41.7	62.383	2.29E+09	67	379	26.1676	25.9653	25.8925	25.9496	26.5516	26.8297	26.4018	26.8008
ost factor s	Q51472	ihfA	4	41	11.471	14.766	1.9E+09	26	100	26.8855	26.2725	27.3028	26.7586	26.3487	26.0142	26.3219	25.5144
regulatory	Q51481	nirQ	15	72.3	28.904	236.82	5.16E+09	70	260	27.3374	27.9644	28.279	27.0535	23.5177	23.5457	24.0947	24.3343
Catalase	Q59635	katB	13	40.5	57.13	59.239	8.49E+08	48	513	23.9917	22.8161	23.7589	22.6582	25.6116	25.4628	25.2836	25.403
osyl)anthra	Q59649	trpF	4	38.4	22.298	25.496	7.38E+08	37	211	25.3481	24.2182	24.6524	25.3781	25.7929	25.567	25.579	25.5156
	Q9HT28	PA5546	13	50.5	44.096	87.159	3.09E+09	90	394	27.7741	26.9124	27.4395	27.7976	25.7463	25.3308	25.5695	25.689
	Q9HT45	PA5528	9	62	30.414	88.12	1.84E+09	54	284	24.9379	25.2078	24.2555	25.1945	26.74	26.5488	26.4348	26.9178
	Q9HT75	PA5498	4	19.2	33.608	62.772	7.58E+08	28	307	25.7134	25.6908	25.7127	25.7556	24.7862	24.7809	24.9837	24.9825
	Q9HT76	nrdJa	12	28.1	82.74	78.742	1.11E+09	44	734	25.6236	26.1859	25.4535	25.9003	25.2571	24.6087	23.5494	24.8114
	Q9HT77	nrdJb	12	73.4	24.885	103	3.41E+09	103	229	26.7526	26.8825	26.8747	26.6952	25.6225	25.6494	25.9038	25.6962
	Q9HT95	PA5475	11	73.1	21	198	2.14E+10	85	186	31.2623	30.7736	30.9107	31.117	27.6484	27.883	28.4016	28.3255
	Q9HTB1	PA5459	7	42.1	30.701	21.549	4.73E+08	23	278	24.8134	24.5975	24.9871	25.008	23.5698	24.3598	24.6517	23.7113
	Q9HTC6	PA5440	6	19.2	51.76	17.788	3.8E+08	19	464	25.2061	25.5049	25.2931	24.9301	23.5761	24.6173	23.4298	22.7809
	Q9HTD1	PA5435	38	69.2	66.095	323.31	3.89E+10	361	607	28.8524	28.8624	29.0636	28.5504	28.2042	28.1069	28.2088	28.0654
	Q9HTD9	adhA	20	62.6	35.911	255.33	7.97E+10	248	342	31.0622	31.1646	31.4474	30.9076	28.7393	28.5778	28.9762	28.4658
	Q9HTI6	PA5378	11	56.7	34.427	108.07	2.85E+09	85	312	26.0493	26.3611	26.6499	26.0697	25.0258	24.9521	25.2947	24.9823
orismate py	Q9HTK1	ubiC	12	80.9	19.512	79.176	3.38E+09	69	178	26.1337	26.313	25.9933	26.5968	27.1172	27.2162	26.8611	26.6074
ing protein	Q9HTL0	hupA	5	47.8	9.6951	77.018	6.18E+09	58	90	28.4739	28.3059	29.4799	28.388	27.7368	27.5345	28.0922	27.445
	Q9HTN7	PA5317	19	52.1	57.88	93.704	2.54E+09	82	526	26.5643	26.8301	26.9335	26.6251	26.2403	25.9285	26.3079	25.9147
	Q9HTP0	PA5314	2	35.8	13.395	39.264	1.27E+09	16	120	23.8691	23.2639	22.7811	27.4531	26.924	27.7578	26.8972	27.5591
acid dehydr	Q9HTQ0	dadA1	24	77.1	47.139	302.61	1.81E+10	210	432	28.6677	28.767	29.0421	28.642	27.8787	28.1947	28.0348	28.1569
	Q9HTU2	PA5252	17	36.5	70.871	55.971	9.2E+08	41	638	24.9993	25.1602	24.9935	24.6907	25.3068	25.5239	25.5932	25.9089
nponent of	Q9HTX3	PA5217	13	44.6	36.292	152.22	1.3E+10	138	332	26.6899	26.8987	27.058	26.7116	28.5883	28.2738	28.7907	28.2809
	Q9HTY2	PA5208	7	39.1	25.643	73.187	7.99E+08	34	225	25.5106	25.2162	26.4199	25.6305	23.6694	23.5023	23.2874	23.4787
	Q9HU06	PA5183	4	71.9	12.25	82.276	1.1E+09	30	135	24.6033	25.193	22.6114	24.9403	26.4192	26.3522	26.3907	26.43
	Q9HU11	PA5178	17	87.6	15.46	246.21	1.51E+10	139	145	27.6928	27.5565	27.7367	27.3058	28.6597	28.4749	29.1358	28.9425
	Q9HU32	PA5152	8	44.4	28.403	76.528	1.92E+09	44	257	26.314	26.294	26.6427	25.868	25.5467	25.5671	25.883	25.7316
	Q9HU45	PA5139	14	64.9	27.565	145.6	6.26E+09	89	248	27.8078	27.8693	27.6271	27.844	26.3466	26.4665	25.8439	26.2711
	Q9HU46	PA5138	9	48.4	27.915	69.011	2.43E+09	51	250	26.545	26.5013	26.5124	26.7017	25.7628	25.9295	25.5141	25.623
	Q9HU47	PA5137	6	41.8	27.991	35.443	5.01E+08	19	251	25.524	25.5375	24.7337	25.7071	23.2039	23.3632	24.1024	22.8437
	Q9HU69	PA5115	5	41.8	20.998	24.056	2.97E+08	19	194	24.2025	24.8683	25.0657	23.9772	23.4761	23.8045	23.5097	22.3541
utilization	Q9HU78	hutC	10	60	27.811	149.8	3.77E+09	88	250	27.1218	27.1995	27.4249	27.078	26.5732	26.4528	26.5902	26.5697
anate hydr	Q9HU83	hutU	25	56.4	61.193	246.64	8.15E+09	138	559	27.6559	27.5098	28.5073	27.4008	26.1854	25.6181	25.8055	25.7646
	Q9HU87	PA5096	9	39.4	35.496	47.231	1.1E+09	27	322	25.8787	25.7059	26.9974	25.6871	23.68	23.0897	22.9129	23.8786
oloneprop	Q9HU91	hutI	14	60.2	43.425	122.48	1.46E+09	60	402	25.9803	25.9389	26.9252	25.1081	23.2555	24.1451	22.4531	24.1326
	Q9HU92	hutG	11	65	29.823	62.312	1.21E+09	40	266	26.3608	26.39	26.8822	26.3808	25.0578	23.0853	25.2806	23.0322
	Q9HUA1	PA5082	9	37.1	32.866	41.676	3.43E+08	16	299	25.1758	24.0113	25.0703	23.3896	22.8508	22.7441	23.8319	22.5321
	Q9HUE2	PA5027	17	66.8	29.793	145.41	8.64E+09	95	271	28.6866	28.7132	28.694	28.5361	26.0834	26.172	26.2364	26.2591
	Q9HUE4	metY	14	49.9	45.177	167.34	5.49E+09	92	425	27.3862	27.4069	27.6679	27.2874	26.6554	26.5916	26.8901	26.6016
ne decarbo	Q9HUK8	psd	5	31.5	32.001	68.379	1.05E+09	27	289	26.1458	24.4239	25.3372	25.9663	26.5966	26.3391	26.414	26.8919
	Q9HUM2	hflK	14	54.8	44.044	237.09	3.62E+09	93	400	26.0258	26.3181	25.0087	26.344	27.0963	26.7078	26.6728	26.9379
Protein HflC	Q9HUM3	hflC	11	36.3	33.115	60.033	1.19E+09	48	289	24.3587	25.2901	24.6099	24.9508	25.8094	25.2046	25.4609	25.5102
bonuclease	Q9HUM7	rnr	21	32.7	100.15	74.338	1.39E+09	74	904	24.1306	24.5942	23.4162	24.6709	25.4678	24.9672	24.7972	25.2772
	Q9HUQ0	PA4913	7	29.1	39.798	27.23	1.72E+08	16	374	24.3987	24.7087	25.1731	23.6915	23.6299	23.1459	22.0374	23.0384
etate decar	Q9HUU1	PA4872	12	57.1	31.32	196.79	5.03E+09	89	287	27.1528	26.8532	26.5751	26.9686	27.7037	27.6055	27.5361	27.4775
	Q9HUW8	PA4842	6	20.2	39.989	20.283	4.03E+08	27	356	24.0518	24.4219	23.4377	24.1166	24.9433	24.4553	24.7284	24.6753
in FdhE ho	Q9HV00	fdhE	7	34	33.863	45.424	7.32E+08	23	309	25.4845	25.5573	24.6363	25.6837	24.685	24.9447	24.447	24.4777
	Q9HV06	PA4803	3	28.8	22.746	29.108	6.37E+08	16	205	23.1899	23.2224	24.5314	24.3939	25.884	26.2505	25.5098	26.3685
	Q9HV23	PA4785	9	42.8	45.9	55.248	5.78E+08	35	425	22.8314	23.7811	23.7699	23.2742	24.5496	24.8906	25.5955	24.573
	Q9HV27	PA4781	7	29	43.628	68.967	3.86E+08	26	393	25.065	25.0732	25.5362	24.6415	23.912	22.5252	23.1149	24.6336
	Q9HV33	PA4775	3	25.2	32.376	38.827	2.48E+08	13	286	23.9031	22.8524	22.5609	22.9974	23.2206	25.1229	24.3701	25.5628
minopropyl	Q9HV34	speE2	17	49.6	39.546	197.72	3.78E+10	175	349	29.7673	29.6044	28.957	29.5979	30.3075	30.5943	30.2097	30.4029
nine decarb	Q9HV35	speH	7	51.9	17.321	76.652	4.77E+09	57	160	26.8779	26.359	25.9978	27.2339	27.4144	28.0763	27.4821	27.8161
pair protei	Q9HV41	recN	5	17.7	60.7	26.406	3E+08	17	558	24.6659	24.734	24.1502	24.4571	22.6701	22.6829	24.5982	22.8919
glucosamin	Q9HV50	glmM	11	39.1	47.796	112.2	1.73E+09	69	445	25.1755	25.5165	25.2457	25.2434	25.9263	26.0036	26.0845	26.1712
oate hydro	Q9HV70	panB2	6	43.6	27.874	86.458	5.57E+09	45	266	28.0755	27.8854	26.8586	28.0897	28.8539	28.6794	28.6201	28.5676
tRNA(Asp)	Q9HV75	gluQ	7	34.8	32.507	30.587	3.84E+08	42	293	23.5352	22.8753	23.9161	23.9646	24.4966	24.2174	24.4595	24.0853
	Q9HV84	PA4714	6	70.5	15.779	39.271	1.09E+09	32	149	25.0776	23.6337	23.6651	24.5513	26.5931	27.2464	26.4889	26.9628
	Q9HV89	PA4709	10	37.3	39.057	92.146	1.41E+09	34	354	23.4636	23.338	23.0132	24.5293	25.7708	24.3049	28.0919	25.2119
	Q9HV93	cbpA	6	30.9	29.671	32.114	2.2E+08	15	265	24.0081	24.2171	23.9922	23.9567	23.6063	22.9177	22.7625	22.9217
	Q9HV95	PA4702	4	55.7	12.947	22.936	5.4E+08	17	115	26.1039	25.7842	24.9103	26.0354	23.6844	24.1069	22.0195	22.9989

tase catalyt	Q9HVA4	yedY	7	33.2	38.143	51.796	9.19E+08	41	337	25.0802	25.2492	25.1381	25.5712	24.901	24.6113	24.8036	24.1052
	Q9HVF2	PA4639	4	27.2	20.735	23.295	3.92E+08	21	195	23.9675	23.1625	23.175	24.36	25.5696	24.8238	25.0118	25.0974
	Q9HVH5	PA4616	15	63.6	36.959	152.59	7.14E+09	111	332	27.7769	27.8188	28.1983	27.893	26.4865	26.3306	25.9092	26.5362
phosphate	Q9HVI1	PA4608	4	47.2	14.573	50.619	6.5E+08	24	125	26.0701	25.9494	26.0349	25.9781	25.0775	24.6173	24.8431	25.1141
	Q9HVI2	PA4607	4	42.3	17.477	66.583	5.38E+08	22	168	25.4526	25.4115	24.8706	25.689	23.7722	22.8955	23.4088	22.0207
	Q9HVI9	mexD	16	28.6	111.59	230.42	7.46E+09	131	1043	26.5831	26.9002	25.9792	27.088	27.7399	27.3903	27.2303	27.6865
	Q9HVK6	PA4578	6	44.4	16.893	91.051	5E+09	38	162	23.3343	23.1915	23.0916	22.9586	28.7662	28.7744	29.2302	28.6787
	Q9HVK7	PA4577	4	57.4	11.756	70.168	2.93E+09	39	108	28.5134	25.5279	25.4914	28.0664	22.9989	25.3154	22.8031	24.3325
	Q9HVL3	PA4571	14	39.6	73.386	45.992	6.23E+08	41	675	24.6132	25.285	24.2872	25.6627	23.6706	23.3006	22.9833	22.7887
cine--tRNA	Q9HVM4	ileS	35	58.3	105.46	262.62	8.19E+09	226	943	25.7561	26.1493	25.1815	26.2658	26.8605	26.506	26.2306	26.652
biogenesis	Q9HVM8	pilY1	24	32.6	126.58	291.12	7.79E+09	156	1161	27.1585	27.3474	27.3359	27.1689	26.5154	26.3342	26.3179	26.3653
pe hydroxy	Q9HVQ7	piuC	5	31.9	25.616	27.207	4.71E+08	19	226	24.1019	24.3422	24.2364	23.8382	24.6297	24.7319	25.2261	24.9688
	Q9HVR9	PA4502	24	70.8	58.611	179.66	5.53E+09	143	531	27.0886	27.267	27.8567	26.863	25.1323	24.8768	25.0339	25.3083
	Q9HVS1	PA4500	19	41.8	59.674	81.963	1.23E+09	35	533	25.1736	26.216	27.5829	25.2356	23.399	24.0608	23.3171	23.266
	Q9HVS4	PA4497	11	44.9	59.496	80.469	5.61E+08	26	532	24.5777	25.1714	25.6225	24.4129	23.7611	23.0039	23.4027	23.5654
	Q9HVS5	PA4496	20	52.3	60.076	176.08	4.14E+09	85	537	27.1009	27.3372	28.3758	27.2183	24.9198	23.986	23.8125	23.6914
92 protein I	Q9HVT2	PA4489	25	29.9	167.43	116.32	1.48E+09	63	1516	24.9943	25.6206	25.7982	25.2025	26.0125	26.3195	26.8083	26.4219
	Q9HVV5	PA4463	8	67.6	11.739	153	8.54E+10	153	102	32.8816	32.4484	31.8925	32.9059	31.2211	30.9832	30.8793	31.2433
e c reducta	Q9HVY4	PA4431	9	69.5	20.828	67.609	1.35E+10	88	197	28.1194	27.734	26.4863	28.2692	28.9331	29.2243	28.306	29.3582
	Q9HW05	PA4401	6	37.4	23.789	16.234	5.5E+08	24	211	25.1104	25.0084	25.0779	25.0062	24.4121	24.4626	24.7358	24.1057
	Q9HW30	PA4372	13	57.9	38.323	57.19	1.29E+09	45	354	24.3219	24.796	23.4767	24.028	25.9611	25.4923	25.8643	26.2913
	Q9HW32	icmP	17	51.1	47.23	115.25	4.25E+09	102	446	25.9348	26.3745	25.4166	26.4622	27.1807	26.5861	27.0997	27.0108
	Q9HW37	PA4364	3	32.3	14.316	12.454	1.97E+08	8	133	24.7379	24.4882	24.4527	24.7959	23.7289	23.7602	23.0469	24.1632
	Q9HW49	PA4352	24	72.4	30.972	323.31	4.79E+10	277	286	30.2897	30.5554	30.7205	30.3055	28.2056	28.105	28.5218	27.9871
	Q9HW52	PA4349	7	33.9	31.671	41.701	1.39E+09	34	298	26.4564	26.4554	26.5608	26.2394	25.238	25.5529	25.8046	25.4559
	Q9HW53	PA4348	7	52.5	29.475	42.735	2.57E+09	33	261	28.1445	27.2484	26.8127	28.1302	25.6558	26.3158	23.5181	25.857
	Q9HW56	PA4345	7	50.5	22.161	28.116	4.95E+08	27	196	24.0897	24.1693	24.2044	23.8162	25.4012	24.957	26.0913	25.2345
	Q9HW73	PA4328	17	66.1	34.419	167.56	5.93E+09	120	304	27.1521	27.4689	27.7045	27.1596	24.9745	23.9809	25.1147	24.78
	Q9HWR5	PA4115	12	44.9	51.13	70.979	2.12E+09	65	461	25.7148	26.1	25.6332	25.5626	26.5574	26.4587	26.4448	26.6621
	Q9HWS8	PA4100	19	51.9	60.354	105.83	1.24E+09	90	559	25.18	25.7592	25.3808	25.3926	24.7181	24.4729	24.5457	24.7069
	Q9HWV5	PA4073	19	62.2	52.094	181.13	1.96E+10	186	495	29.9203	30.1167	30.2285	29.8164	29.1927	28.9608	29.1611	29.0752
	Q9HWX3	ribC	7	29.2	23.469	53.203	1.08E+09	35	219	25.8806	25.9064	26.0047	25.7352	24.1255	24.9499	24.738	24.9258
nonophosp	Q9HWX7	thiL	5	29.5	32.911	19.835	2.37E+08	13	322	23.6072	23.778	22.9515	23.0282	24.2164	24.5317	23.5679	24.3462
	Q9HX01	PA4026	5	38.6	18.049	22.014	9.61E+08	28	153	26.0466	26.057	26.5539	26.7287	26.0896	25.595	25.3325	25.7866
	Q9HX05;Q9I2xaC;PA402		22	56.9	54.979	323.31	2.34E+10	230	506	29.062	29.1626	29.6094	28.7079	27.8555	27.743	28.063	27.9726
	Q9HX12	PA4015	10	72.8	16.7	122.78	5.79E+09	76	151	27.1241	27.2954	27.3271	27.4985	26.8823	26.6668	26.6678	26.4253
yladenine I	Q9HX17	PA4010	8	49	26.949	45.375	1.11E+09	35	239	25.3141	25.7733	25.6141	25.3548	26.2919	26.5098	26.3227	26.2408
nbly lipopro	Q9HX32	lptE	5	31.9	22.883	29.354	6.75E+08	28	207	24.0109	24.4739	23.3007	24.6613	26.013	25.1848	25.5897	25.4134
	Q9HX76	PA3940	5	49.5	9.8923	87.376	7.25E+10	108	93	31.4442	31.3172	30.9321	31.7137	32.1306	32.0505	32.1217	31.8489
erized prote	Q9HX91	PA3922	23	57.1	51.229	320.83	9.12E+09	156	455	27.6429	27.751	28.6231	27.3379	26.1157	25.5564	26.1999	25.3179
	Q9HX94	PA3919	18	63.7	51.699	224.73	8.51E+09	112	463	28.5214	28.341	28.425	27.889	26.8522	26.6956	27.3909	26.8675
	Q9HXA0	PA3913	4	23.3	36.024	29.424	2.33E+08	16	331	23.9519	24.59	25.078	24.0675	24.2933	23.1905	22.7393	23.3795
f vertebrat	Q9HXB1	ivy	6	62.1	16.905	74.318	3.64E+09	46	153	27.4955	26.911	26.0702	27.5881	27.8077	28.334	27.7926	28.0911
	Q9HXC2	PA3891	6	36.2	43.303	62.391	5.8E+08	24	387	24.3278	24.1862	22.2273	23.3289	25.0151	25.2238	25.6405	25.1321
	Q9HXD2	PA3880	5	67.2	14.38	57.343	5.21E+08	21	131	25.4554	25.546	24.9406	25.6865	23.9634	23.9398	23.085	22.3885
	Q9HXD3	narL	11	68.9	24.255	113.2	3.98E+09	79	219	27.2841	27.0263	27.3875	27.3324	25.9896	26.059	26.0111	26.1879
ylprolyl isor	Q9HXD5	PA3871	9	37.1	30.13	206.49	2.55E+09	64	272	27.1419	26.7405	25.8684	27.2713	25.5565	25.4847	25.7552	25.7027
	Q9HXE1	PA3865	9	43.7	28.433	68.893	1.47E+09	47	263	25.755	25.9455	26.3109	25.9731	25.0057	24.8351	24.8467	24.3811
	Q9HXE8	PA3858	10	46.9	37.042	58.842	1.18E+09	54	341	25.6541	26.1431	25.7995	26.0448	25.3076	24.8192	25.3523	25.0258
	Q9HXG0	PA3846	4	37.2	19.892	17.412	4.74E+08	18	180	25.9194	25.9912	26.4858	25.9877	23.9668	24.4384	24.8622	23.3156
	Q9HXG8	PA3836	19	75.1	34.22	268.07	4.63E+10	207	325	30.1392	30.3997	30.1174	30.0235	29.4807	29.3919	29.3162	29.3923
ne--tRNA li	Q9HXH0	valS	31	44.4	107.71	286.26	9.34E+09	171	950	27.3074	27.0955	26.5641	27.2807	27.872	27.6116	27.5673	27.8553
	Q9HXI3	PA3819	13	80.2	18.972	234.24	1.34E+10	126	182	27.899	28.0215	27.175	28.0812	28.8224	28.2522	28.6832	28.361
ine--tRNA I	Q9HXJ5	hisS	13	39.9	47.512	162.68	5.76E+09	90	429	27.2303	27.0221	26.6251	27.0975	27.6505	27.6306	27.6174	27.5823
protein ass	Q9HXJ7	bamB	9	45.8	40.396	49.493	6.51E+08	31	380	23.6508	24.0284	23.0123	24.5118	25.8457	24.5811	24.9303	25.1581
	Q9HXK2	PA3795	9	50	34.803	113.74	4.4E+09	70	316	26.8486	26.638	27.026	26.7832	28.0655	27.6761	27.8536	27.5713
phosphate	Q9HXM5	guaB	21	61.1	51.707	259.66	3.18E+10	262	489	28.3094	28.399	28.0111	28.1448	28.6481	28.8787	28.858	28.9177
	Q9HXR8	PA3723	12	59.8	40.318	157.27	2.55E+09	49	368	26.6657	27.3444	27.5275	26.8169	24.1143	23.1194	24.357	24.0349
	Q9HXS8	spdH	25	50.3	68.861	150.75	3.04E+09	90	620	26.2248	26.3873	26.6822	26.0262	24.5483	24.3417	23.7896	25.0731
	Q9HXS9	PA3712	8	40.7	24.727	41.726	2.43E+09	33	231	26.9246	26.8755	26.5929	26.9248	27.9813	27.3177	27.4184	27.3187
	Q9HXU8	lptF	9	47.9	28.514	161.73	1.15E+09	44	261	23.9	24.8791	23.5687	24.5543	26.3434	25.5451	26.0664	25.6
	Q9HXV2	PA3688	4	42.1	12.918	58.219	8.24E+08	31	114	25.9096	25.9513	25.6975	25.6407	23.7344	22.7449	21.7191	23.1774
	Q9HXW5	PA3675	11	62.4	23.645	129.17	9.7E+09	76	226	28.3205	28.4203	27.3765	28.3884	28.9228	28.8719	28.8684	28.6932
	Q9HXY0	PA3659	8	36.1	44.544	47.531	1.27E+09	38	402	25.6528	25.1978	25.4059	25.7671	25.9833	25.8134	26.2325	26.4169
protein ass	Q9HXY4	opr86	18	39	88.286	114.42	3.07E+09	78	797	26.0371	26.2402	26.0136	26.202	26.9617	26.6276	27.0717	26.968
	Q9HY12	PA3614	16	62.5	51.921	96.393	1.78E+09	54	467	26.8789	26.7033	27.1318	26.5067	25.2015	25.7441	25.3431	25.7832
	Q9HY13	PA3613	22	46.8	88.378	132.23	2.44E+09	78	801	26.6116	27.1329	25.9359	27.0857	25.1829	24.1526	24.4925	25.0777
	Q9HY16	potD	20	80.2	39.26	147.52	6.81E+09	127	354	27.846	27.8736	27.8437	27.6649	26.2642	26.4538	26.3564	26.5089
binose-pho	Q9HY62	arnD	7	33.6	32.919	24.136	4.38E+08	22	295	24.2564	24.167	24.3667	24.2975	24.4652	24.8751	25.0307	25.2756
formyltran	Q9HY63	arnA	31	62.8	74.36	307.84	1.05E+10	230	662	26.8868	27.3336	26.448	27.2425	27.8916	27.5068	27.3057	28.1975
icterioferrit	Q9HY79	bfrB	6	65.8	18.553	41.83	1.57E+09	39	158	26.5785	26.4263	25.7419	26.7209	25.6813	25.4833	25.8766	25.6119
lglutathion	Q9HY85	gloA1	5	35.2	14.251	15.631	1.59E+09	25	128	27.0375	26.7679	27.4091	27.2514	26.451	26.257	26.237	26.3257
	Q9HYD6	PA3470	4	41.4	16.673	40.975	7.7E+08	14	152	22.7248	23.4105	23.406	25.8746	26.4661	26.4077	25.9421	26.1579
	Q9HYE8	PA3458	7	49	17.599	125.03	4.84E+09	51	157	28.2939	28.4802	28.3634	28.2066	26.8207	26.7928	26.7558	26.4461
	Q9HYH8	PA3427	7	37	32.323	50.322	7E+08	27	303	25.7607	25.8872	26.1341	25.4583	24.2255	24.9521	25.2143	24.9008
	Q9HYI7	ldh	10	54.5	35.633	142.28	1.57E+09	43	341	26.1187	26.7196	26.8248	25.6688	23.1438	23.697	22.6289	23.4618
	Q9HYK7	fpr	20	85.3	29.517	223.73	5.57E+10	256	258	29.7204	29.7204	29.2712	29.7573	30.4585	30.2602	30.0499	30.0923
s-oxide red	Q9HYL2	nosZ	33	54.1	70.659	323.31	5.49E+10	276	636	30.2723	30.6125	30.5711	30.0362	27.4002	27.0711	27.3491	27.0629
	Q9HYP7	PA3349	19	58.7	34.402	160.25	5.6E+09	121	310	27.8546	28.1195	28.2637	27.6787	27.0712	27.2951	27.0265	27.2754
	Q9HYP8	PA3347	5	56.4	11.226	57.386	7.97E+08	29	101	25.7961	25.0188	24.6852	25.7512	24.238	24.8169	24.3771	24.5207
manno-hep	Q9HYQ8	hldD	17	63	37.188	278.35	3.23E+09	77	330	27.1509	27.3105	27.6363	26.5089	24.7507	24.7991	23.3012	24.8984
	Q9HYT2	PA3312	7	47.6	30.907	128.37	1.24E+09	48	296	25.3474	24.8808	24.4925	25.3635	25.72	25.7451	25.5252	25.6122
	Q9HYT5	PA3309	15	77.5	16.496	320.25	2.18E+11	372	151	32.836	33.0357	32.9445	32.9361	30.8502	30.7969	30.843	30.6295
olyl cis-tran	Q9HYX8	PA3262	10	44.3	26.845	69.699	5.41E+09	80	253	26.7617	27.1308	26.2585	27.1529	28.0417	27.5165	27.7801	27.8729
	Q9HYZ0	PA3250	13	58.5	38.285	64.193	8.07E+08	45	352	25.2117	25.1332	25.6423	24.8103	23.9767	23.3539	23.3816	22.8923
family ami	Q9HYZ3	apeB	12	39.4	46.658	73.51	1.18E+09	53	429	24.8205	24.5751	23.2622	25.0118	25.8594	25.4761	25.4181	25.1833
in 2,3-diox	Q9HZ00	PA3240	6	47.4	31.627	46.43	7.76E+08	20	285	25.8815	25.9509	26.2531	26.1644	23.1491	23.5681	22.7413	23.5944
	Q9HZ15	PA3225	13	54	34.098	94.416	4.13E+09	89	309	26.8443	27.1438	27.3974	26.7221	26.2709	26.415	26.4629	26.322
	Q9HZ16	PA3224	3	38.1	11.43	70.455	7.22E+08	30	105	25.679	25.6909	25.3165	25.6979	24.8521	24.775	24.9339	24.5014
	Q9HZ19	csaA	7	66.7	12.091	57.468	2.08E+09	36	111	26.8176	27.0565	26.98	26.8541	25.9793	26.1358	26.1024	26.2135
	Q9HZ35	PA3205	3	15.8	16.696	38.97	1.97E+10	45	146	27.4455	27.3763	28.0464	27.674	31.809	30.8609	31.2916	31.0268
	Q9HZ48	PA3190	27	63.1	45.131	152.88	2.48E+10	185	420	30.0714	30.0222	30.1927	29.9282	29.0563	28.1312	28.3917	28.7393
	Q9HZ51	PA3187	11	42.2	42.24	90.762	1.03E+09	44	386	25.4493	25.8328	26.0101	24.7846	23.2495	24.0326	24.1158	24.565
	Q9HZ64	PA3170	5	20.9	48.493	36.602	7.36E+08	20	444	25.2707	25.0645	24.378	24.3303	25.7355	25.2167	26.1262	25.6328
drouridine	Q9HZ95	dusC	8	43.9	35.328	52.134	3.13E+08	41	319	23.697	23.4654	23.3034	23.7899	24.0965	24.1958	24.2517	24.3268
e dehydrat	Q9HZA3	leuC	11	37.1	51.042	63.493	1.58E+09	56	474	26.0286	26.0562	26.5144	25.6699	25.3674	25.5875	25.2183	25.5211
	Q9HZC8	PA3080	6	30.7	38.478	25.469	2.41E+08	12	365	24.8263	24.6415	23.7873	24.3947	22.6568	23.7138	22.8035	23.6127
lutamate d	Q9HZE0	gdhB	69	55.9	182.64	323.31	1.53E+10	326	1620	26.5899	27.1248	25.9686	27.0687	27.8746	27.4338	27.0394	27.6889
	Q9HZF4	PA3054	22	42.9	71.197	169.05	3.83E+09	88	634	26.751	26.9612	27.2328	26.6956	25.3474	25.1982	25.6559	25.2265
e modulati	Q9HZF9	rmf	5	55.7	8.1351	72.083	4.76E+09	36	70	28.6899	28.4937	28.1809	28.4349	24.2867	24.0077	23.7155	23.4247
3-phosphat	Q9HZK4	PA3001	16	47.7	50.111	323.31	6.13E+10	201	461	29.7013	29.8901	29.704	29.6497	30.5217	30.5401	30.485	30.5257
	Q9HZP1	PA2957	8	38.2	25.028	23.029	2.14E+09	45	212	26.5089	26.7347	26.4317	26.3846	27.3198	27.2318	26.9625	26.9274
	Q9HZT4	PA2913	3	15.5	35.366	14.949	1.64E+08	10	323	24.1319	24.213	24.7238	23.9483	22.6341	23.1439	22.5753	23.398
	Q9HZV1	PA2896	5	46.4	22.268	50.579	3E+08	21	194	22.7006	23.4075	24.3116	23.7734	24.3771	24.6624	24.572	24.3643
	Q9HZX1	PA2875	5	28.2	32.851	8.9836	2.17E+08	15	305	23.6472	23.5825	22.766	23.3254	24.2171	23.8841	24.1174	24.1155
rotease Htp	Q9I013	htpX	5	24.4	31.593	30.23	2.97E+08	14	291	23.2014	21.992	22.5782	22.4679	25.0968	25.0304	24.6009	25.3755
	Q9I022	PA2821	9	61.8	24.716	98.431	1.42E+09	60	220	25.232	25.2858	25.3841	25.3966	24.5564	24.7047	24.6423	24.7395
	Q9I036	PA2807	6	42.4	22.458	35.421	8.74E+08	23	205	23.8989	22.2396	21.9421	23.3358	26.2876	26.12	25.6353	26.0295
Ecotin	Q9I088	eco	9	51.3	17.228	135.23	1.21E+10	55	156	29.0209	29.27	28.8749	29.0973	29.8784	29.5293	30.0603	29.5957
	Q9I0C9	PA2713	5	44.7	17.808	18.787	1.93E+08	16	159	24.4717	24.3945	24.2367	24.3204	24.073	22.9166	22.6002	23.2479
	Q9I0D1	PA2711	6	34.2	40.111	19.916	2.79E+08	22	363	24.7042	24.5568	24.8215	24.4037	23.6484	22.6309	24.3577	24.0467
	Q9I0E8	PA2694	3	66.7	11.933	10.57	2.46E+08	11	108	22.3423	23.9896	23.3217	22.7031	24.5641	24.6092	24.7877	25.1416
	Q9I0F1	PA2691	11	42.6	42.607	129.86	1.42E+09	57	401	23.8416	24.2185	24.3031	23.4654	26.3141	25.754	26.1402	26.4693
	Q9I0F4	PA2684	15	19.4	145.82	51.968	6E+08	30	1317	22.937	24.0021	23.2	22.9561	24.9613	25.1451	25.1501	24.3354
ohemopro	Q9I0H4	hmp	17	66.9	43.658	221.9	1.61E+10	159	393	28.9499	28.8915	29.3175	28.7507	27.2724	26.8931	26.8643	27.4422
oxidoredu	Q9I0J4	nuoI	12	71.4	20.61	73.086	1.18E+09	51	182	24.3027	25.0366	24.9917	24.6338	25.0362	25.4164	25.6744	25.5095
dosuccinate	Q9I0K9	purB	19	59.6	50.5	190.91	1.71E+10	178	456	28.9006	28.9421	28.3285	29.3039	29.8545	29.6651	29.3315	29.1792
	Q9I0R1	PA2575	11	61.5	22.222	119.18	2.46E+10	126	200	29.427	29.4845	29.4831	29.1437	29.0815	28.7352	28.8368	28.4799
	Q9I0T0	PA2554	11	66.7	26.426	169.93	9.35E+09	102	255	27.9202	28.1579	28.8255	27.833	27.1568	26.8204	27.2502	26.7914
	Q9I0T1	PA2553	25	75	41.4	323.31	3.11E+10	240	396	28.768	28.7779	29.6294	28.4319	27.9496	27.5536	28.0535	27.6759
	Q9I0T2	PA2552	15	57.9	40.908	141.33	4.1E+09	102	375	26.905	26.9295	27.792	26.4826	25.9384	25.7524	26.2866	25.8823
	Q9I0T4	PA2550	7	30.8	45.482	36.834	3.5E+08	16	409	25.2234	25.0823	25.1335	25.0853	23.222	21.8519	23.5384	24.7921
	Q9I0V4	PA2529	16	53.5	52.681	116.59	1.88E+09	74	477	25.4312	25.5066	25.7932	25.1894	26.1441	25.9688	26.1265	26.0963
	Q9I0V5	PA2528	8	31.2	45.276	30.908	1.71E+08	15	426	23.2828	22.9309	23.2113	22.1586	24.0699	23.3665	23.3417	23.9382

	Q9I0W1	czcB	12	36.8	50.715	53.17	4.58E+08	20	484	22.4907	23.112	23.941	23.3139	25.5346	24.8915	24.2793	25.0436
	Q9I0X8	PA2504	4	26.8	22.822	16.418	3.6E+08	13	205	25.2655	25.6778	25.2194	25.3541	24.7396	23.0667	22.9579	23.0558
	Q9I101	PA2481	9	38.8	31.249	40.981	1E+09	39	291	26.226	25.6333	25.3441	26.1662	24.5838	24.8783	24.7605	24.8128
	Q9I103	PA2479	13	72.6	24.747	146.59	4.23E+09	59	226	24.1781	23.7147	23.986	23.5154	28.0624	28.1769	28.1306	27.9974
nterchange	Q9I104	dsbD2	5	14	62.093	24.972	4.75E+08	17	587	22.4986	22.7508	22.8471	24.3038	25.9388	25.5468	25.3049	25.8577
interchang	Q9I106	dsbG	16	70.7	28.053	245.91	4.64E+10	136	256	24.1765	23.897	22.9366	24.0345	30.9852	30.889	30.9478	30.8252
	Q9I120	PA2462	22	7.9	573.17	91.146	6.68E+09	35	5627	29.5927	30.0688	29.7366	29.9499	23.0756	28.9771	23.5019	23.0955
	Q9I171	PA2410	4	26.2	31.732	18.921	5.38E+08	18	305	23.549	22.8963	24.2996	23.0954	25.8341	25.9682	26.525	25.8783
	Q9I1C6	PA2352	21	72.5	41.13	157.86	8.66E+09	149	375	27.8817	28.0117	27.9679	28.0154	27.4859	27.1815	27.1015	27.2492
	Q9I1F7	PA2318	6	84.3	12.889	103.06	2.28E+10	108	121	30.2616	29.8897	28.976	29.9818	28.562	28.8963	28.8529	28.9813
	Q9I1K7	PA2266	14	48.7	46.74	87.758	1.67E+09	67	439	26.4411	26.315	26.0466	26.3949	25.4322	25.4009	25.3951	25.4498
	Q9I1K8	PA2265	16	36.9	64.715	69.568	2.29E+09	85	591	26.1435	26.2217	26.1164	26.0966	25.4234	25.1599	25.1796	25.2586
	Q9I1L0	PA2263	6	26.8	35.615	37.292	3.56E+08	25	328	24.2891	24.809	25.479	24.1584	23.4184	23.2333	24.131	23.5913
anched-cha	Q9I1M0	bkdB	20	58.2	45.774	190.61	4.25E+09	112	428	26.9233	27.3746	27.1115	27.1784	25.8595	25.4732	25.5123	25.6367
dehydroge	Q9I1M1	bkdA2	15	58.3	38.324	111.67	6.78E+09	100	350	28.0484	27.9253	28.6892	27.4346	25.848	26.0665	26.3433	26.2138
lehydroger	Q9I1M2	bkdA1	17	60	45.256	226.09	6.81E+09	89	410	28.046	27.8733	28.9325	27.3457	26.0342	25.7602	26.1962	26.4287
	Q9I1N7	pslB	9	32	53.515	35.166	4.76E+08	30	488	24.8537	25.4621	25.2679	24.4579	23.7508	24.3514	24.1941	24.4692
	Q9I1Y9	PA2126	10	63	23.677	62.791	1.19E+09	36	211	26.2458	26.7922	27.0329	26.5052	24.2466	24.3194	24.538	24.322
	Q9I1Z6	PA2119	16	66.9	38.561	222.98	1.76E+10	151	366	29.3304	29.3677	29.6578	29.0973	27.4181	27.2716	27.374	26.9772
	Q9I295	liuR	6	53.7	15.631	68.074	9.69E+08	31	134	25.7744	25.621	25.7911	25.3901	24.7663	24.9604	24.9939	25.0639
	Q9I296	liuA	23	72.6	42.209	200.19	8.07E+09	160	387	28.1615	27.9876	28.5097	27.5707	27.2637	27.0921	27.6727	27.2166
	Q9I298	liuC	4	23.4	28.947	29.054	7.28E+08	24	265	26.0864	25.5988	26.0831	25.9122	25.5207	24.7845	25.3952	25.3199
yl-CoA/hyd	Q9I2A0	liuE	8	35.7	31.837	34.702	4.79E+08	33	300	24.3441	24.459	24.7566	24.3189	23.9954	23.3245	23.9946	23.6981
	Q9I2A9	dhcB	6	40.8	23.187	38.841	1.11E+09	32	218	26.7656	26.1621	26.4369	25.8678	25.3784	25.236	25.6699	24.9129
	Q9I2B0	dhcA	7	51.3	25.243	41.458	1.53E+09	50	232	26.8516	26.5075	26.4389	25.8431	25.2391	24.851	25.4603	24.6081
	Q9I2F8	rbsB	6	28.5	33.86	16	4.38E+08	21	319	24.9257	25.2133	25.5649	24.9758	22.5341	22.29	23.8255	22.6323
	Q9I2N2	modA	6	32.3	26.377	28.242	1.26E+09	47	251	26.353	26.4662	26.616	26.4242	25.3381	25.302	25.2741	25.0026
	Q9I2P9	cti	16	30.4	86.517	68.806	5.92E+08	48	762	25.1784	24.8166	23.5804	25.4624	23.467	23.7747	23.312	23.5743
	Q9I2Q4	PA1841	5	37.6	18.654	15.412	4.47E+08	16	165	23.5024	25.0269	24.243	24.6007	25.4285	25.3464	25.1055	25.0123
	Q9I2T7	PA1807	4	13.8	59.074	26.018	1.41E+08	16	536	22.6152	23.1115	22.746	23.7646	24.1887	24.1164	24.2085	24.1204
ylglucosam	Q9I2V0	lpxH	3	22.5	27.554	14.839	2E+08	11	240	23.0898	23.3118	23.9942	23.6145	23.9257	24.172	24.1592	24.2874
	Q9I2V3	PA1789	19	76.7	31.273	293.01	2.54E+10	162	287	29.4952	29.7649	30.0377	29.2986	27.3936	27.3918	27.4358	27.1832
	Q9I2X1	PA1768	10	56.4	19.72	24.433	3.41E+09	39	179	27.6604	28.2158	27.8924	27.8479	27.0889	27.2551	27.3993	27.2602
	Q9I2X2	PA1767	9	28.1	56.812	83.114	7.35E+08	35	508	24.4268	24.691	23.7052	24.727	25.3673	25.3411	25.1073	25.439
	Q9I2Z1	PA1746	9	76.2	18.189	170.52	4.45E+10	142	168	31.9753	31.7242	31.6615	32.0006	29.2495	29.323	29.3899	29.321
	Q9I311	bglX	9	17.9	83.002	16.979	2.05E+08	20	764	24.2282	24.2804	24.67	23.9065	23.0966	23.4895	23.0691	22.7239
	Q9I321	exsD	8	35.1	31.365	31.478	1.25E+09	35	276	26.3228	26.4124	26.512	26.508	25.7972	25.5017	25.6297	25.7839
	Q9I323	popD	6	33.6	31.309	72.343	9.88E+08	20	295	26.2412	26.2918	26.4796	26.1577	23.0737	24.0123	24.2377	23.3362
	Q9I324	popB	6	29.5	40.061	55.474	1.52E+09	30	390	26.9486	26.2535	27.1765	26.5527	25.0098	22.1896	24.117	24.5696
	Q9I338	PA1689	12	28.7	77.984	68.226	1.9E+09	53	700	24.5656	24.8095	22.9164	24.9507	25.6691	26.2292	26.1846	26.3548
eriohemery	Q9I352	PA1673	10	52.9	17.853	167.92	1.38E+10	89	153	29.6705	29.7944	29.0998	29.8122	28.0515	28.0278	27.4538	27.4955
	Q9I371	PA1654	8	41.8	43.311	55.308	7.87E+08	33	388	24.7604	24.6254	24.6321	24.6535	25.0838	25.3857	25.5765	25.7125
	Q9I3D3	sucA	42	59	105.88	323.31	3.69E+10	380	943	28.3962	28.7707	27.9604	28.8741	29.3576	29.2786	28.8893	29.3155
	Q9I3F7	PA1560	4	37.3	15.667	35.892	7.6E+08	18	142	23.3268	24.2344	22.4476	22.1661	26.032	26.1133	25.9939	26.6663
	Q9I3F8	PA1559	12	71.2	26.551	93.168	1.67E+09	44	243	24.2576	23.9655	22.8709	24.5681	26.7915	26.4501	26.2134	27.0452
	Q9I3G1	ccoO2	7	37.1	22.778	26.711	1.94E+09	38	202	27.2533	27.3116	26.693	27.5404	23.4866	25.9691	23.8817	26.358
chrome c c	Q9I3G2	ccoP2	9	45.5	33.812	53.42	9.7E+08	41	308	25.323	26.1046	25.0428	26.0192	24.3684	23.966	23.8134	24.2142
	Q9I3G3	ccoN1	3	10.9	53.108	12.092	5.04E+08	23	475	24.5367	25.1502	24.0124	25.0907	26.0629	25.6101	24.9645	25.7517
protein Zi	Q9I3I5	zipA	9	53.6	32.236	99.766	1.36E+09	58	289	24.4315	24.9583	24.4803	24.8243	25.4335	25.1324	25.4288	25.4953
	Q9I3Q6	PA1441	5	23.4	44.18	23.312	3.01E+08	19	427	24.0623	23.0121	22.1044	22.9582	24.3947	24.6506	24.4605	24.777
nidinobuty	Q9I3S3	gbuA	11	43.6	34.727	75.716	4.2E+09	63	319	27.7834	28.1165	28.3921	28.2141	27.1923	27.3229	26.9046	27.1369
	Q9I402	PA1342	23	75.5	33.054	323.31	7.36E+10	275	302	30.6585	30.7228	30.6132	30.5969	29.8658	29.8636	29.9048	29.8102
nase-aspar	Q9I407	ansB	16	61.9	38.644	203.75	3.22E+10	195	362	29.1375	29.3461	29.7983	29.142	28.6228	28.6471	28.6507	28.5175
	Q9I448	PA1296	9	52.6	33.729	55.531	1.11E+09	37	310	22.1919	23.7642	24.1738	23.3407	26.3892	26.2027	26.7242	25.893
hione pero	Q9I457	PA1287	6	46.7	20.217	34.49	1.23E+09	31	184	26.7898	26.3455	25.7429	26.7877	25.5619	25.7864	25.2541	25.9128
	Q9I484	PA1260	7	39.9	29.142	34.693	3.89E+08	18	273	24.8551	24.5758	25.5418	24.6421	22.9149	23.8575	22.896	23.177
	Q9I4B0	PA1228	3	38.8	12.362	22.057	1.27E+09	24	116	25.929	26.3129	25.692	26.4434	26.9663	26.751	26.7666	26.3718
	Q9I4B3	PA1225	7	48.1	22.551	56.301	2.71E+08	21	208	24.5427	24.8542	23.8437	24.9344	24.3955	22.9458	22.2895	22.5657
	Q9I4G6	PA1171	10	37.9	42.682	61.409	1.6E+09	41	398	26.0401	26.4557	26.501	26.2718	25.5587	25.7179	25.6971	25.5334
	Q9I4I0	PA1157	3	22.5	26.405	17.076	2.7E+08	12	236	23.4376	22.7872	23.4217	22.4025	24.3487	24.7678	25.2624	23.0396
e-diphosph	Q9I4I1	nrdA	48	60.6	107.11	323.31	2.03E+10	337	963	27.0145	27.4913	26.577	27.5083	28.0822	27.7331	27.7689	28.1267
	Q9I4J8	PA1137	12	40.9	34.731	59.446	9.49E+08	42	328	26.1633	25.6714	26.4066	25.6007	24.5086	24.3147	24.6097	24.3818
rone Hsp31	Q9I4K0	hchA	5	26.5	31.898	30.229	5.5E+08	22	291	25.729	26.0067	26.2157	26.0115	25.1806	23.6008	25.4045	24.8575
	Q9I4L2	PA1123	7	86.8	12.167	57.915	1.08E+09	37	106	26.7629	26.3135	26.729	26.917	24.9066	24.9623	25.8562	24.9076
	Q9I4Q3	PA1076	5	52.5	13.743	64.311	1.43E+10	57	120	30.3958	29.7947	29.0953	30.3537	27.9672	28.8187	28.1718	28.8035
xamine 5'-	Q9I4S5	pdxH	12	60	24.86	163.17	1.36E+10	88	215	29.3945	29.4135	29.0343	29.6455	28.1962	27.944	27.7856	27.8785
	Q9I4W2	PA1011	11	53	43.055	79.29	1.46E+09	52	396	24.6761	25.1334	24.3402	25.3386	26.2914	25.7146	26.3447	25.9456
n-associate	Q9I4Z4	pal	7	61.9	17.925	166.74	6.35E+09	80	168	27.3725	27.3704	26.4847	27.6943	28.1239	28.0537	27.996	28.3379
	Q9I4Z7	PA0962	9	62.2	17.493	153.61	4.03E+10	114	156	30.9416	31.1889	31.0839	31.0064	30.0154	29.9713	30.0328	29.8472
	Q9I500	PA0959	9	46.4	23.069	120.7	3.16E+09	61	209	27.0276	27.1366	27.6063	27.0468	26.5955	26.7141	26.5463	26.3268
	Q9I524	relA	11	17.3	83.815	57.138	4.34E+08	26	747	23.9196	24.9627	23.7755	23.6175	25.54	24.7009	24.9177	25.5105
ne--tRNA li	Q9I553	alaS	40	57	94.701	323.31	1.56E+10	303	874	27.0099	27.22	26.7872	27.3609	27.7845	27.7121	27.5877	27.7894
nylpyruvat	Q9I576	hpd	16	52.7	39.912	175.96	1.99E+09	47	357	26.8465	26.4138	26.9988	25.028	23.7596	22.7674	22.4125	23.9624
76 protein	Q9I583	PA0858	12	51.6	35.42	65.193	1.34E+09	55	312	24.9206	24.6792	24.3103	24.824	25.6677	25.8945	25.6728	25.8645
hydratase	Q9I587	fumC2	19	64.7	49.121	248.99	1.32E+10	160	464	27.4241	27.2975	27.4115	27.1802	28.1711	28.2925	28.669	28.2059
	Q9I588	PA0853	8	50.2	23.485	66.509	2.64E+09	49	207	26.2677	26.2528	26.6902	26.4933	27.5928	27.192	27.9135	27.1998
edoxin redu	Q9I592	trxB2	8	59.8	33.481	107.67	2.12E+09	48	316	26.2074	26.1311	26.1542	26.1692	27.5082	27.3094	27.5641	27.2009
	Q9I593	PA0848	7	66.8	21.814	114.57	1.94E+09	47	199	25.9294	25.6852	25.9491	25.4923	26.631	26.7392	26.8504	26.4905
lyl cis-tran	Q9I5A3	slyD	5	85.7	17.01	151.93	1.72E+10	119	161	30.3474	30.8607	31.2593	30.1696	29.2537	29.9083	29.801	29.3124
cetate kina	Q9I5A4	ackA	17	65	42.421	211.05	1.47E+10	174	394	28.516	28.767	29.0356	28.6178	27.6501	27.3449	27.5324	27.2269
te acetyltra	Q9I5A5	pta	19	46.3	75.696	119.59	2.18E+09	75	704	25.5093	25.9605	25.5437	25.5681	24.6665	24.8843	24.9467	25.3262
	Q9I5A7	PA0833	8	57.8	24.713	91.853	2.06E+09	47	237	25.3564	25.501	24.1398	25.525	27.3694	26.7537	26.7588	27.2159
	Q9I5D3	PA0805	4	73.3	8.5276	39.077	5.45E+08	19	75	24.841	24.4659	23.3421	25.2298	25.4773	25.9882	25.3871	26.1221
	Q9I5H3	PA0756	3	24.2	24.431	3.854	2.33E+08	7	223	23.7601	21.7487	22.4387	23.0726	25.2379	22.7762	25.5728	25.3502
	Q9I5I8	PA0741	5	38.5	22.686	18.978	4.49E+08	27	213	24.9585	24.6763	24.2933	25.0041	23.6664	24.001	24.133	24.1831
nethoxy-1,	Q9I5R6	coq7	7	55.8	23.644	62.428	4.55E+09	58	215	27.213	27.2566	27.4008	27.1552	26.499	26.5141	26.6534	26.4144
	Q9I5T4	0222;PA06	14	52.9	38.167	103.88	8.92E+09	107	348	27.724	27.9653	28.209	27.7707	26.7786	26.8204	26.1122	27.1803
-transporti	Q9I5T5	PA0603	15	71.3	40.655	135.04	5.07E+09	121	369	27.0905	27.1488	27.457	26.7442	26.2003	26.1936	25.8034	26.985
	Q9I5T6	PA0602	8	41	37.833	37.314	3.75E+08	18	344	24.341	24.7154	24.9322	24.1918	23.9376	22.7335	22.471	23.4233
embly prot	Q9I5U2	lptD	6	12.1	104.27	33.999	4.03E+08	18	924	23.4853	24.36	22.8583	23.8349	24.3324	24.5484	24.3415	24.6856
	Q9I5U9	PA0588	24	47.3	73.722	130.92	3.6E+09	103	640	26.0394	26.8142	26.1462	26.2575	24.5757	23.5776	24.0335	24.6058
29 protein	Q9I5V0	PA0587	9	30.5	48.742	30.668	2.88E+08	20	423	24.5671	24.9197	24.4648	25.1371	22.4156	23.5539	23.4317	22.702
	Q9I5V1	PA0586	16	41.6	60.814	138.54	3.95E+09	66	517	27.6958	27.841	28.6465	27.2951	24.6171	24.5151	25.3085	23.451
	Q9I5Z9	PA0537	9	57.9	22.18	83.851	4.72E+08	23	202	22.7096	23.8788	23.3413	23.9301	24.4772	24.2194	24.8349	25.1987
	Q9I609	nirN	14	45	53.978	101.45	8.92E+08	31	493	25.2662	25.348	25.9326	25.1275	24.1466	23.4246	23.1719	23.446
	Q9I634	PA0484	2	29.2	18.553	26.229	2.43E+08	8	171	25.2094	25.0328	25.6459	23.7155	23.2276	22.0911	22.9422	23.2647
	Q9I659	PA0459	50	63.2	94.174	323.31	2.23E+10	269	850	28.9953	29.0854	29.5172	28.6943	27.0977	26.9904	27.5934	27.0767
	Q9I669	PA0449	8	62	19.839	98.683	5.29E+09	82	179	28.2557	28.6825	28.7229	28.0987	27.6868	27.7788	27.5929	27.4912
	Q9I671	gcdH	21	69.7	43.311	306.75	1.3E+10	127	393	28.8544	28.8182	29.2678	28.7553	26.6573	26.029	26.6531	26.4301
	Q9I672	PA0446	20	66.8	43.846	199.52	3.76E+09	122	407	26.7576	26.2501	27.1162	26.8093	25.3	24.8768	24.9892	25.1156
	Q9I680	codA	16	55.1	47.074	103.58	2.78E+09	85	423	26.2151	26.5563	26.0175	26.0542	26.8617	26.8923	26.7158	26.8509
L2 protein	Q9I690	PA0423	22	84.8	20.776	320.85	1.09E+11	274	191	30.4855	30.466	29.256	30.483	31.7663	31.2844	31.4324	31.1861
	Q9I6H9	PA0312	4	43.3	17.414	33.812	3.99E+08	21	164	25.3887	25.2906	25.3835	25.2012	23.2801	22.9972	24.5054	24.9093
ng periplas	Q9I6J0	spuE	21	74	40.069	190.01	3.19E+10	223	365	30.1347	30.0648	30.3094	30.0701	29.4448	29.4514	29.5052	29.4606
	Q9I6J6	PA0295	14	50.1	38.981	94.395	3.8E+09	85	353	27.7199	27.4535	26.7742	27.8612	25.7735	26.3133	25.6807	26.0767
	Q9I6J8	guB;PA350	19	70.9	32.759	283.79	3.07E+10	190	292	28.9333	28.8697	29.0896	28.8479	29.4879	29.5612	29.6579	29.544
atine deimi	Q9I6J9	aguA	15	55.7	41.191	306.2	2.43E+10	155	368	29.2232	29.2749	28.2616	29.1897	29.9081	30.0212	29.9492	29.9845
	Q9I6N0	PA0259	7	25	52.444	29.703	2.79E+08	16	480	24.4608	24.7259	25.0124	24.1014	22.3384	23.2188	22.6477	23.5565
	Q9I6R3	PA0225	3	25.1	19.993	29.323	1.43E+08	13	179	24.2832	23.7656	24.4888	24.393	24.1582	23.1938	21.4864	21.9154
	Q9I6T8	PA0200	4	64.3	7.7945	63.703	1.01E+09	22	70	27.8745	26.2951	25.1502	28.1116	22.6395	24.314	23.3824	22.9638
	Q9I6V3	PA0179	5	65.3	13.148	31.957	4.26E+08	16	121	25.0229	25.1945	25.6283	25.127	22.7286	23.2928	24.1853	23.2428
	Q9I6V6	aer2	24	53.8	72.59	167.87	1.38E+09	60	679	24.9808	25.333	25.9735	24.8698	23.3523	22.1523	23.0003	23.8104
	Q9I6Z1	PA0141	18	66.1	40.788	176.93	7.68E+09	118	357	27.5119	28.4778	27.7617	27.7733	26.2831	25.7995	26.1512	26.3876
oxide redu	Q9I6Z2	ahpF	22	52.8	55.835	185.61	8.75E+09	167	521	26.8235	26.8598	27.021	26.7698	27.7365	27.4416	27.5576	27.3348
	Q9I732	PA0100	7	31.7	33.723	49.337	8.82E+08	32	306	23.7094	25.5366	22.942	25.3995	26.2646	26.1769	26.5848	25.5309
	Q9I741	A0095;vgrG	7	19.6	72.012	56.97	3.18E+08	19	643	23.0503	24.6183	23.3054	23.7038	24.548	25.3894	25.3174	24.6043
rotein ClpV	Q9I742	clpV1	19	30.2	98.737	77.377	5.33E+08	30	902	23.08	23.3709	22.5391	24.1568	24.4237	24.5368	25.8697	24.3568
rotein hcp	Q9I747	hcp1	13	77.2	17.414	178.03	9.89E+09	112	162	27.5469	27.585	27.3669	27.6035	29.1416	29.2461	29.6513	28.4964
	Q9I748	PA0084	19	67.1	55.771	162.31	2.96E+09	93	498	23.9013	24.2232	23.159	23.982	26.348	26.2653	27.0348	26.4565
	Q9I749	PA0083	7	54.7	18.785	46.37	4.05E+09	40	172	26.1326	25.903	25.1428	26.5577	28.284	28.2999	28.4893	27.7662
	Q9I753	PA0079	12	45.9	48.274	51.7	5.02E+08	25	444	23.514	23.1456	24.0628	24.2654	24.622	24.5687	25.4278	24.7056
	Q9I773	osmC	4	47	15.61	29.819	4.97E+08	21	151	23.7428	23.393	23.1138	24.0609	25.4373	24.897	26.0983	25.4512
n regulator	Q9X6W6	pyrR	10	81.8	18.676	198.92	1.31E+10	89	170	28.8238	28.416	27.8504	28.6454	27.6408	27.7516	27.5039	27.7391
	G3XD10	nirJ	6	23.3	43.952	21.107	1.09E+08	10	387	23.0324	23.8774	23.871	24.058	22.3353	23.1513	23.1726	23.5332

	G3XD71	PA0620	2	6.2	72.292	8.7943	99903000	9	691	23.9443	23.1724	24.1046	24.2349	22.8476	23.152	24.1566	24.5852
›rotein ass	O68562	bamE	3	38.6	19.395	11.234	1.8E+08	12	176	23.3836	22.4799	24.0626	23.1813	22.0182	24.5603	24.0074	24.4109
ıntaining pı	P42810	PA4667	4	11.2	66.29	16.453	1.9E+08	12	590	23.7435	23.3562	21.9493	23.3001	23.905	22.8825	24.1107	23.8962
ᵓrotein Nirŀ	P95415	nirH	5	30.4	19.582	10.704	1.54E+08	11	171	23.9793	24.4736	24.2834	22.844	22.9079	24.1475	21.6921	22.9597
	Q9HT29	PA5545	3	16.3	33.627	14.163	1.38E+08	13	319	24.168	23.9832	24.0584	24.2337	22.5911	24.451	22.523	22.7838
	Q9HT84	PA5487	5	12.1	73.998	32.499	1.25E+08	9	671	23.1412	22.907	23.0499	24.2399	23.6155	23.3123	23.8582	23.5807
	Q9HUA0	PA5083	3	35.3	12.558	8.0832	92849000	11	116	24.3219	22.4803	22.923	23.3342	23.4585	22.5558	23.3955	24.1379
	Q9HUU0	PA4873	4	20	46.299	12.437	1.02E+08	17	421	24.3988	23.0892	24.4001	23.3679	24.4136	24.1945	23.443	24.3209
	Q9HUW7	PA4843	9	31	61.599	42.612	2.48E+08	13	542	24.1246	24.6103	24.2104	24.2719	22.9727	23.4995	24.3372	24.7239
	Q9HVQ6	PA4516	4	19.9	29.587	11.523	1.16E+08	11	271	23.501	24.1566	23.0246	23.1832	23.879	22.9053	24.4736	23.9676
	Q9HW12	PA4394	2	13.7	29.817	14.725	1.99E+08	13	278	23.3538	23.5774	23.6334	24.511	24.1115	24.0838	24.0563	24.4668
	Q9HX60	PA3956	8	97.9	15.453	44.921	7.88E+08	15	144	27.0345	23.3294	23.9155	26.6043	23.3884	24.397	23.2672	22.3217
	Q9HXS0	nalC	2	20.2	23.533	14.921	1.42E+08	8	213	23.0928	23.6149	22.8989	24.5709	24.3118	24.6448	23.1077	23.8055
	Q9HXW8	PA3672	2	7.8	33.499	6.3282	98465000	8	307	23.4421	23.1036	22.1023	22.3713	23.8367	23.0026	23.845	23.7831
	Q9HYI8	PA3417	6	30.7	40.414	22.437	2.42E+08	11	365	24.7621	22.6769	25.0435	24.4863	23.9803	22.466	23.2142	22.8264
	Q9HYI9	PA3416	2	11.4	36.143	15.821	1.18E+08	8	333	24.2478	23.6242	24.898	23.7743	22.3133	23.7863	23.2264	23.2025
	Q9HYJ0	PA3415	8	43.8	39.52	53.42	3.79E+08	16	370	24.1697	24.7907	25.724	23.9246	23.1673	22.2322	23.8652	23.9246
	Q9I0X9	PA2503	7	29	45.895	19.642	1.96E+08	13	417	23.528	23.2151	24.3309	23.0523	23.4406	24.1696	23.9214	23.8252
;tidine kina	Q9I102	PA2480	4	15.5	47.692	13.62	89224000	13	440	23.5845	22.7875	22.8826	23.0709	23.2017	22.7999	22.7503	23.2288
	Q9I124	PA2458	3	18.2	41.261	17.124	1.18E+08	12	373	22.4032	24.5829	24.53	24.3773	22.3288	22.6007	23.0236	23.558
	Q9I125	PA2457	2	8.9	36.102	11.77	63192000	7	326	23.9004	22.64	23.6874	23.7335	22.7085	22.9965	23.1599	22.8947
	Q9I2A6	bdhA	3	23	26.727	10.916	1.69E+08	11	256	24.0358	24.0812	24.347	23.8633	23.8712	22.9458	22.5005	23.7952
	Q9I2B2	PA1997	5	14.4	71.929	22.19	1.98E+08	11	651	23.2659	23.6937	24.3663	24.0237	23.3438	22.1843	22.9991	23.767
	Q9I2R3	PA1832	4	24.9	38.069	16.557	90460000	7	341	23.1738	23.221	22.4082	24.0265	24.4197	22.7283	23.7534	24.019
	Q9I4E5	PA1193	6	37.2	25.292	11.464	1.95E+08	14	223	23.2358	23.1737	23.4675	23.8009	24.2938	24.1453	23.0679	24.4847
ıic nitrate r	Q9I4G3	napA	8	14.5	92.974	16.184	1.52E+08	13	829	23.6871	23.7825	24.4678	23.5758	23.842	23.6159	24.1229	23.0353
	Q9I4X8	PA0991	3	32.8	13.286	14.686	1.66E+08	11	125	23.6261	23.6694	21.8314	24.2406	23.2372	23.2467	22.9562	23.6464
zyme inhib	Q9I574	mliC	3	37	13.695	9.1778	1.47E+08	13	127	23.7923	23.9375	23.4898	22.4457	24.266	24.0108	23.8714	24.2295
	Q9I673	PA0444	6	25.3	45.265	31.49	2.5E+08	18	427	24.927	24.7157	23.5246	24.6553	22.7871	23.3418	23.242	24.5654
	Q9I6W5	PA0167	2	12.2	24.533	7.0685	1.99E+08	12	221	23.1775	24.1683	23.8381	22.7863	24.7482	24.5886	23.3677	24.6565
hain releas	E1JGJ8	prfB	19	63.7	41.17	323.31	1.43E+10	176	364	27.9361	27.8251	28.0347	27.7251	27.9997	28.2035	28.3476	28.3124
/Cys-tRNA(	G3XCT4	PA3580	3	25.6	16.493	14.589	3.9E+08	15	156	25.4654	23.2886	23.3611	25.3132	25.239	25.4686	25.1506	25.4955
;tidine kina	G3XCT6	PA1458	24	50.3	79.737	291.08	1.14E+10	216	753	27.8241	27.6987	27.9216	27.6816	27.5128	27.6	27.5542	27.8555
	G3XCU6	cysB	11	47.2	36.109	115.06	3.11E+09	69	324	26.8287	27.0522	27.3016	26.5566	26.5997	26.7242	26.7549	26.6333
	G3XCU9	PA2020	3	16.7	23.727	6.2049	1.98E+08	13	210	23.8758	23.2683	24.0793	23.0481	24.1825	24.5225	23.1737	24.2598
	G3XCV0	fleQ	20	52.7	55.276	190.36	7.2E+09	120	490	27.5167	27.4882	27.8449	27.0377	27.1957	27.2481	27.3319	27.6062
	G3XCX8	PA0900	2	31.2	10.641	84.031	1.24E+09	20	96	27.038	26.5381	25.1162	26.4655	26.7411	26.9651	26.8856	26.6812
	G3XCZ3	pilZ	6	66.9	12.904	25.183	1.6E+09	41	118	26.1709	26.0573	25.4096	26.5909	25.9605	26.3026	25.7733	25.9569
carrier prc	G3XCZ6	fabD	16	56.1	32.444	261.1	2.97E+10	158	312	30.2119	29.9901	29.711	29.9851	29.837	30.0924	29.7635	29.9321
	G3XD09	gltD	7	27.9	52.624	62.423	9.51E+08	42	477	25.1736	25.0445	25.2215	25.1134	25.3292	25.6778	25.7864	25.2762
	G3XD11	oprH	7	44.5	21.574	130.87	2.26E+09	39	200	26.3424	26.3771	26.2555	26.4407	26.7675	26.146	26.8116	26.5644
	G3XD13	PA0902	11	55.7	35.462	118.17	2.34E+09	72	334	26.2988	26.5132	26.4495	26.2246	26.4049	26.5735	26.1579	26.2961
ıding trans	G3XD14	dksA	12	73.6	17.344	129.96	5.48E+10	182	148	30.6173	30.5716	29.1893	30.7827	31.0477	30.9611	30.8665	30.9178
	G3XD17	dsbC	8	57	26.095	153.33	8.31E+09	74	242	28.037	28.1333	27.6019	28.232	28.6806	28.4458	28.6533	28.5122
ıg chemota	G3XD24	ᵓ;PA4915;p	12	30	68.016	36.405	1.25E+08	12	629	24.1733	24.3713	23.0133	23.7894	23.1186	23.0799	24.2486	23.4002
	G3XD27	PA5055	7	81.3	13.653	55.029	1.74E+09	40	123	26.7474	26.3005	25.111	26.6647	26.1458	26.6968	25.8728	26.2219
	G3XD36	ptsP	15	36.6	83.606	81.35	1.12E+09	60	759	25.134	25.0301	24.9974	24.917	24.7977	24.6031	24.9953	25.2787
	G3XD47	aotJ	23	81.1	28.01	273.55	4.54E+10	212	259	29.4673	29.684	29.1103	29.2139	29.3783	29.7768	30.0765	29.7544
	G3XD52	PA3302	8	78.2	16.901	84.965	7.09E+09	102	156	28.4571	28.058	28.1445	28.4649	28.1809	28.0045	28.2517	28.3307
	G3XD54	dnaQ	10	54.1	26.814	132.99	5.46E+09	97	246	26.9891	26.9553	26.7938	27.0164	27.2709	27.2331	27.2005	27.1977
	G3XD55	pilY2	3	30.4	12.986	11.776	2.77E+08	11	115	22.6199	22.8323	23.0688	25.1424	23.5643	25.0405	24.9552	25.1195
amate met	G3XD63	chpB	5	34.7	37.237	24.755	5.21E+08	18	343	23.3867	25.8004	25.6565	26.3151	20.7874	25.6261	22.8453	25.547
termining		G3XD64	fleN	10	41.1	30.037	71.401	2.74E+09	69	280	26.1004	26.1032	26.5579	25.8439	25.6312	25.7506	25.8433	25.7574
	G3XD70	PA5279	4	33	25.767	68.789	3.93E+09	41	233	27.5816	27.7142	27.6054	27.7667	27.6815	27.5296	27.5627	27.5163
	G3XD72	PA1464	11	83.6	17.536	169.8	1.66E+10	102	159	28.8174	28.7533	28.6972	28.8264	27.8877	28.4935	28.5668	28.5038
	G3XD74	dacC	11	45.6	42.457	126.37	5.83E+09	92	386	27.163	27.782	27.31	27.6079	27.8517	27.5694	27.8008	27.9684
!,6-dicarbo	G3XD76	dapD	12	43.6	35.973	116.97	1.74E+10	115	344	28.7223	28.6541	28.9196	28.6708	28.7293	28.6528	28.5597	28.5091
	G3XD95	chpC	9	71.4	17.625	137.62	1.75E+09	71	168	26.1561	25.859	24.9312	26.0387	25.1984	25.6468	24.6616	25.5011
	G3XD97	ptxS	14	56.8	37.391	111.39	2.12E+09	47	340	27.9941	27.253	27.6497	27.1426	27.3271	23.2165	25.5157	27.7621
carrier-pro	G3XDA2	fabF1	13	62.1	43.497	323.31	2.94E+10	142	414	29.4288	29.4111	29.5327	29.2632	29.5505	29.5503	29.4318	29.469
	G3XDA4	PA0653	10	83.6	15.236	81.348	4.57E+09	77	140	27.3038	27.4587	27.3566	27.4667	27.2773	27.3283	27.177	27.1366
	G3XDA5	oprE	5	18.7	49.666	28.826	9.36E+08	30	460	26.3655	26.7082	25.8542	26.6596	25.8715	25.9009	25.9232	25.9422
	G3XDB0	crc	13	70.7	29.844	98.78	5.06E+09	101	259	27.2611	26.9506	27.2689	27.1166	26.8795	27.1358	27.1584	26.9898
	G3XDB2	PA0974	15	61.3	29.097	168.53	1.69E+10	150	274	28.7895	27.9668	26.9022	28.8497	28.6986	29.2425	28.4677	29.2596
ıe phospho	O05927	cysH	8	41.9	30.215	86.202	2.38E+09	52	267	26.424	26.7114	26.8389	26.3134	26.2854	26.2204	26.4078	26.2857
nsport ATP	O30506	aotP	8	41.7	27.956	15.026	1.53E+09	41	254	26.668	26.743	26.8019	26.4161	26.6773	26.5599	26.5357	26.774
ıase/acetyl	O30508	aruC	28	81.8	43.747	323.31	1.06E+11	421	406	30.4175	30.6166	30.8605	30.3218	30.2826	30.3698	30.5064	30.317
ıcyl-carrier-	O33877	fabA	11	53.8	18.747	119.65	1.42E+10	95	171	29.5692	29.4707	29.4975	29.4308	29.9858	29.4619	29.5171	29.3311
5-semialde	O50174	astD	19	50.7	51.379	249.46	2.39E+10	163	487	28.6115	28.7861	29.111	28.632	28.9466	28.7441	28.6216	28.6059
larginine di	O50175	astB	20	59.6	48.874	239.36	1.49E+10	157	448	28.8584	28.9139	29.1583	28.6862	28.7313	28.7668	28.8281	28.9266
tamate de:	O50177	astE	10	37.7	36.908	76.544	1.51E+09	38	332	25.9321	26.1686	26.4147	25.9737	25.8413	25.8365	26.1059	25.8524
⁄lyltransfer	O50273	cysD	12	42.3	35.478	39.552	2.41E+09	62	305	25.9895	26.3161	26.1714	25.9133	26.1789	26.0813	25.871	26.0518
me CysN/C	O50274	cysNC	16	37	69.268	98.606	2.03E+09	83	633	25.067	25.2381	24.8301	24.9024	25.4094	25.4021	25.35	25.6982
osomal prc	O52759	rpsD	14	48.5	23.277	107.99	4.1E+10	163	206	29.6169	30.1075	29.3425	30.1067	30.3364	30.2577	30.0975	30.1991
A polymera	O52760	rpoA	27	70.3	36.649	323.31	1.79E+11	409	333	31.2761	31.4802	31.6249	31.2834	31.3845	31.3985	31.4066	31.347
›somal pro	O52761	rplQ	9	62	14.504	47.504	5.63E+10	105	129	30.9405	31.3296	30.5935	31.341	31.5895	31.488	31.409	31.6014
Catalase	O52762	katA	27	70.7	55.588	221.12	8.57E+09	171	482	27.6321	27.8308	26.6096	27.4194	27.1101	27.1012	26.7354	27.034
rrier-protei	O54438	fabG	11	75.3	25.585	274.95	6.48E+10	143	247	30.441	30.6154	30.7938	30.5539	30.2321	30.3678	30.2544	30.2966
carrier prot	O54439	acpP1	4	43.6	8.7406	17.445	1.71E+11	106	78	33.8022	33.1474	32.3061	33.9179	33.3526	33.8775	32.8613	33.5902
scriptional	O68281	hexR	5	20.4	31.169	29.144	3.01E+08	26	285	23.6707	23.3664	24.0099	23.507	22.8732	23.779	23.8	24.1534
›sphate 1-d	O68282	zwf	8	22.5	55.62	18.601	2.11E+08	19	489	23.3695	23.4168	22.9859	23.8939	23.3412	23.2406	23.7128	24.2005
y-phospho	O68283	eda	6	36.4	23.953	20.378	9.16E+08	35	220	25.7035	25.2862	25.4052	25.3867	25.9339	25.434	25.6025	25.1896
association	O68560	ratA	6	54.2	16	38.293	1.17E+09	42	144	26.1904	25.8416	26.1802	26.3922	26.0251	26.1919	25.7662	26.0794
l aminopep	O68822	pepA	21	57.8	52.331	168.45	5.28E+09	130	495	27.1622	27.2679	26.6659	26.8891	27.2454	27.1131	27.1827	27.6665
nerase III s	O68823	holC	5	63.4	16.141	21.844	1.12E+09	23	142	26.5643	26.3105	24.2903	26.721	23.2134	26.5207	25.8647	26.7385
spartokina:	O69077	lysC	25	70.4	44.388	323.31	2.96E+10	220	412	28.9906	29.0371	29.1451	28.8976	29.3556	29.3976	29.3863	29.2822
age regulat	O69078	csrA	3	52.5	6.9089	100.38	9.16E+09	62	61	29.6331	29.6643	29.7872	29.6016	29.5619	29.2027	29.8252	29.3405
osomal prc	O82850	rpsB	18	69.1	27.336	323.31	1.3E+11	465	246	31.4493	31.6969	31.9343	31.5987	31.5987	31.5084	31.6085	31.5719
gation fact	O82851	tsf	35	81.3	30.653	323.31	4.12E+11	954	289	32.5788	32.5383	32.8261	32.7093	32.7319	32.626	32.7132	32.6178
idylate kina	O82852	pyrH	14	72.7	26.272	272.36	1.62E+10	132	245	28.5454	28.5298	28.7313	28.516	28.2957	28.5778	28.5675	28.4449
ne-recyclin	O82853	frr	16	74.6	20.485	226.39	6.58E+10	180	185	31.0842	31.2511	30.7207	31.1441	31.1314	31.2223	31.2652	31.139
RNA formy	O85732	fmt	11	57.3	33.028	102.18	7.3E+09	82	314	27.4367	27.6857	27.1681	27.642	27.3955	27.3266	27.1874	27.2133
pyrophosp	O86062	nudC	6	27	31.355	36.807	8.71E+08	35	278	25.684	25.3577	25.1114	25.8775	25.9111	25.8557	25.5476	25.6867
mino-acid	O86428	ilvE	16	56.4	34.084	323.31	2.29E+10	128	307	28.8759	28.5244	27.8628	28.693	29.0961	29.5978	28.7104	29.326
protein ho	O87014	usg	15	62.5	35.349	116.65	2.31E+09	86	336	25.6657	25.5182	25.6126	25.6349	25.6057	25.819	25.8065	26.0434
douridine :	O87016	truA	12	68.4	31.483	73.098	2.5E+09	81	285	25.7966	25.948	26.1555	25.7573	25.762	25.8089	25.4149	25.6046
:in-glutama	O87125	cheB1	13	68.5	39.004	178.16	7.33E+09	96	368	27.6353	27.7537	28.058	27.4381	27.0358	27.3928	27.3012	27.4672
otein meth	O87131	cheR1	5	40.9	30.647	17.741	2.29E+08	15	274	24.3647	24.3008	23.8701	23.2641	23.7884	24.1899	23.7772	22.1577
⁄tochrome	P00106	cc4	3	28.4	20.734	33.169	1.95E+09	26	201	27.156	26.2593	25.5041	26.8968	27.0898	27.4845	26.2854	28.0801
Azurin	P00282	azu	7	60.1	16.008	127.45	7.9E+10	143	148	32.5492	31.3634	29.6166	32.1337	29.8425	31.4648	29.559	30.8147
ling proteir	P05384	hupB	5	53.3	9.0865	56.143	4.87E+11	439	90	34.707	34.8634	35.2019	34.8175	34.8393	34.7551	34.772	34.4966
ı synthase	P07344	trpA	7	48.1	28.488	64.186	2.55E+09	54	268	26.3062	26.144	26.5458	26.3404	26.1317	26.159	26.5253	26.155
n synthase	P07345	trpB	15	60.7	43.68	108.62	3.6E+09	92	402	26.5081	26.5178	26.3157	26.2418	26.2143	26.5585	26.2946	26.2726
ᵓrotein Rec	P08280	recA	19	78	36.879	206.54	3.3E+10	213	346	29.314	29.2609	29.4511	29.1376	29.0235	29.1571	29.2479	29.2668
gation fact	P09591	tufA	34	90.2	43.369	323.31	1.77E+12	2189	397	34.2696	34.2334	34.5061	34.2589	34.3422	34.3637	34.4029	34.2301
interchang	P0C2B2	dsbA	5	27	23.375	22.706	8.4E+08	21	211	25.2885	25.2379	24.896	25.1851	25.5047	25.2617	25.1989	25.3114
	P0DP44		12	27.7	78.292	33.862	5.28E+08	23	690	24.2084	24.5118	23.0444	24.6325	25.2656	24.6113	24.5717	25.3928
carbamoylt	P11724	argF	10	56.1	33.924	89.344	2.29E+09	68	305	26.6183	26.4284	26.6363	26.8297	26.3699	26.6544	26.4973	26.4632
l regulator	P15275	algQ	5	41.9	18.011	20.493	2.68E+09	24	160	28.1901	28.0964	26.5719	28.2288	27.2548	28.0539	27.136	27.7002
ıl regulator	P15276	algP	4	15.3	34.49	17.126	1.85E+09	21	352	27.3002	27.4687	27.0371	27.6633	22.3844	26.9458	27.2498	27.5252
melate dec	P19572	lysA	21	67.7	45.49	238	1.09E+10	173	415	27.3262	27.3731	27.2947	27.055	27.5146	27.5507	27.4741	27.3935
hosphoribc	P20574	trpD	13	67	37.383	224.32	4.1E+09	93	349	26.057	26.0908	26.4313	26.1034	26.0553	26.1565	26.4034	26.3722
synthase c	P20576	trpG	8	69.2	22.046	179.6	5.31E+09	58	201	27.509	27.3023	27.9367	27.4862	27.6022	27.654	28.0824	27.5893
erol phospl	P20577	trpC	15	47.5	30.347	81.722	4.13E+09	68	278	26.7448	26.8008	27.0075	26.7899	26.8751	26.9629	26.8876	26.7769
ino acid tra	P21629	braF	9	52.5	28.283	79.002	9.33E+08	39	255	25.0198	25.2676	25.5339	24.8727	24.844	24.887	24.6236	24.7906
-carboxylat	P22008	proC	8	53.5	28.094	50.672	1.48E+09	62	273	26.3119	25.8093	25.8708	26.2127	26.4079	26.3563	26.56	26.3402
:id acetyltra	P22567	argA	7	30.8	47.858	43.442	2.28E+08	25	432	23.6189	23.009	23.4975	23.0306	22.8682	23.1641	23.9661	24.4616
al assembl	P22608	pilB	10	30	62.327	137.7	1.8E+09	67	566	25.7659	26.1853	25.9745	25.7189	25.5487	25.5207	25.4232	25.5719
thione redu	P23189	gor	22	68.3	49.237	219.36	9.96E+09	164	451	27.4911	27.5199	27.2794	27.3268	27.4868	27.3784	27.6088	27.287
scriptional	P23620	phoB	9	62.4	25.711	52.435	7.3E+08	32	229	25.1687	25.5419	25.7878	25.0819	24.671	22.7568	24.8217	24.8226
ıg mobility	P24559	pilT	21	71.5	38.02	168.43	8.98E+09	157	344	27.027	27.2497	27.2602	26.982	26.5799	26.6819	26.5923	26.6589
)1 protein I	P24562	PA0394	9	62.2	24.505	38.678	1.9E+09	30	230	26.879	26.7252	23.4775	26.9209	26.9586	26.9391	26.3035	26.8043
osynthesis	P26275	algR	12	59.3	27.608	97.078	5.32E+09	63	248	27.1881	27.2677	27.3836	27.1628	27.4877	27.3255	27.7829	27.3363
rase sigma	P26480	rpoD	28	56.4	69.643	323.31	2.63E+10	242	617	28.848	28.8768	28.6975	28.9121	28.8541	29.0559	28.9359	29.1388

3-phosphat	P27726	gap	8	39.8	36.17	131.66	2.38E+09	60	334	26.3	26.4803	26.6894	26.1031	26.5953	25.9152	26.5169	26.1749
butyrate de	P28811	mmsB	4	26.8	30.486	23.562	2.91E+08	19	298	25.4738	24.6272	23.2904	25.0516	22.4925	23.2347	24.194	23.4892
onine synt	P29363	thrC	24	62.7	51.795	196.45	8.75E+09	147	469	27.2285	27.4463	26.7603	27.43	27.7561	27.7628	27.7563	27.9462
oserine ki	P29364	thrB	8	31	35.374	53.833	1.64E+09	53	316	26.6471	26.6316	25.9126	26.7581	26.3782	26.6918	26.6154	26.4625
rine dehydi	P29365	hom	13	48.2	46.223	194.8	2.09E+10	140	434	28.747	28.7891	28.862	28.411	28.7576	28.7063	28.9203	28.9519
Da chaper	P30718	groL	49	78.2	57.085	323.31	1.11E+12	2194	547	33.9094	33.897	34.2853	33.9213	34.1123	33.9289	34.0901	33.8527
Da chaper	P30720	groS	5	51.5	10.267	158.36	1.23E+11	130	97	32.8403	32.8567	31.8623	33.0513	32.8378	32.7505	32.5501	32.9423
yrophosph	P30819	nadC	11	44.7	30.551	147.04	6.81E+09	110	282	26.9345	27.069	27.1173	26.9891	26.7222	26.9325	26.8539	26.7806
uconate de	P31961	edd	18	49.7	65.182	245.49	8.6E+09	136	608	26.9913	27.0368	27.1324	26.7787	27.4022	27.181	27.5513	27.212
scriptional	P32265	nfxB	5	44.9	21.077	66.227	9.76E+09	42	187	28.8633	29.0118	28.9101	29.0729	29.3509	29.4205	29.3831	29.057
unit pseud	P33640	rluD	6	22.5	35.625	27.109	6.4E+08	23	320	25.0074	25.0517	25.1994	25.0899	25.2752	24.9982	24.7919	25.0696
nino acid o	P33642	PA4548	8	41.8	39.445	34.993	1.24E+09	44	364	25.479	25.163	25.7155	25.1252	25.7349	25.6535	25.5852	25.8212
main prote	P33663	PA4543	5	43.4	26.047	40.285	4.97E+08	23	242	25.402	22.7704	23.8031	25.4543	24.8806	26.0583	22.3054	25.5323
roquinate	P34002	aroB	15	54.6	40.109	119.9	1.03E+10	134	368	27.2862	27.2297	27.3795	27.3306	27.2568	27.2047	27.2812	27.1548
kimate kin	P34003	aroK	5	40.1	19.237	40.375	4.1E+08	14	172	24.7367	24.9018	25.253	24.8054	24.7934	24.8879	23.2266	25.2885
etion syster	P35818	xcpQ	6	24.6	69.952	12.565	1.67E+08	20	658	23.7931	23.5156	23.4796	23.7242	23.9215	24.4484	23.8444	22.9444
exA repress	P37452	lexA	5	35.3	22.506	33.332	2.59E+09	39	204	26.6656	27.0824	27.2845	26.9596	26.9214	26.875	26.7456	26.5123
in carboxy	P37798	accC	22	56.3	48.887	301.02	5.93E+10	230	449	29.7835	29.957	29.9999	29.5339	29.7159	29.9137	29.9414	29.9083
protein of	P37799	accB	7	57.7	16.455	255.93	5.67E+10	166	156	30.9322	30.6934	29.5659	31.1882	31.0952	31.3965	30.6121	31.236
phate synt	P38098	carA	16	62.7	40.828	225.78	2.49E+10	169	378	29.2697	29.3682	29.2289	28.8599	29.0599	29.3103	29.1696	29.2766
phate synt	P38100	carB	53	65.4	117.33	323.31	1.91E+10	345	1073	27.2845	27.6802	26.8943	27.4924	27.6374	27.6046	27.472	27.9075
hydrodipic	P38103	dapB	14	68.3	28.324	153.17	8.45E+09	89	268	27.679	27.9036	27.7877	27.6965	28.0082	27.9052	27.8838	28.0844
ed DNA-bi	P40947	ssb	12	67.9	18.557	143.73	3.14E+10	122	165	30.0761	30.305	29.7118	29.8987	30.3538	30.2785	30.4571	30.1701
Protein PilJ	P42257	pilJ	10	24.8	72.527	144.61	1.05E+09	46	682	25.1925	25.7823	24.8191	25.3431	24.9457	24.6114	24.524	25.0303
2-C-methy	P42805	ispE	3	21.3	30.843	13.305	3.86E+08	20	282	24.4953	24.6515	24.4664	24.5321	24.8709	24.4965	24.25	24.8683
hain releas	P42806	prfA	17	51.9	40.04	271.81	1.39E+10	102	360	29.8843	29.8963	30.158	30.0045	30.0345	30.0562	30.0342	30.0387
yl-tRNA re	P42807	hemA	7	30.8	46.123	80.432	8.09E+08	46	422	25.1704	24.9352	24.998	24.7535	25.1376	25.3185	25.1257	25.5699
no-acid am	P43336	phhC	13	50.4	43.273	72.182	2.42E+09	88	399	25.926	25.7841	25.773	26.0933	26.0708	25.7866	25.8661	25.851
Protein PilH	P43501	pilH	5	60.3	13.273	28.633	8.98E+09	40	121	29.7576	28.8207	27.7519	29.9762	28.221	29.0897	27.5583	28.9023
Protein PilI	P43502	pilI	5	46.1	19.934	42.613	5.13E+08	30	178	24.8137	24.7481	24.8665	24.7348	24.5292	24.6008	24.1248	24.4728
coproporph	P43898	hemF	15	63.6	34.806	119.95	1.11E+10	169	305	27.7637	27.9297	27.8289	27.9407	27.4251	27.4385	27.4152	27.2405
ne oxidore	P43903	qor	12	56.6	35.094	125.41	3.47E+09	89	325	26.5392	26.5414	26.5932	26.2386	26.1601	26.0199	26.0509	25.9779
artate O-m	P45683	pcm	9	56.9	23.407	103.06	4.97E+09	72	211	27.2521	27.4899	27.5424	27.3455	27.5594	27.5754	27.5272	27.6053
Protein PilG	P46384	pilG	7	59.3	14.718	49.757	7.71E+09	63	135	28.503	28.2226	27.4073	28.6754	27.1022	27.8001	26.8708	27.777
rision prote	P47203	ftsA	26	74.3	44.646	323.31	2.74E+10	245	417	28.6588	28.8428	28.9273	28.5127	28.7582	28.8723	29.0518	28.8625
vision prote	P47204	ftsZ	28	87.3	41.218	323.31	5.29E+10	281	394	29.6467	29.8914	29.9445	29.6951	29.3393	29.2502	29.3875	29.1781
oyl] N-acet	P47205	lpxC	17	54.8	33.435	160.56	4.01E+10	165	303	29.4679	29.7516	29.8098	29.4354	29.2915	29.4353	29.3734	29.4407
yrinogen-II	P48246	hemD	5	42.6	27.279	31.611	4.33E+08	28	251	25.11	25.0954	24.5975	24.8821	24.7884	24.6101	24.9901	24.9323
ialdehyde	P48247	hemL	24	74.5	45.397	316.96	2.09E+10	224	427	28.8353	28.9236	29.0236	28.4326	28.5235	28.7098	28.8593	28.9701
gyrase subu	P48372	gyrA	33	48.1	101.13	301.01	1.05E+10	208	923	26.7093	27.3125	26.5266	27.4113	27.465	27.1653	27.1735	27.5986
erase sigm	P49988	rpoN	16	46.3	56.054	153.96	3.95E+09	91	497	26.3895	26.4654	26.6812	26.1065	26.2713	26.4136	26.3453	26.2646
sphoribosy	P50587	pyrE	12	62.4	23.32	144.49	1.15E+10	112	213	28.2837	28.5314	28.6053	28.4572	28.5175	28.3358	28.1515	28.0873
onuclease	P50597	rph	5	34.7	25.654	97.391	3.19E+09	44	239	27.8991	27.9897	27.9909	28.1215	27.3831	27.6121	27.672	27.4635
Protein TolI	P50601	tolB	29	75.7	47.752	302.81	3.21E+10	239	432	28.5544	28.8549	28.975	28.5925	29.0387	28.9936	29.2456	28.8946
nosuccinat	P50987	argH	17	41.2	51.64	150.89	8.08E+09	120	464	27.0488	27.0375	26.84	27.0116	27.2465	27.3503	27.1678	27.3124
istance ope	P52003	mexR	4	38.1	16.964	14.821	3.51E+08	17	147	24.821	24.2237	23.2972	24.7166	24.085	25.0579	24.3875	24.8923
erase III su	P52024	holB	5	25.9	35.746	35.435	2.35E+08	18	328	22.6922	24.0534	23.7723	22.4309	23.8644	24.0924	24.2575	24.3657
osphate de	P52111	glpD	29	74.6	57.134	311.43	1.48E+10	218	512	27.9371	28.1015	28.0672	28.1111	28.1864	27.7911	27.9673	27.6615
e [ADP-forr	P53593	sucC	24	64.4	41.542	323.31	2.76E+11	578	388	32.0321	32.1183	32.2942	31.8641	31.9316	32.0947	32.1651	31.8273
xide dismut	P53641	sodB	10	66.3	21.351	135.96	3.56E+10	155	193	31.0503	30.1255	28.8081	30.8314	30.7924	30.9928	29.3704	30.6771
omoserine	P55218	metZ	17	52.9	43.184	150.2	4.59E+09	91	403	26.8703	27.1382	26.8959	27.1478	27.2172	27.4049	27.1682	27.0001
receptor-l	P55222	vfr	9	41.6	24.225	83.065	3.43E+09	65	214	26.5999	27.1609	26.5125	26.7419	26.531	26.6017	26.2286	26.5122
ucleotide	P57112	sthA	27	71.6	51.16	323.31	4.04E+10	297	464	28.6563	28.8003	28.5731	28.5256	29.1376	29.0259	29.0357	28.8405
goribonucle	P57665	orn	10	41.7	20.826	69.64	3.34E+09	89	180	26.8874	26.8006	26.4761	26.8376	26.4852	26.4122	26.4102	26.6157
le thiol per	P57668	tpx	14	91.5	17.234	289.98	4.1E+10	163	165	29.6357	29.4479	29.0694	29.7033	29.7011	29.6249	29.756	29.5345
l 4-phosph	P57707	ispD	6	41.5	25.613	67.942	1.07E+09	45	234	25.4297	25.3592	25.3293	25.513	25.5033	25.5134	25.7005	25.7722
e O-acetyl	P57714	metX	8	38.3	41.834	87.004	1.37E+09	37	379	25.1319	25.6302	25.1646	25.3094	25.7633	25.5881	25.5024	25.9779
n initiation	P65116	infA	4	69.4	8.3025	114.05	2.99E+10	80	72	30.5963	30.7656	29.5596	30.7727	30.9645	30.931	30.6957	30.7782
type flagell	P72151	fliC	2	8.2	49.242	85.116	6.63E+10	56	488	32.1246	32.2569	32.1697	32.4051	31.8739	31.7392	31.6461	31.7386
douridine	P72154	truB	13	57.2	33.41	193.33	8.61E+09	120	304	27.7963	27.8804	27.8995	27.6695	27.4782	27.7457	27.5364	27.6631
idazole rib	P72157	purE	8	62.6	16.889	95.159	1.68E+10	104	163	28.6283	28.2238	27.4658	28.5285	28.5161	28.9047	28.739	28.9452
idazole rib	P72158	purK	7	33.9	38.495	66.937	9.6E+09	84	360	27.8726	27.6622	27.6522	27.9935	28.1488	27.9299	27.8551	27.8208
hydroorota	P72170	pyrC	21	66.1	38.406	215.34	1.38E+10	168	348	28.085	27.68	26.7119	28.1111	27.813	27.9663	27.3284	27.9734
e aminotra	P72173	aspC	21	73.9	43.319	256.91	1.47E+10	153	398	27.5956	27.8477	27.4978	27.5607	27.8423	27.8506	27.7698	28.254
C system pr	P72174	uvrB	23	51.2	76.066	169.92	2.43E+09	94	670	26.3486	26.3342	26.6266	25.8403	26.0322	26.1795	26.287	26.5258
nyltransfer	P80357	astA	11	42.9	36.93	139.34	8.52E+09	85	338	27.9358	27.8808	28.1378	27.6746	28.1595	28.2822	28.1163	27.9498
nyltransfer	P80358	aruG	21	73.2	37.245	199.11	1.17E+10	143	340	28.0473	28.1337	28.2684	27.8281	28.3891	28.4345	28.0628	28.1457
-binding pr	P95453	PA4753	3	43.3	11.657	14.932	1.23E+09	23	104	26.3109	25.9579	25.668	25.8859	23.5275	25.8859	26.3444	26.0352
e subunit r	P95454	rlmE	6	44	23.499	52.739	1.93E+09	42	207	27.032	27.2379	27.2259	26.9944	26.5145	26.7982	26.9315	26.7094
rinogen de	P95458	hemE	10	45.9	38.792	109.89	5.01E+09	104	355	26.8221	26.7832	26.8955	26.6495	26.7959	27.2106	27.0972	27.3296
d shock pro	P95459	cspA	3	55.1	7.6054	40.343	9.19E+10	63	69	33.2083	32.8875	31.8012	33.5945	33.1452	33.474	32.9216	33.3574
protein Ra	P96963	radA	14	57.2	48.41	98.024	7.15E+08	47	453	23.205	24.7322	24.6351	24.245	24.2088	24.9318	24.7342	25.0916
ression reg	Q00934	pilR	19	53	49.736	141.16	2.09E+09	91	445	25.6021	25.435	26.0112	25.3763	24.9943	25.3804	25.3613	25.44
dienyl deh	Q01269	pheC	9	53	30.447	51.918	1.33E+09	43	268	25.9545	26.1376	25.7793	25.899	25.8562	25.4319	25.4279	25.3803
ke regulati	Q03456	fur	6	71.6	15.234	87.669	1.97E+10	73	134	29.2902	29.3755	29.5911	29.3824	29.5213	29.4435	29.7263	29.2531
osphoribos	Q04633	apt	7	57.1	19.804	44.111	1.1E+09	37	182	25.6243	25.6045	25.9638	25.8697	25.6038	25.6364	25.7399	25.5319
nerase sigm	Q06198	algU	11	71	22.196	205.4	8.71E+09	99	193	27.5202	27.8376	27.4773	27.5861	28.1047	28.0912	28.1215	27.826
scriptional	Q06553	prtR	5	41.8	28.112	19.046	1.14E+09	23	256	25.9846	26.3404	26.2908	26.4949	26.055	26.32	25.8409	26.0187
2 immunit	Q06579	imm2	9	67.8	10.039	55.038	8.14E+09	92	87	28.7646	28.7556	26.7618	29.0897	27.7142	28.632	27.5191	28.6221
phoribosyl	Q51342	purF	26	71.1	55.369	258.26	1.33E+10	178	501	28.1649	28.4669	28.3134	28.1066	28.2415	28.4949	28.5083	28.6079
ialdehyde	Q51344	asd	21	70	40.494	223.36	7.15E+09	124	370	27.0123	27.2616	27.0066	27.0619	27.0732	27.4489	27.2861	27.3913
nose 4,6-de	Q51366	gmd	11	39.9	36.399	45.851	1.6E+09	43	323	26.2648	26.5589	26.666	26.0378	26.5224	26.0594	26.3028	26.0988
se regulat	Q51373	gacA	9	53.3	23.608	50.621	1.43E+09	38	214	25.3234	25.7042	25.9353	25.4045	25.3302	25.2115	25.2518	25.231
malate deh	Q51375	leuB	10	46.9	39.12	108.67	2.77E+09	65	360	27.1382	27.4079	27.3086	27.0642	26.6585	26.9693	27.038	26.8688
protein Hs	Q51382	hscA	25	56.2	66.446	206.65	7.58E+09	153	619	26.8137	26.6975	26.8965	26.7538	27.0134	27.0357	27.0194	27.0792
-2S ferred	Q51383	fdx	9	89.3	12.241	75.869	1.58E+10	81	112	30.2442	29.3378	28.0562	30.3165	29.3923	30.2791	29.231	30.1838
erized prot	Q51384	PA3808	6	87.9	7.8017	47.575	2.2E+09	32	66	27.2062	25.9175	25.4701	27.427	26.4416	27.4332	26.4026	27.525
cerol kinas	Q51390	glpK2	22	52.7	55.966	177.55	5.89E+09	150	505	26.3842	26.5761	26.6928	26.5989	26.6483	26.5052	26.6599	26.3927
sphate reg	Q51391	glpR	8	51	27.568	64.74	9.58E+08	59	251	25.088	25.0011	25.1762	25.2734	24.9522	25.0435	24.7916	24.8822
tRNA(Asp/	Q51422	aspS	30	62.1	66.207	323.31	2.47E+10	239	591	28.8489	29.0254	28.9232	28.6329	29.0265	29.2345	29.2016	29.5676
regulator	Q51423	pmpR	2	16.1	26.556	65.287	1.26E+09	26	248	26.8049	26.132	26.2894	26.8334	25.9495	26.4036	26.5346	26.4405
-depende	Q51425	ruvA	6	45.8	21.959	42.767	2.07E+09	30	201	26.8185	26.7251	26.4071	26.8995	26.9874	27.302	27.1561	27.1811
-depende	Q51426	ruvB	15	68.2	38.932	119.48	2.96E+09	110	352	25.4914	25.6401	25.7049	25.4348	25.6517	25.5667	25.8201	25.6754
phosphata	Q51434	cheZ	15	80.6	25.709	185.77	8E+09	95	227	27.6512	27.9623	28.0016	27.8066	27.5134	27.4862	27.426	27.4583
taxis prote	Q51455	cheY	6	40.3	13.902	42.666	5.27E+09	50	124	28.0618	28.2023	28.1051	28.2041	27.4339	27.7365	27.8012	27.7938
tor switch	Q51464	fliG	15	54.7	37.007	96.149	3.78E+09	93	338	27.127	27.2425	27.5818	27.4478	27.1801	27.1679	26.9896	27.0202
tor switch	Q51465	fliM	15	58.5	36.281	74.933	3.32E+09	87	323	26.6847	26.6331	26.7671	26.4625	26.3408	26.5483	26.6165	26.3819
tor switch	Q51466	fliN	4	39.5	16.62	30.834	2.37E+09	20	157	27.5795	27.2845	26.9252	27.3842	27.6236	27.5039	27.5778	27.2878
)-dimethyla	Q51470	miaB	15	48.2	49.977	92.55	2.17E+09	73	446	26.4857	26.4686	26.4856	26.2113	26.8525	26.9632	27.0403	27.0479
ost factor	Q51473	ihfB	2	20.2	10.632	62.085	5.51E+08	14	94	26.2381	26.4522	22.8638	26.0869	24.804	23.3787	23.4463	22.6943
rt ATP-bin	Q51546	pstB	13	64.6	31.044	72.677	9.9E+08	59	277	24.6376	25.0408	25.0095	24.268	24.9251	24.9831	24.6817	24.8522
ystem acc	Q51547	phoU	10	47.1	27.487	150.81	1.6E+09	62	242	25.4654	25.6267	25.766	25.3986	25.5414	25.6608	25.7917	25.5261
protase-lik	Q51551	pyrC'	10	38.1	44.15	117.97	6.02E+09	94	423	27.5073	27.6965	27.7734	27.2646	27.1311	27.1807	27.2996	27.2868
IA polymer	Q51561	rpoB	81	66.5	150.84	323.31	7.9E+10	658	1357	28.6911	29.1993	28.2552	29.0732	29.4819	29.3	29.0955	29.7446
pimelate e	Q51564	dapF	14	72.8	30.319	212.47	6.14E+09	93	276	27.367	27.7485	27.6768	27.4583	27.3512	27.1342	27.2886	27.207
e [ADP-forr	Q51567	sucD	23	87.8	30.266	305.49	3.38E+11	452	295	32.4479	32.5254	32.5365	32.5702	32.3593	32.4196	32.4599	32.2552
d oxidored	Q51576	PA3106	8	43.1	27.029	49.001	1.97E+09	49	255	26.6082	26.7153	26.9154	26.4743	26.702	26.5751	26.681	26.602
e diphosph	Q59636	ndk	9	75.5	15.592	86.659	8.5E+10	197	143	31.9619	32.1772	31.0344	32.0515	31.9219	32.2983	32.3296	31.9953
drogenase	Q59637	aceE	50	59.6	99.563	323.31	2.89E+10	343	882	27.6795	28.4289	27.3646	28.1624	28.2107	27.6995	27.5346	28.3238
ase compo	Q59638	aceF	30	58.9	56.708	323.31	6.45E+10	304	547	29.6827	30.1061	29.5139	30.0135	29.9061	30.0053	29.8159	30.0637
yl cis-trans	Q59641	ppiA	9	62.6	20.104	261.2	2.74E+10	123	187	29.6247	29.6607	29.304	29.7805	29.637	29.447	29.549	29.3968
evulinic aci	Q59643	hemB	9	33.8	37.037	91.087	2.23E+10	108	337	29.3376	29.6282	29.6833	29.3429	29.6479	29.6042	29.4291	29.5545
l-D-glutam	Q59650	murE	21	60.6	51.263	140.78	3.55E+09	107	487	26.3877	26.6091	26.532	26.3984	26.6374	26.6281	26.7579	26.7646
carbamoylt	Q59653	pyrB	20	71.3	36.629	264.95	8.43E+09	131	334	26.9885	27.2115	27.568	26.8666	26.4156	26.7079	26.7037	26.6656
hosphate	Q59654	pyrF	16	89.7	24.394	181.02	1.56E+10	158	232	28.1617	28.0472	27.8964	28.1859	28.3934	28.2562	28.0929	28.1102
ilinogen de	Q60169	hemC	14	57.5	33.641	161.59	1.02E+10	120	313	27.9005	27.4058	26.9152	28.0314	27.5978	27.6514	27.129	27.5669
protein ins	Q9HT06	yidC	6	20.4	64.074	61.945	7.69E+08	30	578	24.342	24.7841	22.4728	24.6861	25.2845	25.0615	24.9736	25.4252
fication GT	Q9HT07	mnmE	13	47.5	48.744	107.68	1.38E+09	66	455	25.9712	25.861	25.9947	25.6454	25.6078	25.6976	25.7194	25.7732
minometh	Q9HT09	mnmG	15	37.3	69.637	111.87	2.47E+09	82	630	26.0421	26.0704	26.1289	25.9045	26.1198	26.614	26.3782	26.5904
ll subunit r	Q9HT10	rsmG	7	40.2	23.735	56.679	2.68E+09	31	214	26.9289	27.0656	27.3113	26.8109	26.6465	26.745	26.7786	27.0095
	Q9HT11	soj	5	30.5	28.609	55.956	1.26E+09	38	262	26.1907	25.943	26.2546	26.049	26.1195	26.3395	26.4874	26.4452
	Q9HT12	spoOJ	14	57.9	31.968	142.9	9.39E+09	98	290	27.6718	27.7015	27.9201	27.5306	27.4733	27.5134	27.634	27.604
nthase sub	Q9HT14	atpB	4	21.8	31.92	38.295	1E+09	25	289	25.4834	26.0327	23.0797	25.9357	26.5447	26.0294	25.3618	26.0303
nthase sub	Q9HT16	atpF	9	48.7	16.956	70.705	4.16E+09	61	156	26.9753	27.6131	26.8604	27.3559	27.8887	27.4822	27.1818	27.8633

thase subu	Q9HT17	atpH	14	78.7	19.26	296.89	7.38E+10	279	178	31.1266	30.7251	30.1797	30.9828	30.8262	31.0266	30.9044	31.1082
thase subu	Q9HT18	atpA	33	63.2	55.393	323.31	3.8E+11	549	514	32.2536	32.3482	32.5052	32.2975	32.5686	32.4404	32.3443	32.2292
thase gamr	Q9HT19	atpG	18	75.2	31.553	204.63	4.8E+10	192	286	29.7714	29.6566	29.7396	29.6419	29.8371	29.9054	29.5767	29.7256
thase subu	Q9HT20	atpD	42	93.4	49.499	323.31	5.04E+11	1207	458	32.1375	32.1866	32.4035	31.9725	32.2967	32.2543	32.254	32.2163
thase epsilo	Q9HT21	atpC	6	49.6	14.726	47.804	2.04E+10	62	141	29.9932	29.5624	29.4187	30.0279	29.9341	30.0196	29.8418	29.9441
N-acetyltra	Q9HT22	glmU	21	54	48.851	236.84	1.32E+10	173	454	28.1571	28.4297	28.549	27.9909	28.3424	28.4235	28.519	28.5379
	Q9HT24	glmR	6	45.9	28.151	16.416	2.74E+08	16	257	24.5808	24.204	23.1354	24.1901	24.4986	24.13	24.7524	24.2213
sphate ami	Q9HT25	glmS	19	44.8	66.294	151.51	2.93E+09	106	611	25.7446	26.0733	25.6343	26.112	26.2324	26.3105	26.2957	26.6103
	Q9HT52	PA5521	9	55.2	26.894	102.62	4.83E+09	73	252	28.2107	28.2953	28.3273	28.0291	27.9366	28.1843	28.2819	28.2563
	Q9HT54	PA5519	3	23.4	21.215	7.2815	3.52E+08	14	188	25.5799	25.5822	23.4511	25.2401	24.4557	24.5501	24.4323	24.2227
oxamine ki	Q9HT57	pdxY	9	46.5	31.285	42.013	2.51E+09	71	288	26.5335	25.9631	24.9869	26.53	26.2187	26.2643	25.7182	26.168
	Q9HT62	mifR	17	49.2	49.629	60.286	1.04E+09	54	447	25.5474	25.3996	25.4032	25.2613	25.3463	25.4376	25.7667	25.534
	Q9HT64	PA5509	10	59	24.841	67.174	2.7E+09	65	222	26.6761	26.7528	26.2943	26.7858	26.1591	26.5651	26.2166	26.3056
	Q9HT65	PA5508	9	44	47.9	79.352	1.62E+09	60	443	25.8994	25.9395	25.6068	25.5927	25.5435	25.9775	25.947	26.1048
	Q9HT66	PA5507	14	73.7	24.262	249.42	2.75E+10	169	217	29.7803	29.7678	29.9442	29.7571	29.2557	29.3126	29.523	29.3035
	Q9HT67	PA5506	10	42.5	32.143	102.97	1.26E+09	38	285	26.234	26.1002	26.5002	26.3047	25.8348	26.0975	26.1879	25.8634
	Q9HT68	PA5505	12	55	28.065	89.906	3.12E+09	64	260	26.7432	26.8157	22.4624	26.4164	26.6683	27.4553	26.9913	27.1719
t ATP-bindi	Q9HT70	metN2	11	65.4	36.759	95.41	2.44E+09	54	335	26.4108	26.7341	26.6902	26.1322	26.2825	26.6525	26.5936	26.702
	Q9HT74	np20	4	49.1	18.216	47.763	1.39E+09	25	167	26.3981	26.7329	26.3729	26.3889	26.3872	26.4044	26.2318	26.2054
A polymera	Q9HT80	polA	47	66.3	99.792	323.31	1.32E+10	303	913	26.9243	27.0243	27.064	26.669	26.8017	27.0523	27.0393	27.0955
P-binding p	Q9HT81	engB	9	57.7	23.786	55.469	2.3E+09	56	215	26.4199	26.6561	26.638	26.3955	26.3414	26.5394	26.5498	26.2937
	Q9HT98	PA5472	12	66.5	29.148	85.572	6.63E+09	101	266	28.5093	28.1881	27.3568	28.5083	27.3204	27.9172	27.0698	27.6174
	Q9HTC5	PA5441	23	50.2	80.036	177.94	4.57E+09	137	733	26.348	26.2322	26.27	26.067	26.2591	26.4235	26.4841	26.1959
	Q9HTC8	PA5438	10	51.5	31.68	53.73	1.94E+09	44	293	26.3913	26.7433	26.6044	25.9537	26.4836	26.5086	26.3851	26.6807
	Q9HTD0	PA5436	21	55.6	51.848	144.49	2.93E+09	121	471	25.9198	26.1001	25.9386	25.9655	25.8017	25.6046	25.5151	25.7449
ate ammon	Q9HTD7	aspA	19	66.9	51.069	289.56	4.96E+10	254	474	30.4845	30.2969	30.6316	30.2202	30.1475	30.679	30.5131	30.8153
	Q9HTD8	PA5428	8	43	33.477	54.079	7.45E+08	45	302	24.8457	25.0438	24.806	25.1108	25.3718	25.2199	25.1047	24.6869
	Q9HTE1	PA5423	3	28.6	20.002	16.028	6.55E+08	22	182	25.3608	25.5705	24.894	25.5379	25.0141	25.542	25.0018	24.0945
	Q9HTE2	PA5422	12	56.9	36.139	64.409	1.52E+09	54	318	25.2322	25.2292	25.5164	24.9251	24.9802	25.2459	25.6066	25.2222
	Q9HTF0	PA5414	4	31.5	22.547	13.927	2.22E+08	15	213	24.1005	24.1723	23.7623	23.9498	24.0559	23.8864	23.8679	24.2854
ty L-threor	Q9HTF1	ltaE	13	52.6	38.213	200.86	1.56E+10	119	346	29.1398	28.9947	28.934	28.9835	29.0562	29.0753	29.0471	28.9917
ision prote	Q9HTF7	PA5407	2	32.3	10.292	24.985	4.73E+08	13	96	25.578	25.3192	23.4621	25.6103	25.0812	25.2892	25.3481	25.3962
	Q9HTF8	PA5406	2	31.8	12.445	47.35	2.85E+09	27	110	28.4184	25.9046	25.3522	28.2008	25.7026	28.309	25.7537	27.7063
	Q9HTI8	PA5376	10	36	43.721	30.957	9.64E+08	44	392	25.0393	25.415	25.2245	24.9777	25.0841	24.8745	25.1679	25.0461
scriptional	Q9HTJ0	betI	5	32.5	21.861	25.854	4.24E+08	15	197	24.5652	24.2246	24.6951	24.687	24.1801	24.5664	24.606	24.5109
betaine ald	Q9HTJ1	betB	16	57.3	53.332	172.78	7.34E+09	114	490	27.5678	27.4798	27.4947	27.316	27.0678	27.5722	27.894	27.4495
ent choline	Q9HTJ2	betA	20	51.7	61.836	115.72	1.52E+09	96	561	25.6123	25.407	25.4939	25.7312	25.3958	25.673	25.9629	25.5498
	Q9HTJ3	PA5371	5	56.7	14.555	78.361	1.5E+09	45	134	26.7459	26.3216	26.7425	26.6847	26.7891	27.0754	26.8753	26.4243
	Q9HTJ6	PA5364	12	47.7	32.401	91.821	1.74E+09	55	300	26.0565	25.9566	26.1874	25.6665	25.5551	25.4141	25.7162	25.601
in-NAD(+)	Q9HTK9	alkT	15	67.2	40.617	180.69	4.67E+09	110	384	26.891	27.1422	26.9146	26.8081	27.0727	27.1808	27.2907	27.2243
	Q9HTL4	oxyR	18	75.2	34.295	159.4	7.34E+09	119	310	27.5217	27.7776	27.8056	27.2644	27.1539	27.3483	27.5398	27.4579
	Q9HTL5	PA5343	9	46.3	30.778	82.856	3.07E+09	74	283	26.6385	26.6955	26.848	26.6079	26.2448	26.5372	26.5953	26.4465
	Q9HTL9	PA5339	9	93.7	13.609	86.13	7.25E+10	211	126	31.0343	30.6899	29.9581	31.244	30.6614	31.4259	30.9285	31.0712
A polymera	Q9HTM1	rpoZ	7	44.3	9.7748	120.1	3.67E+10	84	88	31.2695	30.6933	29.4476	31.4038	30.8714	31.454	30.6659	31.3258
anylate kin	Q9HTM2	gmk	10	59.6	23.102	115.05	9.08E+09	90	203	28.3281	28.4603	28.2486	28.5273	28.3562	28.3051	27.8994	28.0341
	Q9HTM3	PA5335	12	39.7	31.708	69.613	3.99E+09	49	287	27.6353	27.6741	28.0162	27.515	27.4812	27.8128	27.7767	27.8552
	Q9HTM5	PA5330	8	50.7	22.434	100.12	2.37E+09	48	205	27.3104	26.976	24.9325	27.3274	27.4014	27.3798	26.6495	27.1422
glutamate	Q9HTN2	argB	19	83.7	31.849	231.79	2.7E+10	195	301	28.8978	28.9063	29.0277	29.0033	29.1962	29.2301	29.417	29.1292
hosphate r	Q9HTN3	dut	4	56.3	15.92	74.429	3.02E+09	29	151	27.7815	27.2934	26.6701	27.9792	27.0288	27.5497	26.947	27.3942
	Q9HTN4	coaC	17	72.9	43.133	235.34	8.2E+09	118	402	28.0752	27.9426	28.2783	27.861	27.8377	27.7568	27.7283	27.6621
osomal prot	Q9HTN8	rpmB	5	35.9	9.0655	18.539	1.69E+10	28	78	30.4977	31.2329	30.278	30.5695	31.2434	31.057	30.8367	30.718
osomal prot	Q9HTN9	rpmG	3	35.3	6.045	61.759	7.05E+10	58	51	32.2364	32.8605	32.8177	31.7571	32.6726	32.6413	32.8667	32.6408
	Q9HTP1	PA5313	5	20.7	48.347	13.075	2.71E+08	15	444	22.9366	24.0365	22.8445	23.7952	24.5796	24.3133	24.4329	22.3614
	Q9HTP2	PA5312	29	71	53.143	323.31	6.3E+10	352	497	30.476	30.6466	30.9335	30.1439	30.2352	30.21	30.2902	30.2312
	Q9HTP5	PA5309	12	45.1	47.701	81.241	2.4E+09	78	439	26.8946	27.1381	27.3103	26.9544	26.8206	26.9509	26.9342	26.7685
	Q9HTP6	lrp	6	46.3	18.868	14.455	7.19E+08	25	162	25.0981	25.5516	25.2777	25.0407	25.1135	24.9331	24.9343	24.8845
	Q9HTP9	PA5305	4	56.1	11.527	69.895	2.18E+09	35	114	28.0072	25.1341	25.3821	27.6325	24.4817	27.1892	24.4967	26.3214
	Q9HTQ1	PA5303	4	47	13.019	27.919	3.15E+08	20	117	25.2149	24.7445	23.1526	25.2479	24.1741	22.2826	23.8404	24.204
racemase,	Q9HTQ2	dadX	12	43.1	38.914	108.76	5.46E+09	59	357	27.5006	27.6367	27.4717	27.6471	27.5785	27.4558	27.431	27.1706
	Q9HTQ3	PA5301	10	67	19.962	79.777	5.01E+09	97	182	26.9461	27.2195	27.1749	26.9271	26.9839	27.0358	26.9238	26.8733
	Q9HTQ4	cycB	9	58.1	13.458	77.491	1.52E+10	83	136	29.9998	27.9568	27.4377	29.9478	28.9435	30.1712	28.3442	29.6538
osphoribos	Q9HTQ6	xpt	7	36.8	20.607	40.98	4.84E+09	56	190	28.0463	28.4623	28.6061	28.2499	27.9429	28.0363	28.1021	27.9815
	Q9HTR5	PA5289	5	66.3	9.5329	35.643	1.42E+09	30	86	26.7214	26.1877	25.5321	26.9113	25.9068	26.6046	25.6633	26.4515
	Q9HTR6	glnK	11	76.8	12.286	108.3	3.31E+10	122	112	30.0238	30.1334	29.5724	30.2454	29.778	29.6832	29.9063	29.6666
	Q9HTR9	PA5285	7	81	11.168	115.39	2.27E+10	77	105	30.0318	29.4038	27.9109	30.3001	29.8318	30.1388	28.4855	30.0864
Protein Cya	Q9HTS5	cyaY	5	58.3	11.942	69.036	2.05E+09	30	108	26.8609	26.6298	25.5324	27.0405	26.628	26.5992	26.1752	26.8105
	Q9HTS6	rnk	6	68.7	14.405	100.77	1.04E+10	96	134	28.829	28.3008	27.7044	28.9746	28.4742	28.7477	28.3066	28.6828
	Q9HTT1	PA5269	4	51.6	10.097	40.688	1.17E+09	25	91	26.9139	25.9339	23.1009	26.9889	25.7189	26.6772	23.2926	26.0411
	Q9HTT7	PA5258	13	46.3	40.791	143.97	4.84E+09	78	376	26.3748	26.5566	26.3364	26.6755	26.9606	26.894	27.1549	26.8989
	Q9HTU9	PA5245	12	80.6	23.425	246.93	2.98E+10	132	222	29.8747	29.847	29.2671	29.9556	29.2332	29.5024	29.2758	29.4469
terminatic	Q9HTV1	rho	39	79	47.069	323.31	6.45E+10	417	419	29.5688	29.6812	29.8491	29.4374	29.5439	29.6323	29.8207	29.6549
droxybenzo	Q9HTV3	ubiD	10	31.8	54.62	77.802	1.88E+09	61	488	25.8094	25.8781	25.7438	25.9532	25.9699	26.1439	26.2243	26.2307
	Q9HTV4	PA5236	8	43.8	35.42	53.919	1.24E+09	54	322	25.6096	25.5167	25.2144	25.5235	25.5486	25.4495	25.1421	25.4274
	Q9HTV6	PA5234	8	42.8	34.011	62.557	7.25E+08	46	325	25.14	25.2955	25.2443	24.7754	24.2919	24.6398	24.747	24.4543
	Q9HTV7	PA5233	4	51.8	15.218	86.502	2.01E+09	38	139	26.8622	25.5848	25.4825	26.9638	24.8343	26.4946	25.578	26.3016
	Q9HTV8	PA5232	10	40.1	38.609	29.88	2.67E+08	14	357	24.4838	24.5236	23.3244	25.2451	24.0446	23.8403	22.802	22.7012
	Q9HTW1	PA5229	5	48	16.871	16.303	1.34E+09	45	152	25.9939	25.4579	25.145	26.0414	25.8497	26.1574	25.7763	25.9994
ision prote	Q9HTW3	zapA	7	56.7	11.655	177.15	5.85E+09	62	104	28.1109	28.4698	27.9805	28.2546	28.1277	28.3667	28.968	28.2625
49 protein	Q9HTW5	PA5225	7	75	19.358	89.144	8.54E+09	65	184	29.2201	28.3654	28.3153	29.2639	28.8222	29.0319	28.8189	29.0762
	Q9HTW6	pepP	16	49.1	49.71	73.091	3.9E+09	101	444	26.9274	26.9214	27.2794	27.0278	26.7131	26.6851	26.8735	26.954
	Q9HTW9	PA5221	8	33.3	44.64	42.884	7.03E+08	33	405	24.3397	24.3746	25.0038	24.1565	24.6326	24.767	24.7562	24.8835
methyltran	Q9HTX5	gcvT	13	54.2	38.907	156.56	7.83E+09	112	360	27.5437	27.6712	27.8361	27.5006	27.3388	27.5132	27.512	27.401
vage systen	Q9HTX6	gcvH2	5	51.2	13.642	17.829	1.13E+10	59	129	30.0375	29.3268	28.6287	30.4005	29.3607	29.8066	28.893	29.8543
	Q9HTY0	PA5210	12	35.7	65.981	30.151	4.15E+08	27	594	24.6689	24.3871	24.2433	24.4558	24.2716	23.1184	24.2184	23.9991
	Q9HTY1	PA5209	13	36.8	51.989	76.237	1.78E+09	67	454	25.4935	25.5356	25.3771	25.3384	25.5502	25.7045	25.7497	25.7944
nithine dea	Q9HTY4	argE	7	35.9	42.163	34.247	3.54E+08	24	384	24.5968	24.4155	24.5759	23.0833	24.1044	24.4419	23.8915	24.4538
ate--cystein	Q9HTY6	gshA	21	53.3	59.223	136.04	4.22E+09	119	527	26.8933	26.8201	26.8765	27.2018	27.0362	26.9463	27.0472	26.9675
	Q9HTY7	PA5202	4	55.8	13.814	27.438	1.47E+09	29	129	26.2271	25.9033	25.9518	26.2962	26.428	26.7303	26.871	26.5695
	Q9HTY8	PA5201	43	60.7	84.963	323.31	2.31E+10	362	779	27.5184	27.6156	27.4866	27.3634	27.8813	28.0333	28.049	28.019
	Q9HTY9	amgR	15	66	27.924	132.71	1.14E+10	136	247	28.5402	28.6231	28.8652	28.6046	29.4422	29.1145	29.4523	28.9218
eptide carb	Q9HTZ1	PA5198	12	62.2	33.288	91.21	3.37E+09	82	307	26.46	26.0222	25.3632	26.3518	26.454	26.2293	26.4428	26.1745
pha-L-gluta	Q9HTZ2	rimK	6	34.9	32.604	27.315	3.42E+08	21	301	25.1847	24.4578	25.0183	24.4456	23.2275	23.8491	24.795	24.2073
Da chaper	Q9HTZ6	hslO	12	60.3	32.834	122.13	7.65E+09	74	297	27.7962	27.7435	27.6264	27.7506	27.7525	27.8186	28.2007	28.0923
ruvate carb	Q9HTZ7	pckA	33	67.8	55.731	323.31	3.6E+10	256	513	29.0962	29.1711	29.0592	28.8261	29.2796	29.3776	29.4406	29.4633
	Q9HTZ9	PA5190	10	61	22.449	145.38	8.02E+09	84	200	28.3857	28.2695	28.1733	28.3356	28.2816	28.1928	28.1549	28.143
onal chorisr	Q9HU05	aroQ	5	36.2	20.417	42.665	1.27E+09	31	185	26.3278	26.1202	22.9508	26.4421	25.7477	26.2907	25.0409	25.6219
	Q9HU13	PA5176	5	37.2	20.983	36.63	2.05E+09	36	188	26.2232	26.3037	26.6442	26.1452	26.4487	26.188	26.5298	26.1567
	Q9HU14	cysQ	13	65.6	29.779	46.756	9.7E+08	43	273	25.2987	25.2812	25.277	25.22	25.2258	25.1526	25.349	25.5111
	Q9HU15	PA5174	28	56.8	68.763	323.31	3.19E+10	227	634	29.5885	29.6889	29.5959	29.3364	29.7112	29.7528	29.5845	29.6279
	Q9HU18	0884;PA51	22	69.2	37.05	208.73	1.17E+10	193	331	27.9439	28.0842	28.5474	28.0459	27.9107	27.7377	27.9988	27.7627
	Q9HU19	PA5166	16	55.4	50.864	160.47	3.53E+09	79	462	26.3066	26.3058	26.5385	26.058	26.1515	26.1938	26.3725	26.2989
orhamnose	Q9HU21	rmlC	10	54.7	20.766	85.138	1.03E+10	89	181	28.4617	28.6063	28.3153	28.5201	28.7475	28.6506	28.7412	28.47
phate thym	Q9HU22	rmlA	14	65.5	32.457	90.736	6.66E+09	91	293	26.85	27.1207	27.3247	26.8397	27.0678	27.0207	27.0116	26.8032
	Q9HU23	rmlD	6	26.8	33.106	35.563	7.71E+08	40	302	24.9247	24.8102	24.7977	24.877	24.908	25.0309	25.5888	25.6307
cose 4,6-de	Q9HU24	rmlB	19	74.7	39.542	323.31	1.22E+10	157	352	27.9416	28.0525	28.1048	27.6797	27.8246	27.9802	28.3362	28.2371
	Q9HU31	PA5153	14	71.6	27.561	91.192	3.66E+09	87	250	26.843	27.3549	26.0079	26.3856	26.3153	26.3347	26.7769	26.7412
	Q9HU35	PA5149	6	31.9	39.433	21.771	1.41E+08	14	360	23.6723	23.8702	24.126	23.0319	22.1289	23.3342	23.7492	23.5317
(2+)-traffic	Q9HU36	PA5148	11	70	10.625	87.728	9.91E+09	94	90	29.4534	28.131	27.3251	29.2747	28.2989	29.085	28.2706	28.9537
	Q9HU38	PA5146	14	27.5	80.849	61.977	3.6E+08	37	750	23.6925	24.0152	24.0401	23.3352	24.5437	24.0091	24.1726	24.4553
ol-phospha	Q9HU41	hisB	7	45.7	21.937	45.308	1.86E+09	57	197	26.397	26.3251	24.8076	26.6824	26.2938	26.8207	26.3222	26.6998
osphate sy	Q9HU42	hisH1	15	90.1	23.698	181.8	1.11E+10	116	213	28.4824	28.6933	28.727	28.7113	28.9409	28.5574	28.6805	28.5122
o)methylid	Q9HU43	hisA	20	89.4	25.938	251.2	2.43E+10	158	245	29.4645	29.7281	29.4818	29.603	29.5675	29.4852	29.5123	29.4487
osphate sy	Q9HU44	hisF1	13	77.7	27.131	158.58	8.19E+09	105	256	28.1079	28.1173	27.6632	28.2132	28.058	28.1272	27.9758	28.0951
	Q9HU49	PA5135	4	28.4	28.071	10.662	3.79E+08	12	257	23.9437	25.0807	23.5674	24.2353	23.4599	24.7579	25.0217	24.972
	Q9HU50	PA5134	21	71.3	46.018	209.54	2.02E+10	204	436	28.6558	28.8339	28.9419	28.7066	28.6678	28.6916	28.8511	28.6206
	Q9HU51	PA5133	10	35.3	47.291	33.541	1.27E+09	43	428	25.9041	25.8057	25.9405	26.0558	25.9097	26.0222	25.744	26.3266
ndependent	Q9HU53	gpmI	25	69.1	55.6	315.42	3.92E+10	265	515	29.5478	29.5959	29.7576	29.5292	29.7522	29.8412	29.7125	29.6258
lutaredoxi	Q9HU55	grx	5	65.5	9.1926	84.488	3.47E+09	38	84	28.3715	26.3617	26.1059	28.5138	26.0412	28.1089	26.8223	28.0558
export prot	Q9HU56	secB	10	84.7	18.146	270.4	4.34E+10	189	163	30.323	30.261	30.4243	30.5036	30.571	30.3107	30.4454	30.2588
	Q9HU59	ntrC	16	49.2	52.753	118.23	1.95E+09	74	476	25.6362	25.8063	26.0139	25.4063	24.9571	25.1081	25.4129	25.397
mine synth	Q9HU65	glnA	30	68.2	51.944	265.88	5.5E+10	353	469	29.5796	29.7966	29.9166	29.3438	29.2117	29.2631	29.4743	29.4091
sulfurtrans	Q9HU66	thiI	7	24.2	54.849	27.776	6.13E+08	34	484	25.2051	25.7212	25.418	25.4783	25.328	25.6098	25.7051	26.1221
	Q9HU67	typA	26	46.4	66.934	323.31	3.21E+10	224	605	29.6004	29.6668	29.7958	29.5626	29.5674	29.5911	29.5275	29.6449
lglutathion	Q9HU72	gloA	6	61.9	20.214	49.213	3.95E+09	46	176	27.3988	27.1225	26.6472	27.3962	27.5818	27.5699	27.6604	27.6253

-bisphosph	Q9HU73	fbp	15	66.7	37.216	249.53	2.21E+10	168	336	28.8834	29.0109	29.1598	28.8781	28.6859	28.8997	29.0153	28.7904
e iminopep	Q9HUA3	PA5080	19	77.7	36.515	201.62	1.47E+10	156	323	28.1669	28.3446	28.4348	28.112	27.9136	27.8596	27.8968	27.649
iosynthesis	Q9HUA5	opgG	28	60.4	59.476	236.79	4.45E+10	287	525	28.8492	28.9787	29.0976	28.7645	28.8772	28.8577	28.954	28.8471
hesis gluco	Q9HUA6	opgH	6	13.9	96.994	35.369	4.62E+08	32	861	23.5472	24.2931	22.0489	24.2883	24.8145	24.5037	24.5784	24.7506
	Q9HUA7	PA5076	20	74.8	29.736	222.4	2.54E+10	179	266	28.8839	28.7491	28.9401	28.8006	28.9548	28.9096	28.695	28.8292
	Q9HUA9	PA5074	3	23.8	27.075	15.223	3.11E+08	20	244	24.2669	23.784	24.7014	24.1553	24.3261	24.2756	24.3748	24.1977
yl-ATP pyro	Q9HUB6	hisE	3	21.6	12.038	10.701	7.52E+08	18	111	24.2619	23.6646	23.5931	26.6819	26.0771	26.1854	23.6158	26.4058
syl-AMP cy	Q9HUB7	hisI	6	74.6	15.375	51.481	3.79E+09	47	134	27.1846	26.9969	26.2392	27.001	27.0735	26.9374	26.668	26.8978
	Q9HUB9	PA5064	3	18.8	22.767	24.017	6.83E+08	24	208	25.926	25.1846	25.7669	26.0839	25.5247	25.6091	25.5366	25.6642
biosynthesi	Q9HUC0	ubiE	14	66.8	28.254	161.14	1.18E+10	129	256	28.0564	28.3541	28.4844	27.9508	27.7225	27.7956	27.8925	27.6719
	Q9HUC1	PA5062	3	48.4	9.8063	20.871	1.21E+09	26	91	26.4972	26.0535	25.4916	26.6299	25.8866	26.2258	25.5124	25.8794
	Q9HUC3	phaF	5	22.7	30.577	11.229	8.18E+08	22	309	23.2357	25.4232	24.476	25.4459	25.8517	25.7088	25.7567	25.8276
rotease ATI	Q9HUC5	hslU	29	52.6	50.082	191.17	2.66E+10	211	447	28.9072	28.9994	29.2232	28.7692	28.6561	28.8264	28.8154	28.8133
nt protease	Q9HUC6	hslV	5	54.8	18.785	106.5	4.46E+09	46	177	27.7038	27.2111	27.4217	27.5286	27.942	28.0618	27.832	27.8496
ine--tRNA l	Q9HUC8	argS	22	43.8	65.199	164.8	8.04E+09	147	587	26.8229	27.2346	26.5565	27.1037	26.9764	26.7595	26.4557	26.8805
osomal pro	Q9HUD0	rpmE	6	74.6	7.9201	85.533	8.03E+10	145	71	32.1242	31.5366	30.9521	32.5271	31.6811	31.8053	31.7501	32.1638
	Q9HUD3	PA5046	39	85.3	45.428	323.31	3.06E+11	640	422	32.0858	31.9537	32.1641	32.1078	32.438	32.2105	32.3612	32.2145
	Q9HUD4	PA5037	15	44.6	57.446	117.56	1.74E+09	64	551	25.6587	25.8396	25.3109	25.8925	26.0967	25.6803	25.4936	25.5879
	Q9HUE1	PA5028	11	73.7	28.643	56.972	1.37E+09	42	255	26.2832	26.3132	26.2991	26.0207	25.7951	26.3597	26.2127	26.0355
ge subunit r	Q9HUF0	rlmJ	8	42.8	31.882	33.469	3.53E+08	27	278	24.2069	24.6368	23.0474	24.2996	24.2848	24.3331	24.6627	24.9247
ne sulfoxid	Q9HUF1	msrA	9	59.5	23.517	63.966	8.32E+09	77	215	28.7689	28.6898	28.2155	28.8385	28.4556	28.4098	28.2873	28.1396
	Q9HUF5	waaC	4	20.6	39.746	14.269	3.66E+08	24	355	24.9153	24.6778	22.4226	24.7132	24.7111	24.6631	24.7151	24.7774
	Q9HUF6	waaG	4	23.3	42.156	14.695	1.64E+08	17	373	22.4884	23.0135	22.7593	24.6922	24.3921	23.8628	24.3697	24.5959
	Q9HUF8	PA5007	11	70.2	29.414	48.124	1.06E+09	66	252	25.0057	25.0595	25.3632	24.7231	24.6934	24.8692	25.0831	25.0215
	Q9HUG0	PA5005	18	49.6	66.079	130.26	4.33E+09	85	585	26.8913	27.4599	26.221	27.2533	27.2897	27.0622	26.8353	27.5922
	Q9HUG1	PA5004	8	33.6	42.197	35.419	4.88E+08	27	378	24.2757	24.201	24.2392	24.1045	24.6545	24.5678	24.2855	24.9596
	Q9HUG2	PA5003	6	37.2	33.849	60.434	3.22E+08	18	298	23.0818	25.0941	22.9736	23.8132	25.2816	23.6081	25.0303	25.1874
	Q9HUG7	PA4998	5	28.2	24.985	6.3834	3.36E+08	18	216	24.0819	24.3547	24.3637	23.321	24.8569	23.9869	23.764	24.0156
inding/peri	Q9HUG8	msbA	5	14.3	66.431	23.945	2.05E+08	14	603	23.2395	23.8704	23.8677	23.3638	24.2674	23.8133	23.9488	24.3575
hate adeny	Q9HUG9	hldE	21	57.4	50.348	203.61	1.26E+10	151	474	27.9257	28.0054	28.0267	27.6353	27.8479	27.9288	27.8318	27.8245
	Q9HUH3	PA4992	12	59.6	29.03	113.95	1.65E+09	50	270	25.8533	26.3421	26.2961	25.9903	26.291	26.0689	26.2345	25.6675
	Q9HUH4	PA4991	15	61.1	42.109	136.15	6.19E+09	78	391	28.7263	28.7697	28.7516	28.9039	28.8604	28.7573	28.712	28.5035
	Q9HUJ1	PA4974	19	48.3	53.405	142.42	2.68E+09	101	482	25.5088	25.3827	25.1288	25.5135	26.0505	25.4303	26.1951	25.5583
	Q9HUJ4	aspP	10	59	23.133	112.02	3.63E+09	86	205	26.7356	26.76	26.6672	26.6224	26.6834	26.8573	26.5999	26.754
ophosphat	Q9HUJ6	cpdA	9	57.7	30.516	101.6	1.37E+09	46	272	26.118	24.7087	23.3855	25.9912	25.3947	26.21	23.8071	26.0474
	Q9HUJ7	PA4968	7	60.7	22.645	36.282	1.14E+09	54	206	25.2479	25.3825	25.3567	25.1375	25.3134	25.388	25.21	25.2148
somerase 4	Q9HUJ8	parE	28	56.8	69.178	140.72	4.48E+09	137	629	26.1476	26.2396	26.3457	26.0691	26.6046	26.5995	26.7378	26.7146
	Q9HUJ9	PA4966	13	55.8	36.483	39.519	1.41E+09	48	337	25.4488	25.4893	24.3892	25.4468	25.3496	25.2354	25.2019	25.0472
somerase 4	Q9HUK1	parC	28	52.1	83.364	187.66	7.97E+09	144	754	28.099	28.5854	27.9159	28.5382	28.9034	28.7769	28.6353	28.9356
	Q9HUK5	PA4960	8	24.7	46.438	40.507	1.49E+09	33	429	26.1212	26.3729	26.2325	26.0786	26.1876	26.1496	26.0284	26.189
	Q9HUK6	fimX	4	9.8	75.992	60.298	3.45E+08	24	691	24.0813	24.7139	24.211	24.0964	24.9492	24.9443	24.1765	24.7108
	Q9HUK7	PA4958	20	62.6	67.828	102.32	2.3E+09	81	607	25.4592	25.805	25.2907	25.6787	25.794	25.7939	25.7157	25.8963
furtransfer	Q9HUK9	rhdA	12	70.8	29.391	101.1	8.53E+09	82	271	27.6965	27.7199	27.4959	27.8369	27.8143	27.7798	27.6486	27.6831
ne biogene	Q9HUL3	rsgA	12	54.3	37.078	102.02	2.29E+09	76	339	26.1858	26.1198	26.4147	26.0942	26.1339	26.1447	25.8793	26.0586
	Q9HUL6	PA4948	4	40.6	16.495	34.079	4.89E+08	16	155	25.2885	23.8008	24.7105	24.9338	23.1509	22.2817	25.4942	25.1286
	Q9HUL7	amiB	15	49.1	50.683	131.09	3.62E+09	89	475	26.7898	27.0027	27.2075	26.8009	26.6611	26.8403	26.7754	26.6186
ch repair p	Q9HUL8	mutL	8	28	69.747	67.303	8.33E+08	41	633	24.6618	25.1702	25.0288	24.9234	24.9962	25.088	25.2089	25.7971
GTPase HflX	Q9HUM1	hflX	15	52	48.607	111.38	1.47E+09	71	433	26.0698	25.7954	26.1644	26.0256	25.5154	25.8955	25.94	26.0935
transferase	Q9HUM5	hisZ	15	48.7	42.546	159.99	9.89E+09	100	394	28.5523	28.614	28.7555	28.3414	28.6647	28.8764	28.9768	28.9262
uccinate sy	Q9HUM6	purA	28	65.8	46.814	323.31	5.67E+10	332	430	29.834	30.0598	29.9563	29.4988	29.6017	30.0108	29.9577	30.1832
-2'-O-)-me	Q9HUM8	rlmB	11	60.1	27.081	58.602	2.91E+09	48	248	27.3305	27.6273	27.3545	27.277	27.2171	27.1841	27.328	27.2117
osomal pro	Q9HUM9	rpsF	14	69.1	16.164	323.31	2.56E+11	488	139	32.3404	32.4029	32.0348	32.3267	32.4076	32.4105	32.3763	32.3138
osomal pro	Q9HUN0	rpsR	4	40.8	8.8732	44.39	1.7E+10	50	76	29.6161	30.6092	29.8269	30.3993	30.9369	30.3557	30.609	30.6838
osomal pro	Q9HUN2	rplI	11	73	15.532	157.41	3.42E+11	700	148	33.046	32.9952	32.6082	33.2693	33.2052	33.1098	32.9286	33.0546
	Q9HUN3	dnaB	24	67	51.589	156.35	2.64E+09	114	464	25.5624	25.6394	25.6064	25.2831	25.5276	25.6368	25.8832	26.0183
cemase, bi	Q9HUN4	alr	9	38.3	38.31	101.5	3.74E+09	83	358	26.8742	26.5871	26.3447	26.8092	26.7554	26.9096	26.4658	26.7073
dent NAD(-	Q9HUP3	nadE	17	82.9	29.696	294.38	2.11E+10	173	275	29.3738	29.1475	28.6598	29.328	28.6805	28.7813	28.5337	28.6603
osphoribosy	Q9HUP4	pncB1	14	46.6	46.094	84.819	1.19E+09	45	399	25.5604	25.5516	24.753	25.6792	25.4074	25.0559	25.3222	23.2228
	Q9HUP7	PA4916	7	39.8	25.457	42.213	1.3E+09	47	231	26.2122	26.2857	26.0326	26.2337	25.797	25.9216	25.6219	25.7181
	Q9HUQ6	PA4907	12	65.2	27.384	210.65	1.09E+10	99	253	27.8426	28.0648	28.3713	27.7549	27.6927	27.7679	27.8418	27.6514
	Q9HUR4	PA4899	5	23.9	52.697	37.003	4.41E+08	28	489	24.6047	24.5253	24.997	24.3407	24.0886	23.6949	24.2574	24.3025
	Q9HUS3	desT	9	60.3	22.899	23.577	6.36E+08	36	209	24.6373	24.9449	25.1398	24.9398	24.9756	25.15	24.4905	23.648
	Q9HUT3	PA4880	12	76.3	20.217	214.21	6.79E+09	85	177	27.6822	27.8777	27.6315	27.7715	28.1259	27.7787	27.8743	27.6125
	Q9HUT5	PA4878	5	36.7	31.168	41.841	1.63E+09	24	270	27.4732	25.9983	23.4214	27.5707	26.2334	27.3196	26.1321	26.9995
se subunit a	Q9HUU5	ureC	6	23.1	60.61	24.805	3.62E+08	24	566	24.437	24.2691	24.6761	23.8284	24.6261	24.4941	24.7993	24.7652
sylamine--g	Q9HUV8	purD	19	62.9	45.212	277.07	2.23E+10	190	429	28.3133	28.3503	28.3164	28.3015	28.3409	28.5082	28.5646	28.5188
lrolase;Pho	Q9HUV9	purH	32	63.9	57.665	323.31	3.07E+10	308	535	28.6089	28.7555	28.8464	28.4829	28.8075	28.9405	28.9409	28.7806
ike DNA-bi	Q9HUW0	PA4853	5	81.7	11.578	36.202	2.35E+09	29	104	27.6658	26.5399	25.8883	27.7551	26.8657	27.6476	26.4696	27.5141
	Q9HUX0	PA4840	4	46.3	12.663	62.443	7.4E+08	29	123	25.8142	21.6664	25.1799	25.6053	25.9719	25.3808	25.8735	25.4265
arginine de	Q9HUX1	speA	26	61.2	70.667	261.4	9.66E+09	215	636	26.7133	26.797	26.5445	26.654	26.8051	27.0591	26.8347	27.0244
	Q9HUX9	PA4831	4	28.5	20.48	21.054	2.75E+08	22	186	24.0518	24.4272	24.3088	24.3062	23.8436	24.2769	23.0857	24.0572
	Q9HUY3	PA4827	10	47.3	31.203	56.619	1.12E+09	50	279	25.8704	25.6661	25.8922	25.5295	25.3304	25.1443	25.6526	25.7185
ec) seleniu	Q9HV01	selA	9	22.9	50.291	30.148	1.94E+08	18	468	23.911	23.7717	24.0463	23.6077	24.4889	23.5792	23.4039	23.5043
	Q9HV22	PA4786	7	28.4	47.182	25.591	6.12E+08	32	451	24.8194	25.1259	25.4014	24.6355	24.7778	25.3351	25.3271	25.0469
	Q9HV32	pmrA	8	57	24.394	169.94	7.96E+09	67	221	28.0814	28.2129	28.4062	28.2033	28.2812	28.3834	28.2965	28.2798
te dehydro	Q9HV37	lldD	18	68.5	41.122	80.747	4.02E+09	97	381	26.8497	26.8882	26.8289	26.4447	25.7736	26.4439	26.0977	26.6798
-binding pro	Q9HV40	smpB	4	38.4	18.032	37.949	1.12E+09	18	159	25.8938	26.072	25.4364	25.9702	26.1213	26.5674	25.8798	24.079
Protein Grp	Q9HV42	grpE	12	84.9	20.702	239.71	7.52E+10	216	186	31.2713	30.5707	29.5262	31.539	30.4121	31.1948	29.9644	31.1077
rone protei	Q9HV43	dnaK	52	72.8	68.402	323.31	3.51E+11	906	637	31.7693	31.8073	31.6229	31.7121	31.7551	31.7506	31.8474	31.767
rone protei	Q9HV44	dnaJ	19	45.4	40.26	134.78	1.15E+10	143	377	29.4022	29.3239	28.6352	29.2556	29.2229	29.245	28.9846	29.0155
elongatio	Q9HV46	greA	10	82.3	17.158	133.18	5.22E+10	135	158	30.7322	30.4814	29.0062	30.9781	30.7109	30.9566	30.2381	30.8317
zinc metal	Q9HV48	ftsH	19	45.9	69.954	112.12	2.83E+09	116	639	25.2468	25.6956	24.663	25.6914	26.3111	25.6979	25.7797	26.2391
opteroate s	Q9HV49	folP	12	62.2	30.49	104.75	3.27E+09	77	283	25.8946	26.1014	26.111	25.7618	26.3095	26.4124	26.5244	26.4045
osphate is	Q9HV51	tpiA	13	78.1	25.621	217.06	1.28E+10	138	251	28.2783	28.4849	28.357	28.4022	28.5134	28.7146	28.7438	28.6651
membran	Q9HV52	secG	3	38.8	13.207	23.057	1.65E+09	27	129	27.002	27.1791	25.9518	27.4763	27.2852	27.4229	26.5703	27.3023
naturation	Q9HV53	rimP	4	37.5	17.171	72.908	7.37E+09	35	152	28.7745	28.3518	28.4633	29.0652	28.1071	28.6683	29.0043	28.965
ion/antiter	Q9HV54	nusA	27	53.8	54.658	323.31	9.9E+10	354	493	30.8508	30.9579	31.1092	30.857	30.9602	30.9196	31.0931	30.9231
n initiation	Q9HV55	infB	59	70.1	90.911	323.31	1.28E+11	612	840	30.1959	30.3157	30.1706	30.1017	30.141	30.323	30.2058	30.2398
ne-binding	Q9HV56	rbfA	8	58.1	14.547	77.735	1.25E+10	82	129	28.2787	28.3064	28.2721	28.3742	28.3167	28.2574	28.1831	28.0462
osomal pro	Q9HV58	rpsO	4	48.3	10.115	74.843	2.11E+10	50	89	29.785	30.0326	29.6202	29.9588	29.8514	29.9161	30.1673	29.8656
tide nucleo	Q9HV59	pnp	49	74.6	75.452	323.31	1.91E+11	675	701	31.5388	31.5448	31.8336	30.8475	31.1534	31.569	31.7791	31.8175
	Q9HV60	PA4739	4	43	11.76	19.918	2.99E+08	18	114	23.1056	24.575	22.7226	24.908	24.8692	22.8477	24.7738	24.5232
	Q9HV63	PA4736	6	57.7	11.02	34.707	8.25E+08	30	97	25.8318	25.7262	24.8669	25.6192	25.1578	24.9596	24.9758	25.2454
phosphate	Q9HV67	pgi	23	56.5	61.944	178.73	7.99E+09	169	554	26.8446	26.9812	26.7447	26.7161	27.2272	27.0031	27.019	26.6871
henate synt	Q9HV69	panC	14	60.1	30.836	110.69	9.84E+09	105	283	27.7536	27.585	27.8592	27.5538	27.4958	27.67	27.441	27.7004
A) polymer	Q9HV72	pcnB	12	30.8	53.3	63.012	5.63E+08	31	467	23.7419	24.3206	24.209	24.5277	24.5579	24.8476	24.2823	24.8969
	Q9HV73	cbrB	17	46.9	52.286	176.36	7.77E+09	138	478	27.5741	27.5735	27.8341	27.2493	27.1739	27.3212	27.5051	27.3729
	Q9HV76	PA4722	11	49.5	42.567	64.459	1.56E+09	47	390	25.8161	25.9092	25.9078	25.5315	25.9285	26.1424	25.9419	26.3831
acil-C(5))-m	Q9HV78	trmA	12	54	41.206	181.06	3.79E+09	90	363	26.3663	26.4191	25.7587	26.5156	26.6203	26.5859	26.489	26.5774
	Q9HV81	PA4717	5	32.7	31.86	55.006	7.98E+08	44	297	24.9383	25.0101	25.1013	25.0262	24.7828	24.8755	25.3191	24.8715
	Q9HV83	PA4715	14	46.2	46.063	103.18	1.71E+09	39	411	26.1507	26.0823	26.1361	26.044	25.6456	25.8895	25.6712	25.574
	Q9HV90	phuT	10	62	31.037	52.456	2.29E+09	53	297	25.4222	25.1866	24.7545	25.7379	26.0569	25.8335	28.1001	26.1868
	Q9HV96	PA4701	28	75.8	58.376	323.31	1.48E+10	242	520	27.3748	27.3637	26.7525	28.0525	28.1121	27.8676	27.6268	27.2594
	Q9HV98	PA4698	2	26.6	11.994	14.448	4.92E+08	14	109	25.7397	23.9103	23.1473	25.8523	25.0253	25.8441	22.6363	25.6559
	Q9HVA0	ilvI	28	49.5	63.018	126.07	1.5E+10	174	574	28.2283	28.4241	28.6146	27.8891	28.2833	28.5496	28.6291	28.4252
	Q9HVA1	ilvH	4	39.3	17.784	96.196	5.41E+09	64	163	27.7672	27.8794	28.3393	27.9474	28.0732	27.9961	27.9442	27.6798
d reductois	Q9HVA2	ilvC	23	70.7	36.424	323.31	5.15E+10	258	338	29.0197	29.4962	27.6917	29.3577	29.8644	29.7431	29.5917	29.6658
	Q9HVA8	hitA	18	70.4	36.149	279.39	1.81E+10	171	335	28.7059	28.766	28.5644	28.8811	28.2355	28.2011	28.3487	28.1653
	Q9HVA9	PA4686	16	27	106.83	101.26	1.7E+09	68	948	24.5474	25.2325	24.6645	25.2539	25.7784	25.4097	25.1179	26.0713
	Q9HVB0	PA4685	8	47.2	26.354	84.67	2.51E+09	55	231	26.6724	26.5449	26.3843	26.6595	26.768	27.036	26.9162	26.663
	Q9HVB1	PA4684	10	36.8	49.194	42.957	9.98E+08	45	432	24.8746	24.9676	24.953	24.4612	24.8478	25.1583	25.3467	25.6568
	Q9HVB6	PA4679	8	57.1	24.873	43.552	5.3E+08	41	233	24.314	24.5513	24.5748	24.1907	24.4786	24.3285	24.3626	24.2842
	Q9HVB7	rimI	4	31.3	17.095	6.3095	3.29E+08	15	150	25.1235	24.9008	25.059	25.2284	23.574	23.4191	25.2979	25.2773
onic anhyd	Q9HVB9	PA4676	12	71.2	24.223	180.09	6.48E+09	83	215	27.7829	27.7713	27.2698	27.6722	27.943	28.0047	27.7581	27.6995
-binding AT	Q9HVC2	ychF	25	81.7	40.077	219.86	3.99E+10	225	366	29.1697	29.2809	29.2891	29.0328	29.164	29.2973	29.3655	29.2608
yl-tRNA hyd	Q9HVC3	pth	8	59.3	20.804	44.843	6.55E+08	35	194	24.4185	24.7981	24.9761	24.7937	25.0429	24.9711	24.8135	24.8066
osomal pro	Q9HVC4	rplY	18	81.9	21.962	323.31	5.01E+11	469	204	33.2808	33.426	33.3031	33.2275	33.1462	33.3408	33.2539	33.2105
hate pyrop	Q9HVC5	prs	20	78.6	34.04	323.31	7.47E+10	288	313	30.3613	30.4484	30.6256	30.1306	30.5051	30.56	30.6052	30.3541
utamine m	Q9HVC8	prmC	4	21.7	30.486	20.769	6.11E+08	13	276	25.3031	25.5218	25.3404	25.4416	25.3044	25.3565	23.323	25.5483
amate race	Q9HVD0	murI	8	41.5	28.274	45.841	1.28E+09	36	265	26.2528	25.9848	26.3567	26.0478	26.2304	26.2586	26.3776	26.1522
	Q9HVD5	PA4657	13	61.2	35.913	152.8	3.15E+09	94	327	26.5025	26.6726	27.1891	26.7657	26.4456	26.1211	26.7554	26.2035
phoribosyl	Q9HVE6	upp	17	70.8	22.938	95.501	1.66E+10	151	212	28.3149	28.3174	28.4517	28.4402	28.1901	28.1456	28.1594	28.0777
	Q9HVE7	PA4645	13	72.4	20.639	136.97	2.36E+10	159	185	29.5746	29.6432	29.6393	29.4852	29.3843	29.2928	29.2603	29.1529
	Q9HVE8	PA4644	8	82.8	17.109	95.976	4.39E+09	54	157	27.9513	27.1822	26.5786	28.1447	27.2849	27.7362	27.1021	27.7833

	Q9HVF0	PA4642	3	43.8	10.8	28.353	6.03E+09	40	96	28.6376	27.8802	26.7858	28.8302	28.0677	28.768	27.6693	28.6299
:quinone c	Q9HVF1	mqo2	14	37.9	55.498	97.961	7.44E+09	94	507	28.1857	28.1169	28.2765	27.6217	27.5942	28.048	28.1451	28.3209
	Q9HVF5	PA4636	10	43	43.779	58.857	1.35E+09	47	386	25.427	25.6603	25.684	25.544	25.3673	25.8034	25.7775	26.27
	Q9HVF9	PA4632	6	37.4	29.159	47.577	4.35E+08	26	273	24.3602	24.6154	22.3779	24.8521	24.9439	24.739	24.6423	24.9384
	Q9HVG0	PA4631	9	46	37.263	69.493	7.56E+08	40	341	24.9257	25.1748	25.3078	25.1456	25.022	25.029	24.9823	24.9298
ll subunit r	Q9HVG4	rsmC	7	28.9	35.937	22.733	3.28E+08	23	332	24.0825	24.5403	23.3347	23.7602	24.2637	23.9932	24.5269	24.0488
	Q9HVG5	hprA	6	27.9	34.285	21.924	6.11E+08	33	323	24.681	25.1829	24.9974	24.5112	25.0377	25.3289	25.2466	25.182
	Q9HVH1	PA4620	3	21.2	19.918	21.723	3.06E+08	21	179	24.6485	24.3471	22.5739	24.7894	24.6396	24.5979	24.0127	24.231
	Q9HVH2	PA4619	11	41	44.71	141.03	1.16E+09	73	415	25.3598	25.2966	24.8355	25.4939	24.9744	24.7798	23.521	24.8332
	Q9HVH6	PA4615	11	56.2	29.377	72.136	2.69E+09	75	258	25.5938	26.0019	25.9364	25.7254	26.1046	25.5655	25.8272	25.4933
	Q9HVH9	PA4611	4	60.7	9.7158	55.783	7.69E+09	44	84	30.0844	28.9018	27.9325	30.7592	26.4082	28.9882	27.1323	28.4211
	Q9HVI5	PA4604	4	20.1	37.256	14.443	2.89E+08	19	334	23.749	24.6567	24.57	22.9966	24.7302	24.6658	23.7713	24.7945
oxymethylt	Q9HVI7	glyA2	13	52.8	45.157	144.21	2.63E+10	149	417	30.8324	30.4392	30.7372	30.5818	31.0211	31.0615	30.9854	30.9339
	Q9HVJ1	PA4595	36	63.2	61.32	323.31	5.63E+10	356	554	29.6756	29.7911	29.8243	29.3769	29.6899	29.866	29.8494	29.9004
	Q9HVK8	PA4576	22	36.4	90.222	195.48	3.02E+09	104	817	25.4043	25.6508	25.3107	25.4523	25.6354	25.5283	25.6838	26.0378
olyl cis-tran	Q9HVL2	fklB	7	36.6	21.795	163.93	1.58E+10	71	205	30.0563	29.4382	28.5499	30.0321	29.1574	29.8458	29.1827	29.8565
	Q9HVL5	ispB	14	62.7	34.851	143.28	3.57E+09	86	322	26.8352	26.7953	26.9114	26.461	26.7883	26.9659	26.9711	27.2526
osomal pro	Q9HVL6	rplU	14	77.7	11.653	96.902	9.22E+10	189	103	30.9321	31.2967	31.1509	30.9802	31.2029	31.0961	31.1338	31.0741
osomal pro	Q9HVL7	rpmA	5	51.8	8.9903	10.174	2.46E+09	19	85	27.0534	27.6252	23.6087	27.1681	27.6911	27.346	27.8418	27.6363
GTPase Ob	Q9HVL8	obg	20	64	44.338	217.66	1.26E+10	127	406	28.1216	28.2414	28.405	28.1507	27.9874	28.044	28.1098	27.9539
amate 5-ki	Q9HVL9	proB	8	33.9	39.845	91.226	1.36E+09	60	372	25.4192	25.3272	25.6866	25.4934	25.4664	25.8013	25.6684	25.9397
	Q9HVM0	PA4564	7	55.2	16.724	48.998	1.63E+09	39	154	26.4176	26.4461	26.459	25.879	26.0003	26.2084	25.9354	25.9403
osomal pro	Q9HVM1	rpsT	1	15.4	9.9175	9.4061	8.54E+09	15	91	29.4262	30.2405	22.7422	30.2771	30.6414	30.3719	30.4749	22.849
biosynthes	Q9HVM3	ribF	11	44.6	34.34	49.437	8.8E+08	27	312	25.8108	25.4401	25.8629	25.9841	26.6034	25.6132	26.2534	25.5765
olyl cis-tran	Q9HVM6	PA4558	7	68.5	15.984	115.2	1.47E+10	75	146	29.3125	28.6543	27.5819	29.3909	28.9821	29.5365	28.8952	29.4351
ut-2-enyl d	Q9HVM7	ispH	14	51	34.762	99.852	4.27E+09	86	314	26.9274	26.9875	26.5187	27.0686	27.1724	27.1468	27.1367	26.9397
rone protei	Q9HVN5	clpB	66	74.4	95.004	323.31	6.37E+10	550	854	29.2904	29.2782	29.497	29.0888	28.7367	28.9675	28.9697	28.8575
	Q9HVP4	PA4533	5	32.1	17.645	14.498	9.52E+08	27	156	26.2337	26.3825	26.6539	26.3933	26.2148	26.3564	26.4373	25.9572
ospho-CoA	Q9HVP8	coaE	8	66	22.843	77.075	2.33E+09	46	203	26.3041	26.3103	25.7418	26.5458	26.2559	26.3599	26.1215	26.2028
	Q9HVQ3	speC	19	66.7	43.559	216.69	7.76E+09	142	387	26.895	26.8418	27.0985	26.824	27.0995	27.0432	27.28	27.0049
	Q9HVR5	PA4506	6	28.5	36.339	31.284	4.06E+08	23	323	24.4728	24.6792	25.1755	24.0435	24.5082	22.6432	23.9714	23.105
	Q9HVR6	PA4505	7	38	35.429	30.371	3.55E+08	23	324	25.0152	24.6097	25.4346	24.2005	24.2266	24.32	24.2414	23.199
	Q9HVS2	PA4499	4	41.7	20.435	9.111	2.5E+08	14	187	24.6734	24.6574	22.8141	23.4409	24.5146	24.8157	23.0691	24.8641
	Q9HVS3	PA4498	10	40	44.126	74.519	4.5E+08	34	405	24.674	24.6845	24.5242	24.744	24.3947	23.9142	23.8245	24.3909
	Q9HVS6	PA4495	11	54.7	24.879	191.25	2.34E+10	113	236	29.2942	29.4281	28.9605	29.4447	29.3474	29.1631	29.888	29.0055
	Q9HVS8	roxR	8	71	20.616	93.854	1.58E+10	71	186	29.0087	29.0918	29.3541	28.9759	28.7415	28.8527	28.9134	28.8672
	Q9HVT4	PA4487	5	42.2	28.145	48.71	5.46E+08	13	263	23.7879	25.9502	23.2361	23.4937	22.3225	25.3943	25.7635	25.5523
	Q9HVT5	PA4486	7	76.6	13.947	21.311	1.04E+09	17	128	26.3173	22.693	23.7782	26.3439	25.3157	26.0595	25.7606	25.9685
sn/Gln) an	Q9HVT7	gatB	26	60.5	53.081	260.59	1.67E+10	228	481	28.062	28.1006	28.0321	28.0145	28.0002	28.2179	28.1604	28.2611
) amidotra	Q9HVT8	gatA	24	71.7	51.862	256.58	2E+10	180	484	28.5553	28.4613	28.6736	28.2335	28.4336	28.8051	28.7354	28.9561
) amidotra	Q9HVT9	gatC	4	79.2	10.53	41.041	2.51E+09	42	96	27.4793	24.18	24.3083	27.0728	24.0416	27.4564	24.5642	25.8477
	Q9HVU0	mreB	21	57.4	36.976	312.1	2.73E+10	184	345	28.5402	28.7226	28.7318	28.292	28.5942	28.8017	28.8873	28.9946
	Q9HVU4	cafA	7	20.6	54.665	42.097	3.69E+08	27	485	23.5908	24.0841	23.8222	24.0939	24.1844	24.245	24.1782	23.7241
	Q9HVU6	PA4475	7	34	30.724	17.197	3.41E+08	16	282	24.3692	24.0723	24.6094	23.7928	23.902	23.0227	24.3909	23.7713
	Q9HVU7	PA4474	16	51.2	50.266	226.16	9.34E+09	123	480	27.4075	27.5413	27.664	27.4032	27.5647	27.3796	27.6	27.2288
)7 protein I	Q9HVU8	PA4473	7	52.3	20.21	42.234	2.12E+09	31	174	26.9166	27.0879	27.0925	26.7782	26.732	26.6339	26.8054	26.7247
	Q9HVU9	pmbA	30	82	47.908	323.31	1.99E+10	218	449	29.0344	29.0575	29.3775	29.1381	29.1756	29.0164	29.1496	28.6863
ocarrier pro	Q9HVV2	ptsH	4	60	9.7021	64.744	1.8E+09	29	90	26.9291	26.7738	26.4915	27.0784	26.5567	26.5835	26.6324	26.5091
oinding pro	Q9HVV3	PA4465	8	45.1	32.218	88.438	2.45E+09	53	286	26.4677	26.2906	26.7917	26.3083	26.155	26.1994	26.6224	26.3521
osphotran	Q9HVV4	ptsN	6	48.1	16.73	69.981	2.38E+09	41	154	27.0001	26.0958	25.46	27.1726	26.1544	27.0149	25.716	26.7762
	Q9HVV6	PA4461	12	56.8	26.45	106.01	1.04E+10	116	241	27.5888	27.7521	28.0553	27.5053	27.6386	27.8574	27.6992	27.634
e export sy	Q9HVV7	lptA	8	56	19.126	128.37	1.3E+10	79	175	29.3588	29.3086	29.0213	29.1867	29.5444	29.3724	29.4493	29.1207
e export sy	Q9HVV8	lptC	4	28.9	21.353	11.622	2.29E+08	15	190	24.1515	24.1349	24.2087	23.5276	24.5911	24.2881	24.2064	24.7101
	Q9HVV9	PA4458	8	44.7	19.334	59.748	4.04E+09	66	179	27.1142	26.8259	26.947	27.0149	27.244	27.4279	27.0132	27.2592
hosphate is	Q9HVW0	kdsD	16	63.8	34.197	165.18	1.24E+10	136	326	28.058	28.2143	28.2364	28.0368	28.0227	28.0448	28.2525	28.1657
	Q9HVW1	PA4456	13	48.3	29.621	88.198	4.2E+09	77	269	26.1941	26.3828	26.4468	26.0448	26.3471	26.6387	26.5789	26.6633
	Q9HVW3	PA4454	5	47.8	16.557	29.798	8.45E+08	28	157	25.7871	22.7857	24.4047	25.5488	26.1881	26.2528	25.3801	26.0784
	Q9HVW4	PA4453	13	59.1	23.76	103.64	3.41E+10	136	215	30.257	30.1903	29.9942	30.4711	30.5986	30.576	30.7312	30.4461
	Q9HVW5	PA4452	3	49	10.777	90	1.64E+09	27	102	26.7296	26.5956	26.45	26.8175	26.9646	27.0595	26.8893	26.8083
	Q9HVW6	PA4451	4	77.2	8.8028	98.488	4.97E+09	37	79	28.5615	27.9345	27.9388	28.2056	27.8255	28.7164	28.4019	28.6924
mine 1-car	Q9HVW7	murA	28	71.5	44.645	323.31	3.49E+10	307	421	29.343	29.3425	29.4842	29.274	29.5666	29.4836	29.5039	29.2772
ohoribosylt	Q9HVW8	hisG	14	73	22.844	123.16	5.86E+09	99	211	26.5756	26.6595	26.691	26.9221	26.7993	26.7503	26.5456	26.4761
ol dehydrc	Q9HVW9	hisD	23	60.9	47.179	323.31	1.78E+10	198	440	28.0943	28.2008	28.3835	27.99	28.4304	28.3989	28.4354	28.2416
phate amir	Q9HVX0	hisC1	10	39.3	38.655	57.229	3.99E+09	56	351	27.5611	27.4742	27.5568	27.395	27.7734	27.9337	27.5862	27.8127
cyclohydrc	Q9HVX2	PA4445	12	73.8	27.36	169.47	2.82E+09	75	252	26.5855	26.4383	23.2405	26.1435	26.833	26.8049	26.343	26.7632
	Q9HVX4	PA4441	7	54.1	16.418	58.068	1.58E+09	46	148	25.5775	25.7969	25.3026	26.1667	25.5769	25.7709	25.3776	25.9472
	Q9HVX5	PA4440	8	69.4	22.333	83.444	1.06E+09	30	209	25.5475	25.644	25.5377	25.5608	25.623	25.8087	25.8245	25.7283
phan--tRN/	Q9HVX6	trpS	18	54.5	48.961	173.05	1.53E+10	128	448	28.8583	28.5824	29.034	28.6229	28.7797	29.0289	29.0504	28.9923
ision prote	Q9HVX7	zapE	17	56	41.993	86.042	2.27E+09	82	364	26.3202	26.3702	26.064	26.309	26.4995	26.3762	26.4832	26.5541
	Q9HVY1	PA4434	12	51	38.644	62.073	3E+09	67	345	26.4805	26.3568	26.4665	26.7248	26.7641	26.8555	26.384	26.6585
osomal pro	Q9HVY2	rplM	9	61.3	16.028	115.06	6.73E+10	139	142	31.1576	31.381	31.9278	31.2066	31.2953	31.1781	31.5083	31.0536
osomal prc	Q9HVY3	rpsI	6	44.6	14.615	47.215	1.23E+10	55	130	29.2993	29.7096	28.5266	29.6697	30.005	29.7809	29.4636	29.8757
	Q9HVY6	PA4429	12	58.8	28.993	85.202	5.41E+09	76	260	27.5775	27.9297	27.0112	28.0913	27.3498	27.5317	26.9575	27.8722
	Q9HVY7	sspA	8	42.9	23.615	74.51	3.21E+09	56	205	27.2929	27.1225	27.4597	27.1552	27.4325	27.4109	27.3835	27.2259
	Q9HVY8	sspB	6	82.2	14.548	62.062	3.56E+09	75	135	26.954	26.5979	25.8336	27.0595	26.9427	26.8881	26.4757	26.7612
heptose is	Q9HVZ0	gmhA	10	75.1	21.413	286.32	2.34E+10	96	197	29.2405	29.2453	28.4128	29.3385	29.6843	29.6786	29.3868	29.434
	Q9HVZ2	PA4423	14	51	65.629	136.58	1.9E+09	88	604	25.1524	25.2884	24.9274	25.351	25.5804	25.3017	25.7565	25.6556
all subunit	Q9HVZ3	rsmI	9	35.1	30.617	33.868	1.47E+09	53	282	25.6305	25.7616	25.6995	25.2576	25.5678	25.6863	25.6654	25.6702
ional regul	Q9HVZ4	mraZ	5	46.4	17.111	11.899	3.24E+08	13	151	24.984	24.5828	23.7701	25.1126	23.7998	24.7537	22.605	23.1393
ll subunit r	Q9HVZ5	rsmH	12	50.8	34.64	188.98	6.04E+09	121	313	26.9106	27.0638	27.2644	26.8546	26.8618	26.8377	26.99	26.655
ripeptide--	Q9HVZ7	murF	19	71.2	47.412	204.48	1.25E+10	173	458	27.7544	28.0331	28.1726	27.7718	27.8853	27.7086	27.7869	27.6953
noylalanine	Q9HVZ9	murD	19	55.8	48.08	130.16	7E+09	115	448	27.3347	27.4294	27.4319	27.4473	27.4401	27.4474	27.3141	27.1366
de) pyroph	Q9HW01	murG	11	48.2	37.798	68.739	1.7E+09	46	357	25.678	25.9894	25.823	25.7429	26.0538	26.2499	26.7727	26.2882
uramate--	Q9HW02	murC	11	32.9	51.981	59.434	2.53E+09	61	480	25.8102	25.8883	26.0288	25.6295	25.9896	26.2205	26.3904	26.611
nal protein	Q9HW04	argJ	19	79.5	41.762	191.54	2.09E+10	194	405	29.0598	28.9909	28.8877	29.0684	29.0487	29.2605	29.2139	29.1311
	Q9HW06	PA4400	17	66.3	34.054	142.61	5.66E+09	104	315	27.3854	27.6153	27.3746	27.3538	27.5529	27.6247	27.5987	27.4245
	Q9HW07	PA4399	4	31.8	21.065	28.171	3.67E+08	20	192	24.5635	24.5321	23.4684	24.2977	24.7885	24.5318	24.6166	24.5707
34 protein I	Q9HW11	PA4395	11	50.3	18.044	124.71	1.56E+10	82	159	29.8963	29.7143	30.0484	29.7522	29.3175	29.5266	29.7746	29.6267
	Q9HW15	speA	12	46.8	26.72	97.715	1.21E+10	68	252	28.8037	29.2849	29.5277	28.8625	28.8346	28.9301	29.2033	28.7689
	Q9HW16	PA4388	9	67.2	27	30.867	2.32E+09	45	244	27.1576	27.049	26.7641	27.0236	26.9234	27.1977	26.9435	26.8731
	Q9HW21	PA4381	8	51.5	25.235	93.189	3.49E+09	50	227	27.3787	27.3454	27.6819	27.4724	27.11	26.9369	27.7568	27.1894
	Q9HW29	PA4373	10	44	40.005	37.442	6.22E+08	28	364	24.1717	24.5088	24.4306	24.5338	24.9017	24.4715	24.8743	24.3977
	Q9HW41	PA4360	6	42.7	17.302	51.666	5.29E+08	24	157	24.7272	25.1702	23.8256	25.2393	24.8378	24.7796	25.2681	25.2125
	Q9HW44	PA4357	3	41.2	8.4948	13.523	4.04E+08	16	80	24.6597	22.5638	22.6646	24.7084	25.0718	25.1491	25.2715	23.7576
	Q9HW45	xenB	24	86.6	37.796	323.31	8.47E+10	293	350	30.5958	30.6277	30.6387	30.631	30.6574	30.3562	30.6749	30.1827
	Q9HW61	PA4340	3	31.1	20.988	17.369	3.94E+08	25	190	24.9694	24.0702	24.9625	24.7417	25.4129	25.1071	24.9352	24.7755
	Q9HW65	PA4336	9	86.6	21.025	137.63	1.16E+10	103	194	28.6562	28.5365	27.9258	28.5876	28.0929	28.0563	27.9959	27.8948
	Q9HW68	PA4333	26	75	54.763	299.78	4.57E+10	290	507	29.2489	29.3123	29.4559	29.0675	28.9515	29.3193	29.1665	29.4475
ruvate kina	Q9HW72	pykA	29	65.4	52.252	323.31	4.24E+10	289	483	29.027	29.1986	29.3032	28.8214	28.8894	28.9925	29.0455	28.9653
	Q9HW79	PA4322	16	61.5	36.661	132.94	6.21E+09	113	335	27.1969	27.3769	27.3801	26.9891	27.3988	27.6043	27.6239	27.5612
	Q9HW86	mvaT	9	68.5	14.18	46.565	3.73E+10	134	124	29.8297	30.451	30.3044	30.1271	30.1272	29.9826	30.5393	30.3083
ydrofolate	Q9HW87	purU1	14	67.5	32.41	110.05	7.38E+09	107	283	27.3153	27.6247	27.5954	27.2688	27.2964	27.574	27.7236	27.6964
g chemota	Q9HW91	pctB	12	31.2	68.076	256.11	3.54E+09	96	629	26.2545	26.7447	25.6782	26.6387	26.5732	26.4332	26.2222	26.7819
	Q9HW92	PA4308	17	51.2	53.246	160.8	2.36E+09	83	496	25.6636	25.4787	25.6425	25.6476	25.8	25.9172	26.5109	26.3078
g chemota	Q9HW93	pctC	12	33.2	68.308	33.836	3.26E+08	15	632	24.1451	25.2403	22.1609	25.166	23.1384	24.4878	24.457	25.0968
	Q9HWB4	PA4286	10	53.7	24.847	76.121	1.48E+09	40	227	25.7377	25.5917	25.8271	25.9057	25.7096	25.6365	25.7191	25.6804
l ligase/rep	Q9HWC0	birA	4	19.2	34.048	11.314	2.52E+08	26	312	23.7204	23.6823	23.9348	24.0091	23.7086	23.7763	23.511	22.6697
ion/antiter	Q9HWC4	nusG	13	79.7	20.162	205.92	4.89E+10	181	177	29.9971	30.135	30.1246	30.0465	30.2945	30.2195	30.1143	30.0174
osomal pro	Q9HWC5	rplK	8	61.5	14.907	82.241	1.1E+11	104	143	31.8769	31.9627	32.166	31.8904	32.0993	32.0202	32.1771	32.0388
osomal prc	Q9HWC6	rplA	20	71	24.234	268.43	3.48E+11	532	231	33.4165	33.5018	33.7253	33.5949	33.4235	33.4309	33.4303	33.3336
osomal pro	Q9HWC7	rplJ	12	50	17.634	323.31	1.42E+11	137	166	31.845	32.0916	31.9797	31.6868	32.112	32.3215	31.9834	32.2105
omal prote	Q9HWC8	rplL	9	83.6	12.479	144.88	7.56E+10	398	122	32.7144	32.153	31.8595	32.6884	32.7574	32.8086	32.9132	33.1989
A polymer	Q9HWC9	rpoC	74	66	154.38	323.31	6.85E+10	637	1399	28.2405	28.8431	27.951	28.6857	29.0963	28.8394	28.7932	29.3239
osomal pro	Q9HWD0	rpsL	6	43.1	13.799	83.016	1.1E+10	98	123	28.0356	28.8609	27.9742	28.8105	29.2403	28.8706	28.7171	29.1781
osomal prc	Q9HWD1	rpsG	13	57.1	17.504	86.438	2.75E+10	106	156	29.6075	30.1308	29.2821	30.1483	30.2381	30.2208	30.1085	30.2649
gation facto	Q9HWD2	fusA	63	83.6	77.784	323.31	1E+12	1658	706	33.0512	33.1216	33.2741	33.0466	33.1997	33.0957	33.0717	32.9817
osomal pro	Q9HWD4	rpsJ	12	56.3	11.766	98.746	1.61E+11	179	103	32.117	32.4869	32.3167	32.4109	32.2123	32.3171	32.3927	32.2089
osomal prc	Q9HWD5	rplC	13	60.7	22.591	217.21	1.52E+11	243	211	32.4037	32.5968	32.709	32.6392	32.628	32.6089	32.635	32.5314
osomal prc	Q9HWD6	rplD	13	59.5	21.64	171.34	5.45E+10	294	200	30.7229	30.9314	30.7851	30.7082	30.9934	30.9433	30.7763	30.7408
osomal pro	Q9HWD7	rplW	7	50.5	10.95	50.054	3.9E+10	70	99	30.523	31.0536	30.8352	30.7153	31.0038	30.9408	30.9138	30.8382
osomal prc	Q9HWD8	rplB	11	43.6	29.579	114.71	9.72E+10	176	273	30.6304	31.3405	30.5286	31.1746	31.5693	31.2214	31.0373	31.4215
osomal pro	Q9HWD9	rpsS	7	50.5	10.357	43.25	1.51E+10	76	91	28.8984	29.535	28.932	29.2706	29.4809	29.4821	29.5644	29.5924
osomal pro	Q9HWE0	rplV	8	42.7	11.911	221.12	8.08E+10	127	110	31.3551	31.5185	31.1833	31.8268	31.59	31.5772	31.4199	31.5232
osomal prc	Q9HWE1	rpsC	13	54.4	25.838	156.29	4.13E+10	161	228	29.3334	29.9176	28.8149	29.8314	30.5016	30.0231	29.6884	30.1244

osomal pro	Q9HWE2	rplP	6	41.6	15.401	35.096	3.49E+10	87	137	29.6306	30.2621	29.2805	30.2661	30.2178	30.1016	29.8279	30.2909
osomal pro	Q9HWE3	rpmC	5	60.3	7.2013	191.72	1.17E+11	85	63	32.4	32.0597	31.7511	32.7082	32.4799	32.7833	32.0875	32.5481
osomal pro	Q9HWE4	rpsQ	6	43.2	10.086	23.4	6.99E+10	75	88	31.5964	31.9711	31.9617	31.8891	31.8569	31.9343	32.0672	31.6616
osomal pro	Q9HWE5	rplN	11	61.5	13.412	177.65	3.56E+10	149	122	30.0158	30.4626	30.1252	30.2732	30.4324	30.1988	30.2321	30.1781
osomal pro	Q9HWE6	rplX	15	67.3	11.47	323.31	2.16E+11	270	104	32.2615	32.6388	32.3941	32.5467	32.9098	32.5163	32.8097	32.4663
osomal pro	Q9HWE7	rplE	17	63.7	20.392	127.38	1.56E+11	263	179	31.8288	32.1013	32.3037	32.273	32.0391	31.8204	31.7659	31.7556
osomal pro	Q9HWE8	rpsN	3	31.7	11.565	7.9815	9.77E+09	22	101	29.0046	29.7872	28.7391	29.343	30.2199	29.8308	29.6406	29.5402
osomal pro	Q9HWE9	rpsH	9	65.4	14.17	77.788	2E+11	382	130	32.7832	32.8557	32.6416	32.9271	32.7888	32.7598	32.7948	32.5462
osomal pro	Q9HWF0	rplF	15	73.4	19.099	117.37	2.36E+11	247	177	32.4366	32.6614	32.8596	32.5945	32.7246	32.6282	32.6161	32.4161
osomal pro	Q9HWF1	rplR	4	36.2	12.661	43.512	5.26E+10	44	116	30.5625	31.1121	30.4883	30.9727	31.3403	31.2408	31.0237	31.0952
osomal pro	Q9HWF2	rpsE	10	65.7	17.625	182.69	1.39E+11	369	166	32.3205	32.625	32.7011	32.5518	32.5927	32.4906	32.6118	32.4038
osomal pro	Q9HWF3	rpmD	2	25.9	6.4775	162.67	5.38E+10	36	58	31.6809	31.7832	31.925	31.7079	31.6493	31.8042	31.5882	31.5357
osomal pro	Q9HWF4	rplO	10	54.9	15.174	107.35	6.13E+10	89	144	30.65	31.1223	30.7171	31.0894	31.4224	31.2433	31.2417	31.1056
osomal pro	Q9HWF7	rpsM	13	65.3	13.265	184.92	3.54E+10	139	118	29.7182	30.4181	29.4601	30.2541	30.6584	30.4157	30.3445	30.6057
osomal pro	Q9HWF8	rpsK	6	49.6	13.629	67.244	7.64E+10	76	129	32.4851	32.9703	31.8785	33.2046	33.1189	33.1221	32.809	33.1019
cterioferrit	Q9HWF9	bfr	9	78.6	17.94	93.695	3.07E+09	47	154	27.5972	27.2398	26.4027	27.9066	25.9965	27.2318	26.2538	26.9096
C system pr	Q9HWG0	uvrA	26	48.9	104.49	118.08	2.41E+09	106	945	25.8687	26.0141	23.7296	26.4076	26.4786	26.2844	26.3357	26.6753
	Q9HWR2	aph	18	64.9	29.812	323.31	2.15E+10	181	268	28.7813	28.7935	28.4319	28.6401	28.7092	28.8429	29.0024	28.8729
	Q9HWV9	PA4069	6	30.3	32.939	27.225	4.3E+08	19	294	25.1682	23.4793	24.8526	23.168	25.2279	25.1931	25.0206	24.9024
	Q9HWW0	PA4068	11	57.9	33.31	99.535	3.66E+09	72	309	27.0628	26.9929	27.1224	26.9687	27.1671	27.2828	27.3673	27.2348
	Q9HWW1	oprG	4	22.8	25.193	34.431	5.54E+08	12	232	26.3588	26.5161	22.8711	22.9217	25.6594	25.6117	23.7583	25.6565
	Q9HWW5	PA4063	7	46.4	21.235	22.025	9.82E+08	31	196	26.5849	25.5159	23.0757	26.7389	25.3582	26.3511	25.3132	25.7308
	Q9HWW7	PA4061	13	47.4	31.878	210.74	1.27E+10	110	289	28.4551	28.0203	27.1324	28.5704	28.1473	28.3786	27.8344	28.2825
ional repre	Q9HWX1	nrdR	7	45.5	17.925	48.953	2.03E+09	50	154	26.2886	26.6176	26.3064	26.3655	26.3926	26.4048	26.5107	26.115
osynthesis	Q9HWX2	ribD	16	60.9	39.764	148.57	2.49E+09	89	373	26.4673	26.629	26.8699	26.3665	26.4023	26.3852	26.4321	26.432
tanone 4-p	Q9HWX4	ribB	16	66	39.438	222.66	2.35E+10	151	365	29.2569	29.4307	29.5352	29.1817	28.7144	28.9544	29.0661	28.9876
ostance prc	Q9HWX6	nusB	7	55.3	17.707	42.348	6.25E+09	51	159	28.385	28.6194	28.6618	28.6412	28.3991	28.2209	28.4007	28.2742
cyclohydrol	Q9HWY1	ribA	9	68.3	22.099	83.308	4.09E+09	67	205	26.9538	27.3252	26.9572	27.0206	27.0519	27.0907	27.0397	27.0924
	Q9HWY4	ispA	13	59	31.464	101.75	2.89E+09	90	295	26.2728	26.3266	25.7892	26.2559	26.2698	26.399	26.5265	26.3944
nuclease 7	Q9HWY5	xseB	4	75	8.9728	118.1	2.69E+09	48	80	26.8191	26.1663	25.7431	27.0082	26.8591	26.9345	26.6847	26.8406
	Q9HWZ2	PA4035	11	46.8	35.895	80.897	1.47E+09	51	333	26.2572	26.2331	26.0776	26.3322	25.8502	25.8019	25.7453	25.9321
	Q9HWZ5	PA4032	2	25.6	27.422	23.714	5.9E+08	15	238	25.5183	23.7051	23.1445	25.3182	25.2833	25.4906	25.9807	26.04
c pyrophos	Q9HWZ6	ppa	13	60	19.396	151.9	7.14E+10	194	175	31.4744	30.5477	29.8871	31.3076	30.8416	31.3232	30.3966	31.2517
	Q9HX07	mpl	10	36.8	48.469	123.03	5.72E+09	87	451	27.4448	27.4059	27.3544	27.436	27.8265	27.689	28.108	27.7226
	Q9HX10	PA4017	11	52.1	23.164	51.858	2.98E+09	77	213	26.7316	26.8539	26.5401	26.717	26.5772	26.466	26.6559	26.4129
myl phosph	Q9HX20	proA	23	74.1	45.044	323.31	2.18E+10	181	421	28.2735	28.2515	28.4482	28.2237	28.2776	28.4576	28.6255	28.4468
nucleotide	Q9HX21	nadD	6	44.4	23.801	67.995	1.12E+09	30	214	25.4142	25.8763	25.8291	25.7769	25.7576	26.1076	26.2425	25.7018
l silencing f	Q9HX22	rsfS	7	83.9	13.054	81.313	6.09E+09	63	118	28.2283	27.8407	27.7203	28.3748	27.3995	28.0206	27.9819	28.1418
e subunit r	Q9HX23	rlmH	6	50.3	17.79	39.477	7.37E+08	24	155	25.1267	25.0753	25.4298	25.1807	25.4507	25.4122	25.5323	25.2367
	Q9HX24	sltB1	16	65	37.867	134.22	3.44E+09	84	340	26.4907	26.5388	26.8964	26.7106	26.9912	26.798	26.8153	26.3701
oyl syntha	Q9HX25	lipA	19	63.3	36.733	187.19	1.13E+10	158	327	27.9282	27.916	28.0266	27.8598	28.0079	28.0618	28.2336	28.0653
	Q9HX28	PA3992	13	47.8	47.76	85.448	1.06E+09	46	448	24.892	24.9736	25.1394	24.8167	24.8492	24.7959	24.834	24.8256
	Q9HX31	holA	8	43.2	37.378	47.155	9.72E+08	50	345	24.5869	24.4209	24.3685	24.7239	24.8311	24.8293	24.9478	24.9525
ine--tRNA l	Q9HX33	leuS	42	59.7	97.647	323.31	2.42E+10	330	873	27.6941	27.7628	27.2875	28.0009	28.4847	28.4122	28.134	28.0121
	Q9HX36	PA3983	12	47.3	31.548	74.32	3.49E+09	90	279	26.3786	26.4862	26.77	26.2303	26.6798	26.6688	26.292	26.2681
ibonucleas	Q9HX37	ybeY	6	49.4	17.981	50.99	7.75E+08	21	160	25.9972	25.1673	22.6203	25.7726	24.9842	25.398	25.3324	25.3741
	Q9HX38	PA3981	11	50.6	38.424	62.953	1.95E+09	47	340	26.068	26.4617	26.6345	25.9547	26.0807	26.1398	26.3928	26.8522
	Q9HX39	PA3979	4	46.4	12.624	26.519	1.35E+09	26	110	26.1081	25.9665	25.6172	26.0211	26.2543	26.4801	25.896	26.2328
-phosphate	Q9HX40	thiE	8	64.6	22.146	135.55	1.68E+09	37	209	26.6701	26.3118	27.0948	26.9459	26.6929	26.7974	26.9067	26.6818
	Q9HX41	thiD	9	56.6	27.979	127.73	3.7E+09	76	265	27.1305	26.8827	26.6992	26.975	26.8147	27.0802	26.8397	27.0666
	Q9HX44	PA3972	18	55.9	60.338	127.58	1.92E+09	76	549	25.7743	26.1357	25.8017	26.0046	25.4601	25.4336	25.3743	25.4573
P nucleosic	Q9HX46	amn	9	29.7	56.225	50.431	5.94E+08	32	499	24.0858	24.8733	24.3578	24.448	24.368	24.228	24.3506	24.5871
	Q9HX49	PA3967	4	51.5	14.705	31.339	1.99E+09	22	130	27.4261	27.3252	27.3583	27.5926	27.5311	27.2527	27.3931	27.1539
	Q9HX51	PA3965	8	48.5	19.289	23.345	1.43E+09	29	169	27.1349	27.3533	27.6802	26.9363	26.8053	27.1354	26.7473	26.5092
	Q9HX64	PA3952	4	42.9	19.793	102.88	2.53E+09	44	191	26.6822	25.8747	24.2875	26.4039	26.8212	27.5078	26.7439	27.3427
	Q9HX66	PA3950	5	15.4	48.6	16.871	1.51E+08	16	449	23.5343	23.906	22.1072	23.7021	23.939	23.8188	23.3261	23.7622
	Q9HX68	rocA1	6	34.9	22.995	24.912	8.59E+08	25	209	25.7395	25.71	26.1043	25.7115	25.7487	25.6061	25.6917	25.4196
	Q9HX71	PA3945	3	21.9	22.403	10.961	3.18E+08	21	196	24.6232	24.6404	24.6491	24.5732	24.5259	24.5639	25.2736	24.15
	Q9HX72	PA3944	4	28.6	21.857	27.772	6.54E+08	24	192	25.0842	24.9306	24.9191	25.0258	25.1035	25.0812	25.1701	25.0923
	Q9HX74	tesB	7	40.1	32.934	56.143	1.76E+09	58	289	26.4714	26.5126	26.3437	26.4352	26.718	26.2827	26.1694	26.1679
	Q9HX85	PA3931	15	54.4	28.101	103.19	1.16E+10	109	259	28.2736	28.7439	27.4263	28.5195	28.6175	28.2969	28.1365	28.5416
phosphate s	Q9HX95	moaC	6	41.2	17.338	32.286	4.46E+09	51	160	27.3395	27.3388	26.7953	27.5252	26.9281	27.0028	26.8993	26.9838
nthase sulf	Q9HX96	moaD	3	43.4	9.2153	34.751	1.61E+09	32	83	27.3493	26.7591	26.6842	27.7043	26.58	27.4082	26.9099	26.6494
synthase c	Q9HX97	moaE	5	58	16.686	26.829	9.65E+08	28	150	26.3439	25.6604	25.4852	26.2006	25.4669	24.9007	25.2842	25.634
actor biosy	Q9HX98	moaB1	11	88.6	19.669	98.495	2.11E+09	63	185	26.8987	26.7195	27.0412	26.8219	26.6444	26.9131	26.5292	26.7433
hain releas	Q9HXB0	prfC	22	49.1	59.865	184.32	4.94E+09	127	527	27.0864	27.0976	26.9473	26.7474	26.8861	27.1932	27.0154	27.3296
	Q9HXB7	PA3896	12	54.5	35.632	64.064	1.32E+09	36	325	25.6192	25.7117	25.7735	25.3951	25.2617	25.5282	25.8034	25.7859
ic D-argini	Q9HXE3	dauA	8	45.1	40.566	26.253	1.26E+09	15	375	23.1284	23.9254	24.0622	22.7182	24.4356	23.7846	24.0551	23.7812
bolic L-argi	Q9HXE4	dauB	9	54	33.945	70.892	1.3E+09	57	315	25.7614	25.7546	25.8388	25.5025	24.8101	25.3363	25.2539	25.3032
lent RNA h	Q9HXE5	rhlB	15	56.9	44.287	122.13	3.14E+09	96	397	26.1659	26.2659	25.9998	26.1814	26.399	26.4872	26.2641	26.3157
	Q9HXE7	PA3859	8	78.1	23.42	151.86	1.19E+10	104	215	28.2839	28.1007	27.8775	28.1314	27.6036	28.0259	27.8763	28.1206
	Q9HXF6	PA3850	4	28.1	33.613	50.178	8.8E+08	21	303	25.8293	25.3791	22.5284	26.0078	25.5525	26.2495	25.5892	25.8539
ociated prc	Q9HXF7	PA3849	14	50.9	37.93	162.02	5.95E+09	132	334	26.6652	26.7745	27.0201	26.616	26.7217	26.7641	26.9365	26.767
	Q9HXG2	PA3844	3	22.2	22.795	13.796	3.2E+08	18	203	24.945	25.0285	25.1829	24.8692	24.975	22.472	24.8624	24.1395
	Q9HXG6	PA3838	12	56.8	28.444	34.549	7.74E+08	28	264	25.1759	25.6585	25.807	25.3409	24.9827	25.2195	25.2142	25.3888
	Q9HXH1	PA3833	4	68	13.511	56.351	7.88E+08	22	122	25.972	23.0735	22.7741	26.0726	23.3442	25.6032	23.7949	25.9577
tRNA ribos	Q9HXH8	queA	9	42.9	38.16	76.255	2.75E+09	60	347	26.6207	26.5664	26.6355	26.5195	26.5839	26.8987	26.6775	26.8119
NA-ribosyl	Q9HXH9	tgt	25	86.8	41.205	280.83	1.51E+10	217	372	27.668	27.7694	27.7652	27.6693	28.1005	28.1114	28.154	27.9093
nslocase su	Q9HXI1	secD	19	36.6	67.674	76.894	1.32E+09	51	620	24.414	25.0972	24.4471	25.0031	25.7934	25.0186	24.9351	25.3287
nslocase su	Q9HXI2	secF	3	12.4	33.041	35.919	4.17E+08	12	306	23.4407	24.458	22.5534	24.4972	25.2493	22.7548	24.7097	24.9246
-monopho	Q9HXI4	suhB	27	54.2	29.521	259.86	1.43E+10	200	271	27.9948	28.0664	27.6	28.0436	27.8406	27.998	27.5877	27.7526
e-2'-O-)-me	Q9HXI5	trmJ	9	49	28.511	81.761	3.44E+09	80	257	27.0052	26.8338	27.0007	27.0874	26.9316	26.8477	26.6955	26.5548
	Q9HXI6	cysE	6	40.7	27.89	28.511	2.58E+08	14	258	23.7619	24.2774	22.7721	23.9555	21.6863	23.6602	23.7958	24.5173
	Q9HXI7	iscR	4	44.2	17.853	44.19	1.09E+09	31	163	25.9382	25.8361	26.0492	26.0098	26.3281	25.863	25.95	26.108
e desulfur	Q9HXI8	iscS	23	59.2	44.666	311.64	4.25E+10	249	404	29.8208	30.0239	30.1388	29.6479	29.7804	29.8627	29.7066	29.7287
	Q9HXI9	iscU	10	75	13.753	158.63	2.07E+10	107	128	29.4823	29.1112	28.3385	29.5443	29.044	29.4888	28.7892	29.3794
e protein H	Q9HXJ1	hscB	5	54.3	20.235	35.996	2.1E+09	27	173	27.1492	27.2859	26.5288	27.2481	27.6624	27.4217	24.0072	27.1803
	Q9HXJ3	PA3804	4	14.7	36.102	15.429	2.51E+08	17	347	23.5558	24.5462	22.642	23.6061	24.3922	24.6129	24.4866	24.7012
-1-yl dipho	Q9HXJ4	ispG	27	83.6	40.056	308.29	1.92E+10	234	371	28.1301	28.1992	28.423	28.2061	28.2681	28.2763	28.3262	28.2722
	Q9HXJ6	PA3801	7	52.8	23.14	56.906	2E+09	46	214	26.0834	26.3907	24.841	26.4848	26.985	26.6494	26.4509	27.128
GTPase De	Q9HXJ8	der	16	48.3	55.005	82.249	2.99E+09	102	493	26.053	26.4719	25.6804	26.377	26.6291	26.3969	26.1987	26.8007
	Q9HXJ9	PA3798	11	46.1	42.594	72.323	1.24E+09	60	382	24.9871	24.6925	24.8158	24.5413	24.9098	25.0549	25.3091	25.1752
	Q9HXK0	PA3797	11	54.2	29.996	100.4	1.69E+09	76	264	26.2767	25.6888	25.1404	26.2741	25.7975	26.2431	25.4243	26.011
	Q9HXK4	PA3793	3	46.4	12.459	47.517	1.72E+09	22	110	26.7601	26.6431	22.3557	26.7733	26.7478	26.7601	26.8961	26.732
pylmalate s	Q9HXK5	leuA	33	68.3	61.726	323.31	8.3E+09	203	556	27.8227	27.7713	27.8723	27.5876	27.4209	27.5206	27.4751	27.4801
	Q9HXK8	PA3787	8	46.8	30.773	38.862	1.62E+09	43	282	25.5487	25.9456	25.8949	25.849	26.1107	26.0742	25.982	25.8641
	Q9HXL0	PA3785	7	43.7	17	43.656	1.3E+10	67	158	29.414	28.1562	27.3745	29.5528	27.3812	28.9423	27.4875	28.7571
	Q9HXL6	PA3779	7	27.5	37.696	36.353	4.22E+08	23	346	24.8597	24.489	24.1237	24.8323	24.5044	24.5929	24.4079	24.7445
e [glutamin	Q9HXM6	guaA	31	76	57.959	269.66	1.52E+10	217	525	27.4355	27.6825	26.8644	27.8097	27.8291	27.6861	27.3847	27.7045
c adenosin	Q9HXM8	tadA	5	51.1	19.938	31.741	5.79E+08	20	182	25.0129	24.9077	24.8794	23.5068	25.4309	25.1099	25.6544	24.946
	Q9HXN0	PA3765	7	51.5	20.903	27.301	6.85E+08	33	194	25.1818	25.2667	24.5847	25.2674	25.2456	25.3852	25.3068	25.3985
ormylglycin	Q9HXN2	purL	50	58.4	140.65	323.31	1.27E+10	298	1298	26.9093	27.3407	26.3418	27.3649	27.6083	27.4168	27.2642	27.6535
	Q9HXN3	PA3762	4	65.7	11.702	35.266	2.32E+09	19	105	27.754	25.1381	25.2876	26.969	25.6881	28.4744	25.977	26.9686
	Q9HXN6	PA3759	9	44.7	36.123	42.798	3.54E+08	31	340	24.1742	23.983	24.4466	23.9668	23.9776	24.1409	24.0753	24.099
ine-6-phos	Q9HXN7	PA3758	9	49	38.83	64.947	8.06E+08	44	363	24.6917	24.7254	23.2197	24.7326	24.8961	24.7833	24.1514	24.737
	Q9HXP1	PA3754	2	17.7	22.855	11.587	3.19E+08	12	203	24.69	24.4068	24.8688	22.5671	24.307	24.2568	24.5042	24.2212
cinamide fo	Q9HXP3	purT	21	65.1	42.342	317.11	2.19E+10	208	393	28.864	28.9383	29.108	28.7389	28.6123	28.665	28.7	28.7244
gnition part	Q9HXP8	ffh	22	58.2	49.359	127.21	3.9E+09	104	457	25.9003	26.2294	25.9002	26.0599	26.359	26.3778	26.4004	26.3874
osomal pro	Q9HXP9	rpsP	4	49.4	9.2043	131.48	8.14E+10	79	83	32.0657	32.1674	32.0422	32.1571	31.7076	31.9345	32.1414	32.0358
naturation f	Q9HXQ0	rimM	6	42.9	19.789	49.674	2.53E+09	29	175	27.2803	27.2626	27.1087	27.5311	27.3543	27.459	27.338	27.6368
-N(1)-)-met	Q9HXQ1	trmD	12	51.6	28.359	67.075	2.87E+09	87	252	26.0806	26.185	25.1266	26.2156	26.4566	26.2422	26.3633	26.1424
osomal pro	Q9HXQ2	rplS	9	56.9	13.032	76.513	6.83E+10	153	116	30.611	31.0084	30.4516	31.0412	31.4526	31.1561	31.0815	31.2062
	Q9HXQ9	PA3733	7	31.8	44.766	37.704	7E+08	31	409	25.1525	25.0629	25.5053	25.5663	25.4196	24.8851	25.7102	25.3164
	Q9HXR0	PA3732	2	19	15.959	38.653	1.56E+09	24	147	26.2166	26.2122	25.9189	26.548	26.74	26.6342	26.6818	26.4892
	Q9HXR1	PA3731	14	61.5	24.893	138.97	8.32E+09	111	231	27.3509	27.3957	26.2686	27.4294	27.5782	27.4196	27.6604	27.3393
	Q9HXR6	PA3726	5	35.8	20.596	63.466	3.45E+09	40	179	26.9898	27.3433	27.2199	27.118	27.4118	27.1535	27.1218	26.8586
olyl cis-tran	Q9HXS4	PA3717	5	56.6	12.644	43.549	1.49E+09	30	113	26.3342	26.5122	25.7308	26.3409	26.5237	26.491	26.6339	26.4665
	Q9HXT7	wspE	8	22.8	84.324	26.16	3.2E+08	24	769	24.4849	25.0106	24.6401	22.0093	22.3548	24.9112	24.4971	23.6641
	Q9HXT9	wspR	12	49.3	37.887	66.997	1.58E+09	52	347	25.5426	25.4795	25.7265	25.4718	25.4547	25.4104	25.461	25.3533
ne--tRNA li	Q9HXU0	lysS	38	72.5	57.328	323.31	4.56E+10	363	501	28.4277	28.543	28.5958	28.2082	28.6096	28.7233	28.8167	28.9117
	Q9HXU1	PA3699	4	27.8	26.345	28.33	8.11E+08	35	237	25.201	25.3553	25.5544	24.9143	25.0004	24.8893	24.6227	24.7309
	Q9HXU4	PA3696	6	32.3	26.551	22.857	4.69E+08	27	248	24.9021	25.2003	25.5748	24.6136	24.8477	25.1734	24.8163	23.1707
containing	Q9HXU7	PA3693	3	32.4	18.219	18.857	5.7E+08	20	173	25.0945	25.419	25.0608	25.5114	25.251	25.6234	25.2081	25.4096
	Q9HXV1	PA3689	4	39.7	17.694	30.685	6.65E+08	27	156	25.8114	25.6755	23.6311	25.6142	25.5483	25.2681	25.3087	25.4501

enylate kin	Q9HXV4	adk	12	65.1	23.107	238.3	5.75E+10	219	215	30.6777	30.5848	29.6854	30.979	30.6473	30.7243	30.3047	30.4881
	Q9HXV5	PA3685	5	38.5	24.18	61.636	1E+09	35	226	25.8892	25.6339	25.9545	26.1415	25.5274	25.8761	26.0338	25.8946
all subunit r	Q9HXW0	rsmJ	6	34.5	27.904	27.186	4.18E+08	13	261	25.117	24.6756	24.0591	25.3128	25.0197	25.0394	24.8346	25.3199
	Q9HXW2	PA3678	7	52.8	23.15	30.699	5.04E+08	28	212	24.5094	23.9977	23.3718	24.6188	24.6044	24.6549	24.6396	24.3133
	Q9HXW6	PA3674	4	47	14.393	30.204	9.18E+08	14	132	23.5942	22.9637	23.1853	25.3798	23.1023	26.8055	27.2072	26.8777
	Q9HXX2	PA3668	6	62.1	15.927	57.786	6.63E+08	36	140	25.0465	24.9157	24.6091	25.1283	24.9855	24.8491	24.8385	24.6759
cysteine de	Q9HXX3	csd	10	37.9	43.274	74.441	1.34E+09	56	401	25.107	25.3023	25.5021	25.1943	25.4073	25.4663	25.4368	25.2106
	Q9HXX5	PA3664	9	77.4	13.111	52.324	4.85E+09	58	115	27.7784	27.4028	27.0728	27.895	27.4826	27.6259	27.3375	27.6016
ine aminop	Q9HXY1	map	15	75.1	29.086	154.82	2.74E+10	139	261	29.4073	29.3934	29.374	29.3121	29.1173	29.3451	29.3623	29.2788
te synthase	Q9HXY2	uppS	4	22.7	28.04	104.48	1.67E+09	24	251	26.4618	27.2405	27.4249	26.9554	26.9977	27.2964	27.7496	27.2733
p-like prote	Q9HXY5	PA3647	7	38.7	19.09	46.408	4.7E+10	87	168	30.4054	30.6863	30.3372	30.5898	30.9268	30.8585	30.9813	30.8318
cosamine N	Q9HXY6	lpxD	7	31.7	36.191	40.024	1.84E+09	31	353	26.0875	26.136	26.15	26.0611	26.093	26.3131	26.002	26.186
saccharide	Q9HXY8	lpxB	8	32	41.294	30.549	2.8E+08	24	378	24.0408	23.5937	23.1583	24.1998	23.4422	23.9927	23.6806	24.3556
onuclease	Q9HXY9	rnhB	9	49.3	21.771	28.041	8.36E+08	26	201	25.9436	22.9005	23.3581	25.4773	24.3265	25.5367	25.2662	25.9707
erase III su	Q9HXZ1	dnaE	16	21.7	130.9	47.363	3.26E+08	36	1173	23.5179	23.2202	23.2697	23.3847	24.4264	23.3062	23.5092	24.4633
ase carbox	Q9HXZ2	accA	21	66.8	34.947	323.31	4.21E+10	231	316	29.4423	29.5416	29.4317	29.3693	29.1913	29.3788	29.4393	29.4718
TP synthas	Q9HXZ4	pyrG	32	75.3	59.617	323.31	2.04E+10	252	542	27.7137	28.0049	27.7055	27.7294	28.1425	28.2833	28.179	28.561
Enolase	Q9HXZ5	eno	22	63.4	45.195	323.31	1.59E+11	356	429	30.9481	30.9338	31.0093	31.0262	31.1725	31.2794	31.3605	31.137
	Q9HXZ8	PA3632	5	79.3	9.1152	30.86	1.54E+09	25	82	27.1709	25.6926	24.7867	27.1374	25.1411	26.7953	25.0888	26.2982
)glutathior	Q9HY01	adhC	12	46.5	39.208	123.22	2.08E+10	127	370	29.2038	29.1518	29.4209	28.973	29.377	29.5182	29.4924	29.5118
	Q9HY02	PA3628	11	60.4	31.275	133.6	6.87E+09	100	283	27.5861	27.4723	27.6101	27.7596	28.2714	27.9687	27.9524	27.6366
icleotidase	Q9HY05	surE	6	37.8	26.368	70.64	3.2E+09	56	249	26.7647	26.7265	26.4861	26.768	26.8322	26.7783	26.7008	26.747
erredoxin	Q9HY07	fdxA	3	24.3	12.159	22.31	1.03E+10	39	107	29.2362	28.7599	26.9895	29.4381	28.7675	29.2052	27.7236	29.0797
tch repair p	Q9HY08	mutS	18	32.6	94.977	80.082	4.81E+08	55	855	23.2415	24.0042	23.746	24.2214	25.118	24.2005	23.8422	24.8299
	Q9HY10	PA3618	6	59.5	17.715	51.348	6.28E+08	27	168	25.1055	25.6185	25.4103	25.1021	25.8254	25.5337	25.5381	25.3713
	Q9HY11	PA3615	7	26.1	40.465	28.288	5.05E+08	25	356	25.3581	25.4844	25.0918	25.3474	24.9859	24.2962	24.646	22.8392
	Q9HY15	PA3611	3	37.5	14.986	82.416	1.45E+10	19	136	30.4683	29.9858	28.6254	30.2675	30.0258	30.3328	29.9942	30.3544
import ATP	Q9HY19	potA2	8	43.8	40.044	58.643	9.64E+08	32	363	26.4848	25.8003	26.2404	26.3382	25.7575	26.0505	25.3836	25.7668
	Q9HY22	erdR	10	69.1	23.531	98.525	6.66E+09	91	217	27.6237	27.7335	27.3849	27.6116	27.5466	27.4916	27.5307	27.5446
	Q9HY50	PA3567	13	64.7	36.677	157.65	8.03E+09	77	337	28.2614	28.5552	28.7506	28.2168	28.2681	28.2838	28.386	28.2308
	Q9HY51	PA3566	4	68	11.167	28.248	2.47E+09	17	97	28.4898	24.1753	26.3927	28.0512	22.361	28.4957	27.5699	28.6189
	Q9HY54	fruR	11	39.5	36.387	76.712	6.35E+08	51	329	24.6189	24.8823	25.0605	24.4069	24.4425	24.4874	24.5526	24.564
	Q9HY55	fruI	16	32.5	101.57	107.48	1.27E+09	71	956	25.094	24.8951	24.9683	24.9203	24.6682	25.2857	24.9331	25.2029
phofructok	Q9HY56	fruK	7	33.8	33.381	40.382	1.25E+09	42	314	26.2781	26.1863	26.8075	26.2509	25.5422	25.9338	26.0176	25.8335
	Q9HY58	PA3559	19	50.2	51.795	169.11	6.52E+09	139	464	26.8478	26.9076	27.2715	26.3449	26.9537	27.3541	27.4096	27.6398
oxy-4-form	Q9HY64	arnC	9	39.8	37.296	22.454	6.07E+08	30	339	23.7872	24.4013	23.4363	24.2319	24.6442	24.7746	24.0616	24.9339
binose--oxo	Q9HY65	arnB	20	60.2	41.866	248.54	4.85E+10	205	382	30.0581	30.1026	30.189	30.1305	30.7252	30.5273	30.5827	30.3687
46 protein I	Q9HY72	PA3539	11	62.9	29.56	110.61	3.17E+09	59	259	26.8391	26.9431	27.0908	26.3888	26.8304	27.0151	27.2039	26.8617
	Q9HY73	PA3538	9	46.7	39.189	48.229	1.15E+09	31	360	25.7553	26.2624	25.8637	25.6024	25.9666	26.1859	26.194	26.1312
Glutaredoxi	Q9HY77	PA3533	7	61.1	11.843	31.402	1.25E+10	50	108	30.7425	29.0689	28.5093	30.3806	28.2746	30.0469	28.1747	29.1308
	Q9HY81	PA3529	14	89.5	21.822	165.43	1.29E+11	198	200	31.8066	32.1234	32.0253	31.8769	31.8999	31.946	32.1036	31.8926
bonuclease	Q9HY82	rnt	10	62.9	24.723	148.34	6.3E+09	74	224	27.5186	27.5253	27.417	27.4153	27.2005	27.2426	27.306	27.1973
osuccinate s	Q9HY84	argG	30	82.7	45.297	323.31	3.11E+10	259	405	29.4262	29.6438	29.674	29.1864	29.0504	29.3793	29.2686	29.2836
	Q9HYB3	PA3496	5	79.7	6.8834	22.389	1.94E+09	27	59	27.8232	27.4476	24.6614	28.1568	27.8993	27.5125	27.0432	27.6497
donuclease	Q9HYB4	nth	8	42.9	23.737	40.025	9.16E+08	34	212	25.8207	26.0677	26.0213	25.9496	26.0882	26.089	25.8083	25.9534
onine--tRNA	Q9HYC7	metG	32	65.7	74.903	323.31	1.61E+10	219	677	27.2338	27.3767	26.9709	27.4754	27.8919	27.7976	27.522	27.5574
	Q9HYC8	PA3481	12	54.4	38.888	104.77	5.48E+09	69	364	27.697	27.7421	27.7286	27.3678	27.5138	27.6816	27.9229	27.9586
triphospha	Q9HYC9	dcd	6	46.3	21.154	69.637	3.76E+09	53	188	27.21	27.3432	27.5362	27.3148	27.3281	27.3783	27.3822	27.3087
endent mal	Q9HYD5	maeA	22	64.4	62.42	323.31	1.14E+10	142	564	27.8737	27.7087	27.7079	27.7016	28.0107	28.3515	28.519	28.3335
	Q9HYE0	PA3466	13	43.5	48.989	56.076	1.21E+09	72	446	24.761	25.0326	24.5472	25.159	24.9764	24.8506	25.0124	25.2991
02 protein I	Q9HYF3	PA3453	8	57.5	24.16	82.422	4.15E+09	59	221	26.6572	26.8094	26.9337	26.6039	27.1695	26.9461	27.0572	26.8181
:quinone o	Q9HYF4	mqo1	11	32.1	57.21	53.234	1.36E+09	56	523	25.0805	25.4162	25.0513	25.5946	25.63	25.2667	25.0705	25.5787
	Q9HYF6	PA3450	13	73.1	24.164	115.29	9.74E+09	93	212	28.0901	29.0376	27.9351	28.2022	28.2386	27.8805	28.123	28.0756
	Q9HYG6	PA3440	7	63.1	11.637	104.31	8.18E+09	60	103	29.0489	27.1092	26.4176	28.9626	27.2109	29.0309	27.0091	28.3543
yclohydrola	Q9HYG8	folE1	9	53.8	20.833	22.816	4.64E+08	27	186	24.2958	24.5998	24.3496	22.692	24.3854	24.5422	24.3636	24.2224
	Q9HYG9	folM	5	29.1	25.403	29.582	3.7E+08	23	234	24.2432	24.6373	24.8	24.4934	24.1099	24.2692	24.3134	24.1108
erized prote	Q9HYH1	PA3435	6	70	15.871	66.043	6.12E+09	61	150	28.6617	27.9152	27.3978	28.658	27.7833	28.4919	28.0044	28.3617
	Q9HYH9	PA3426	4	25	26.998	11.506	1.85E+08	14	256	24.0308	24.2718	24.1223	24.2915	22.6599	23.4108	23.8726	24.3081
	Q9HYI4	PA3421	2	7.7	50.69	10.165	83959000	8	456	23.4444	23.6191	24.5145	23.2346	22.991	23.1662	23.482	23.6834
	Q9HYJ2	PA3413	3	56.8	10.157	16.412	1.83E+09	29	88	27.4788	26.6784	25.2006	27.5299	27.1615	27.8336	26.1041	27.3677
D-serine de	Q9HYN9	dsdA	13	49.1	48.218	77.682	2.31E+09	94	448	26.1563	26.3227	26.364	25.9845	26.2713	26.4101	26.5733	26.835
	Q9HYP0	PA3356	10	39.2	45.161	163.82	1.63E+09	60	413	25.2837	25.3502	25.3478	25.0907	24.9619	25.2554	24.917	25.0762
	Q9HYP3	PA3353	14	53.6	29.884	60.728	1.47E+09	66	263	25.3219	25.858	26.026	25.4179	25.1344	25.2012	25.1387	24.8414
	Q9HYP4	PA3352	8	51.9	17.255	55.251	2.13E+09	46	156	26.9339	26.4071	25.6661	26.9349	26.4111	26.4676	25.998	26.649
	Q9HYP5	flgM	7	78.5	11.25	100.89	1.74E+09	55	107	26.3773	24.6831	24.9745	26.1995	23.8943	25.6003	24.6108	25.3544
	Q9HYP6	PA3350	5	36.2	25.522	65.255	6.05E+08	28	232	24.8142	25.4315	25.2443	25.1796	25.1395	23.0739	25.1623	24.9525
	Q9HYQ4	PA3341	8	63.9	16.071	100.71	3.65E+09	58	144	27.0694	27.1166	27.539	27.4846	27.537	26.9431	27.2406	26.9982
protease p	Q9HYR9	clpP2	4	41.3	22.142	23.26	2.79E+08	16	201	24.0481	24.3254	22.5157	24.1758	23.7137	23.925	24.5001	23.7438
	Q9HYS3	PA3322	6	34.5	26.023	17.548	3.55E+08	33	232	23.929	23.6975	23.1246	24.2367	24.0576	23.819	23.4963	23.97
	Q9HYT1	PA3313	20	61.8	36.539	159.2	1.88E+10	176	335	28.1535	28.5309	28.2763	28.0657	28.0527	28.4624	28.5014	28.3235
e-associate	Q9HYT6	rapA	21	36.1	106.87	109.57	2.2E+09	79	950	25.3483	25.6669	23.9345	25.4884	25.7864	25.3218	25.482	26.1842
	Q9HYU4	fadD1	20	56.4	61.654	176.01	9.8E+09	168	562	27.2351	27.4778	26.7972	27.3978	27.883	27.7105	27.3588	27.466
	Q9HYU8	PA3295	5	51.7	16.165	72.351	4.48E+09	51	145	28.1466	27.3911	26.9166	28.2996	27.4982	28.2627	27.3757	27.7681
[acyl-carrie	Q9HYV7	PA3286	19	69.4	38.171	276.13	3.01E+10	172	350	29.7324	29.7329	28.8038	29.6234	30.1331	29.8937	29.6571	29.7889
	Q9HYX1	PA3270	7	55.4	21.901	51.01	1.36E+09	49	195	25.242	25.1194	24.9835	25.6338	25.8653	25.8342	25.599	25.2441
n-associate	Q9HYX7	rdgC	21	68.3	34.015	153.06	1.32E+10	145	306	27.5202	27.5873	27.8496	27.4114	27.6267	27.6165	27.9755	27.8308
	Q9HYY0	PA3260	5	45.6	10.926	19.058	1.15E+09	25	103	26.7984	25.643	25.3258	26.8869	25.4454	26.063	25.5948	25.9938
	Q9HYY3	prc	35	57.9	78.182	205.45	1.62E+10	225	698	27.9225	28.0897	28.3314	27.9921	28.0389	28.0344	28.088	27.7891
	Q9HYY4	PA3256	12	44.4	33.419	69.237	9.67E+08	62	320	24.4223	24.388	24.4727	24.0649	24.1351	24.3938	24.5066	24.4427
	Q9HYY5	PA3255	8	64.6	21.635	38.594	2.51E+09	53	192	26.9889	27.3501	27.232	26.8219	27.1134	26.9676	27.0529	27.1833
	Q9HYZ4	rluA	8	53.6	24.338	39.584	2.17E+09	39	211	26.8605	27.0115	27.1589	27.0461	27.419	27.2856	27.3398	27.0243
pological sp	Q9HYZ5	minE	6	42.9	9.7901	11.909	2.38E+09	38	84	26.5384	26.4206	26.5037	26.5751	26.9958	26.9185	27.0705	26.2949
termining p	Q9HYZ6	minD	20	72	29.635	293.21	4.3E+10	216	271	29.6983	29.8762	30.1394	29.6188	30.0871	29.9449	30.1775	29.7737
ite-determ	Q9HYZ7	minC	8	49.8	28.124	119.57	3.37E+09	63	263	27.393	27.1905	27.1137	27.1706	27.4633	27.5099	27.3103	27.2327
	Q9HZ02	PA3238	7	26.7	50.985	24.014	3.13E+08	23	453	23.6396	24.3406	24.0467	23.7831	24.4138	23.9832	24.6121	24.3299
	Q9HZ32	PA3208	7	65.6	19.948	79.777	2.79E+09	51	186	26.7277	26.7132	26.0182	26.9258	26.3182	26.7341	26.3074	26.6151
	Q9HZ36	PA3204	9	52	24.849	70.601	9.21E+08	42	225	25.4808	25.4959	25.1645	25.4037	25.5347	25.5689	25.5221	25.3396
	Q9HZ38	PA3202	4	23.2	10.609	8.6764	2.35E+09	37	99	27.2937	25.9183	25.5865	27.3221	26.0307	27.4058	26.1157	27.0079
	Q9HZ40	PA3200	5	41.7	32.215	28.087	4.26E+08	15	295	22.5395	25.5128	22.3731	23.7609	25.1614	25.077	24.1823	25.2542
	Q9HZ41	PA3199	5	29.7	23.143	15.088	2.56E+08	17	209	24.0593	23.3955	24.1451	24.6285	23.9275	24.3594	24.3292	24.284
d condensa	Q9HZ43	PA3197	9	43.7	36.905	70.152	7.75E+08	35	332	22.6361	25.061	25.4933	24.6341	25.0144	25.2474	25.5567	25.4926
	Q9HZ44	PA3196	5	18.8	19.293	19.486	4.9E+08	21	176	25.253	24.9153	25.027	25.727	24.9272	25.6469	23.177	25.4555
unit pseud	Q9HZ55	rluB	15	49.7	43.724	81.816	2.79E+09	86	386	25.9549	26.7966	26.1405	26.4991	26.6726	26.6323	26.0686	26.6053
	Q9HZ61	PA3173	8	39.4	26.332	127.52	1.98E+09	52	246	25.6993	26.0531	25.8096	25.7041	25.8628	26.1894	25.8355	25.9323
ycolate pho	Q9HZ62	gph2	11	71.2	24.976	87.93	3.63E+09	85	226	26.5556	26.5438	26.1059	26.5171	26.4359	26.5708	26.5182	26.5912
nthesis O-r	Q9HZ63	ubiG	14	76.3	25.859	115.43	7.03E+09	133	232	27.2351	27.3834	27.4075	27.1675	27.1726	27.3242	27.2703	27.2717
se-1-phosp	Q9HZ65	mtnA	19	79.1	39.425	176.53	1.01E+10	115	358	27.8083	27.9814	27.9574	27.7075	27.7768	27.8576	27.808	27.7813
rine amino	Q9HZ66	serC	17	57.9	39.949	96.063	7.9E+09	117	361	26.9029	27.092	26.9446	26.6814	27.0712	27.2851	27.3333	27.3295
protein;Pr	Q9HZ67	pheA	16	56.2	40.632	122.91	5.55E+09	121	365	26.722	26.8248	26.7031	26.4602	26.8006	27.115	27.0844	27.0638
phate amir	Q9HZ68	hisC2	21	76.4	39.482	196.27	7.28E+09	137	369	26.7766	26.7615	26.9076	26.5039	27.0432	27.2781	27.3876	27.4311
idylate kina	Q9HZ70	cmk	13	67.2	24.637	138.44	5.44E+09	65	229	27.4378	27.4386	27.3375	27.5849	27.4917	27.7331	27.4758	27.3868
osomal pro	Q9HZ71	rpsA	38	68.5	61.869	323.31	9.15E+11	1141	559	33.6591	33.7014	33.8895	33.7284	33.8805	33.7173	33.8368	33.7053
	Q9HZ93	PA3131	7	49.8	22.852	51.638	9.9E+08	34	215	25.4675	25.1726	25.1123	25.5642	25.5176	25.6164	25.5417	25.3552
	Q9HZ94	PA3130	5	47.6	16.555	58.278	1.49E+09	34	145	26.1646	26.1693	26.1417	25.9336	26.3003	26.354	26.3165	25.9229
	Q9HZ96	PA3128	8	41.5	26.219	27.192	2.45E+08	12	248	24.2582	24.3218	24.5255	22.5693	24.2312	23.7995	24.0576	22.8597
	Q9HZ98	ibpA	10	71.8	16.582	98.297	6.86E+09	73	149	27.5552	27.6062	27.2382	27.5711	27.766	28.1971	27.8201	27.6956
	Q9HZA2	PA3122	8	46.8	32.835	48.12	6.99E+08	43	297	25.3449	25.3787	25.7381	25.4349	25.2621	25.4165	25.4019	25.2064
e dehydrat	Q9HZA4	leuD	15	88.7	24.011	151	3.76E+09	101	212	27.1935	26.5888	25.9482	27.2882	25.3867	26.2156	25.0989	26.299
	Q9HZA6	fimV	16	31.6	96.928	163.64	2.11E+09	83	919	25.4192	25.7063	25.1661	25.7585	25.6997	25.6793	25.4022	25.5902
lase carbox	Q9HZA7	accD	13	50	31.843	115.89	1.23E+10	85	290	28.815	29.0512	29.2172	28.7944	28.6734	28.8078	28.9002	28.8509
	Q9HZA8	folC	7	29.8	46.505	27.439	7.4E+08	22	429	24.9793	25.3481	23.1687	25.2014	25.28	25.0725	25.4097	25.9899
NAD kinase	Q9HZC0	nadK	9	52.9	32.14	44.242	1.41E+09	36	295	25.4972	25.7017	25.7809	25.4433	25.1585	25.6468	25.6779	25.6576
	Q9HZC1	PA3087	8	38.3	37.211	61.177	3.84E+08	19	326	23.2439	24.9919	24.4753	24.6091	23.5607	23.3936	24.3213	21.8833
	Q9HZC3	PA3085	3	62.1	9.9651	18.671	2.04E+09	32	87	27.5483	25.8394	22.5194	27.4721	25.247	27.1146	25.5674	26.5043
	Q9HZC4	PA3084	12	61.3	29.99	47.181	1.19E+09	58	266	24.6939	25.213	25.2794	24.9876	24.9353	25.037	25.1309	25.2485
	Q9HZC5	pepN	39	58.6	100.05	273.56	6.61E+09	204	885	26.2329	26.5533	26.8954	26.6895	26.8348	26.6283	26.5247	26.3796
	Q9HZC7	PA3081	23	64.2	50.679	243.65	1.16E+10	167	455	27.4447	27.7279	27.7229	27.6151	27.4263	27.3104	26.9745	27.1414
	Q9HZD8	PA3070	7	21.5	36.148	24.481	8.33E+08	38	326	24.835	25.0917	25.165	24.677	24.5121	24.7524	25.1038	25.0241
	Q9HZF2	PA3056	5	60.1	18.132	37.922	1.14E+09	32	158	25.5653	25.4181	25.9175	25.7785	26.0555	25.4279	25.8818	25.5034
	Q9HZF5	PA3053	9	43.9	37.484	78.135	1.57E+09	44	335	26.1379	26.1583	26.3948	25.9677	25.6055	25.7964	25.6844	25.4049
dehydroge	Q9HZF8	pyrD	13	50	36.128	76.969	2.56E+09	71	342	26.4471	26.4817	26.7811	26.6544	26.7383	26.6573	26.4713	26.6484
methyltran	Q9HZG0	rlmL	10	21.8	81.154	78.327	5.36E+08	36	725	24.5005	23.9458	23.6464	24.8752	25.5841	24.6531	24.3608	25.1182
	Q9HZG1	PA3047	12	49.2	51.86	83.749	1.58E+09	69	476	25.5703	25.4142	25.503	25.7106	25.7128	25.9736	25.7818	25.9337
	Q9HZG2	PA3046	5	65.8	13.025	48.673	5.12E+09	34	114	28.4806	27.6872	27.562	28.6949	28.0891	28.2077	27.7985	27.8815

	Q9HZG8	PA3040	5	39.4	11.916	49.167	9.67E+08	35	109	25.1909	25.7694	25.6401	25.7986	25.9317	25.5476	26.3254	25.8337
	Q9HZH4	PA3034	3	20.5	20.988	7.259	1.37E+08	13	185	22.9409	23.797	23.7966	22.986	22.0784	22.7906	23.8848	22.4625
	Q9HZH8	moeA2	7	28.6	43.39	49.966	7.98E+08	27	405	24.832	25.0487	25.1614	24.6661	24.888	25.0233	25.1787	25.4557
	Q9HZI4	PA3022	11	50.7	29.579	47.059	1.19E+09	43	268	26.0146	26.2912	26.49	26.3842	26.0236	25.8074	25.9745	25.4371
	Q9HZI5	PA3021	9	56.2	14.206	87.737	1.61E+09	54	128	26.0806	25.5174	24.687	26.3427	25.4952	25.7001	25.4694	25.7683
	Q9HZI7	PA3019	17	37.8	71.155	91.792	1.65E+09	107	640	24.937	25.1255	24.8912	25.0434	25.0471	24.9994	25.143	25.4768
sal stress p	Q9HZI9	PA3017	5	69	15.844	72.881	1.71E+09	39	145	26.8803	26.1102	26.2586	26.8602	25.2642	26.0847	25.8746	26.4881
noyl-CoA is	Q9HZJ2	fadB	30	51	76.953	246.46	1.54E+10	276	715	27.413	27.506	27.4217	27.1675	27.1176	27.3579	27.3434	27.3093
acyl-CoA th	Q9HZJ3	fadA	13	61.6	41.643	171.65	2.45E+10	132	391	29.9661	30.0839	30.21	29.8637	29.717	29.6787	29.8929	29.6597
topoisomer	Q9HZJ5	topA	37	52.5	97.282	264.86	6.94E+09	168	868	26.0926	26.4775	25.9282	26.434	26.5613	26.676	26.4402	26.7204
	Q9HZJ6	PA3010	5	44.8	19.071	22.912	6.99E+08	30	172	25.0304	24.0951	25.3373	25.1319	24.9629	25.0636	25.1106	24.9564
	Q9HZJ9	psrA	12	70.8	25.784	80.299	3.87E+09	70	233	27.5507	27.3762	27.4312	27.3716	27.3571	27.2281	27.4233	27.0736
hexosamin	Q9HZK0	nagZ	13	50.9	36.101	98.981	6.58E+09	107	332	27.6612	27.7327	27.6435	27.5871	27.7799	27.6286	27.7776	27.6441
	Q9HZK2	PA3003	9	72.2	22.474	63.103	8.09E+09	91	209	28.9062	28.7137	27.7991	28.8678	28.8847	28.9386	28.678	28.8287
DH-quinor	Q9HZK6	nqrA	18	58	48.081	205.76	6.22E+09	115	445	27.2029	27.3561	27.2257	27.1544	27.3984	27.4937	27.3183	27.446
DH-quinor	Q9HZK8	nqrC	6	37.2	27.779	18.73	5.8E+08	22	261	23.7995	24.9441	23.1289	24.9583	24.8611	25.1505	24.7696	25.3104
	Q9HZL4	PA2990	9	38.3	26.887	68.986	1.06E+09	47	240	25.2097	25.4468	25.2666	25.277	24.7263	24.6867	25.1226	24.8585
	Q9HZL5	PA2989	3	10.2	28.376	11.672	1.91E+08	14	254	24.215	24.0653	24.2831	24.1165	23.8883	23.9635	23.7759	23.0788
system ATI	Q9HZL7	lolD	4	48	24.65	92.148	1.95E+09	33	227	26.9076	27.0694	27.1151	26.997	27.0224	26.9298	27.3388	27.3081
34 protein I	Q9HZM4	PA2980	5	77	6.7208	40.865	5.69E+09	35	61	29.1363	27.5088	26.7242	28.8542	27.2333	28.7784	27.4868	27.9398
ulosonate	Q9HZM5	kdsB	10	44.5	27.646	94.232	3.99E+09	59	254	26.976	26.9898	27.1856	26.6495	26.9121	26.9045	27.0789	26.9339
	Q9HZM6	ptpA	6	52.6	17.152	48.289	3.92E+08	28	154	24.5824	21.9792	24.5699	24.7665	24.3109	23.8563	24.4049	24.3418
bonuclease	Q9HZM8	rne	38	39.4	117.46	323.31	5.13E+10	346	1057	29.3661	29.6506	29.2775	29.4417	29.7782	29.7446	29.8216	29.873
unit pseud	Q9HZM9	rluC	8	26.7	35.641	28.968	4.75E+08	30	318	24.3816	24.5141	24.3769	24.2585	24.2898	24.6277	24.4245	24.5343
	Q9HZN0	PA2974	9	54.8	25.322	46.418	9.67E+08	35	230	24.5718	25.1887	25.0778	24.4678	24.7912	25.1036	25.2745	24.895
	Q9HZN1	PA2973	5	24.2	36.063	56.577	5.93E+08	23	326	24.9419	25.3695	23.2682	25.1472	25.6718	25.405	25.43	25.7693
e protein F	Q9HZN2	PA2972	8	58.3	21.054	53.154	1.93E+09	49	192	26.4332	26.7095	25.7432	26.571	26.8028	26.8082	26.7547	26.7927
	Q9HZN3	PA2971	6	50.6	19.569	33.006	1.72E+09	32	178	27.1245	26.9623	21.8184	27.059	26.9205	27.3021	26.8496	27.2135
osomal pro	Q9HZN4	rpmF	4	55	6.7914	46.994	8.62E+10	69	60	32.2442	32.2333	31.5283	32.2188	32.3711	32.1846	32.3851	32.1124
ate acyltrar	Q9HZN5	plsX	4	19	35.549	25.084	2.27E+08	19	336	24.0587	23.6917	24.0774	22.9629	22.539	23.9194	24.128	23.9043
	Q9HZN6	pabC	3	15.1	29.886	7.8268	3.79E+08	20	271	23.9971	24.2086	24.4687	24.1377	24.046	24.1781	24.0595	23.8073
nidylate ki	Q9HZN8	tmk	7	50.5	23.109	59.816	1.35E+09	38	210	25.5314	25.5034	25.039	25.4723	25.5577	25.6921	25.4765	25.5263
	Q9HZN9	PA2959	12	77.9	28.114	150.8	5.62E+09	84	258	27.2027	26.9029	26.6734	27.2763	27.0769	27.2687	26.8134	27.1615
	Q9HZP0	PA2958	7	30	42.109	29.442	4.58E+08	20	377	24.4405	25.0819	22.9645	24.77	25.3898	25.1368	25.9351	25.3946
	Q9HZP4	PA2954	7	57.7	21.24	27.207	4.77E+08	25	189	25.157	25.1542	24.918	25.3424	23.5891	25.0531	24.923	24.6756
rotein-ubic	Q9HZP5	PA2953	32	70.8	59.928	284.94	4.37E+10	324	551	29.0065	29.1324	28.9512	28.8337	28.9864	29.14	29.0163	29.0914
r flavoprot	Q9HZP6	etfB	13	74.7	26.376	204.77	1.23E+11	236	249	31.3362	31.3783	31.6877	31.4112	31.2621	31.3364	31.3765	31.1592
flavoprote	Q9HZP7	etfA	17	76.7	31.422	309.48	1.59E+11	411	309	32.2302	32.2127	32.5427	32.3695	32.2502	32.1927	32.3386	32.0494
reductase	Q9HZP8	PA2950	26	60.6	43.528	323.31	4.65E+10	245	398	29.6535	29.973	29.9262	29.5046	29.3534	29.5155	29.4915	29.6114
rotein Cob\	Q9HZQ2	cobW	15	51.5	41.206	323.31	9.25E+09	115	375	27.4834	27.4466	27.4045	27.2593	27.322	27.5041	27.8028	27.5295
	Q9HZQ5	PA2942	4	20.1	36.545	36.118	4.03E+08	23	338	24.3382	24.347	24.8615	24.122	24.4455	24.6165	24.2635	24.6423
	Q9HZR5	morB	9	47.7	39.631	85.21	1.03E+09	62	369	24.9862	24.642	24.9239	25.1251	24.9612	24.8683	24.8822	24.7845
	Q9HZS9	PA2918	3	22.2	26.738	18.165	4.82E+08	27	257	24.9592	24.9802	25.1548	24.7625	25.2915	25.2158	25.1843	24.9703
	Q9HZT2	PA2915	8	49.3	32.013	74.381	1.01E+09	43	288	25.1832	25.2888	25.7112	25.1629	25.5469	25.6225	25.9597	25.7235
n-8X methy	Q9HZU2	cobH	5	24	21.65	30.371	1.07E+09	31	208	25.4974	25.6483	25.6282	25.5576	25.6955	25.6186	25.9496	25.6834
C(20)-meth	Q9HZU3	cobI	7	42.4	27.274	38.398	9.25E+08	35	250	25.7839	25.8072	25.8225	25.6621	25.51	25.887	25.7661	25.6209
	Q9HZU4	cobJ	3	8.4	58.982	11.907	79832000	12	559	23.8387	22.9542	22.3334	22.9796	22.2879	23.1738	23.0468	23.3131
	Q9HZU5	PA2902	8	35.5	30.448	62.753	7.95E+08	16	282	25.9929	26.2204	23.0691	26.6439	21.829	25.5516	23.4946	25.6729
	Q9HZW2	atuR	4	29.3	22.057	47.444	1.14E+09	33	198	26.0255	26.2189	26.6305	25.8384	25.9013	25.5593	23.3364	25.4384
	Q9HZX5	PA2871	8	44.2	28.695	53.46	1.57E+09	50	265	25.3073	25.5934	25.4565	25.5394	25.8893	25.4251	25.6529	25.4223
	Q9HZY0	mttC	5	30.3	29.544	75.008	6.54E+08	23	267	25.1471	25.1531	23.1771	25.4389	25.4791	25.4023	25.1198	25.0593
elongatio	Q9HZY5	greB	6	42.9	19.144	37.176	6.16E+08	22	168	25.1148	23.1957	24.8592	25.2134	25.5744	25.5047	25.6444	25.1958
	Q9HZY7	PA2857	7	46.3	24.585	63.884	1.47E+09	48	227	25.5533	25.8423	26.2661	25.5105	26.2609	26.3997	26.3036	25.9971
sterase Tes	Q9HZY8	tesA	5	51.2	21.037	55.471	1.28E+09	45	201	26.3699	25.3785	25.1471	26.2427	25.3275	25.9718	25.8227	25.94
	Q9HZZ0	PA2854	16	59.8	34.799	116.22	1.49E+10	147	323	28.1609	28.419	28.3034	28.1182	27.9432	27.9783	28.0472	28.0169
gation fact	Q9HZZ2	efp	9	38.3	20.985	80.928	9.93E+10	119	188	31.5017	31.7483	31.4751	31.6815	31.7598	31.6797	31.558	31.5754
	Q9HZZ3	ohr	5	54.2	14.522	98.69	6.43E+09	40	142	28.1998	27.9718	27.4882	28.1851	28.2197	28.5638	28.542	28.6352
	Q9I000	PA2843	11	35.9	49.851	30.947	4.71E+08	23	448	24.9428	24.8766	23.0302	25.4438	23.6146	25.1236	24.843	25.3104
ent RNA he	Q9I003	deaD	26	54.9	62.107	190.04	1.3E+10	186	567	27.7622	28.0878	27.8946	27.7085	27.7739	27.9001	27.9041	28.0161
S-methylt	Q9I011	tpm	6	49.5	24.875	14.035	5.22E+08	22	218	25.2902	23.0612	25.3819	25.1424	25.1322	25.2859	25.4558	25.166
	Q9I012	PA2831	12	49.3	42.933	89.604	1.67E+09	59	375	26.1102	26.2791	26.2832	26.2164	26.207	26.0252	25.968	25.9239
	Q9I014	PA2829	6	56.7	16.431	27.592	3.84E+08	23	150	24.4372	24.1316	24.4244	24.3927	24.7485	24.5867	24.8094	24.5049
	Q9I015	PA2828	13	53.8	44.799	113.22	6.89E+09	100	403	27.3151	27.0408	26.9283	27.4771	27.7879	27.6191	27.4114	27.3774
hione pero	Q9I017	PA2826	6	53.4	17.659	35.335	1.6E+09	38	161	26.5456	26.9409	26.9797	26.5071	26.5483	26.7566	26.8339	26.575
	Q9I020	PA2823	12	50	33.792	118.1	5.49E+09	82	296	27.5433	27.7291	27.9856	27.4322	27.6479	27.8015	27.8888	27.8149
	Q9I021	PA2822	2	25.6	17.129	19.697	1.18E+09	20	160	26.3023	26.1342	25.8884	26.373	26.1618	26.3687	26.1997	26.4015
	Q9I026	PA2817	9	69.9	16.136	62.485	3.59E+09	52	136	28.2183	26.5931	25.2203	28.0361	26.7035	27.8875	26.0415	27.3068
	Q9I030	PA2813	8	45.6	23.404	41.707	3.03E+09	41	206	27.6954	28.4766	28.5781	28.1874	28.3533	28	28.1974	27.7161
	Q9I031	PA2812	13	64.8	34.54	63.236	1.72E+09	75	310	25.8789	25.9054	25.9405	25.5522	25.6215	25.729	25.8386	25.6711
	Q9I034	copR	5	32.7	25.716	11.666	2.78E+08	17	226	23.1724	22.3924	24.2198	24.1662	24.3992	22.833	24.1684	24.5324
cyano-7-de	Q9I037	queF	10	48.2	30.848	103.27	2.1E+09	57	276	25.7173	25.5961	25.15	25.8754	25.9256	26.0215	25.9938	25.7258
	Q9I038	PA2805	9	90.8	9.7039	124.74	5.15E+09	49	87	29.4432	27.3565	25.842	29.4393	25.9791	28.3777	26.2296	27.5715
	Q9I045	PA2798	16	56.3	43.485	124.72	2.23E+09	75	394	26.3005	25.8457	26.1775	26.3219	26.737	26.6188	26.8905	26.5616
	Q9I046	PA2797	6	49.4	17.71	39.447	2.1E+09	45	160	27.1942	27.0656	27.0202	27.1981	27.2756	27.1865	27.2742	26.9787
ransaldolas	Q9I047	tal	17	63.2	33.946	156.03	8.88E+09	143	307	27.3117	27.5324	27.664	27.1059	27.6914	27.5636	27.9383	27.7046
drouridine	Q9I048	dusA	12	60.2	36.929	114.35	2.03E+09	62	332	25.9355	26.2441	26.3262	26.3418	26.8138	26.5776	26.2238	26.1242
	Q9I067	PA2776	14	43.3	46.693	126.95	6.69E+09	98	427	27.8551	27.7553	28.0167	27.7416	27.3469	27.2577	27.2338	27.1851
ized isome	Q9I073	PA2770	7	54.1	28.258	26.242	9.69E+08	24	259	26.1714	26.3723	24.0316	26.1401	26.18	26.4375	26.0816	26.303
	Q9I078	PA2765	14	64.2	32.396	128.97	1.09E+10	119	299	28.1073	28.0331	27.7969	27.9923	28.2054	28.3094	27.8287	28.1583
	Q9I083	PA2760	6	21.2	46.85	20.05	3.18E+08	20	425	24.0621	24.4459	24.1236	24.5024	24.2991	23.3374	24.394	24.2117
	Q9I098	PA2745	7	37.6	31.258	33.931	3.26E+08	33	287	24.0414	24.0669	24.0819	23.9992	23.7767	23.7909	23.7528	23.6994
nine--tRNA	Q9I099	thrS	29	51.2	73.079	280.09	2.13E+10	188	640	30.068	30.0971	29.4921	30.0454	30.6177	30.4811	30.343	30.5658
initiation	Q9I0A0	infC	13	60.1	20.882	152.51	3.69E+10	146	183	29.4605	29.7928	29.7328	29.8441	29.8409	29.7687	29.8839	29.7396
osomal pro	Q9I0A2	rplT	3	16.9	13.365	10.922	2.01E+10	20	118	29.6972	30.3501	29.4493	30.4168	30.7463	30.4668	30.0645	30.3455
tRNA ligas	Q9I0A3	pheS	18	75.4	38.063	195.55	1.42E+10	168	338	28.3554	28.418	28.2985	28.0693	28.4346	28.5572	28.5788	28.7857
-tRNA ligas	Q9I0A4	pheT	25	45.1	86.799	236.83	1.27E+10	153	792	27.516	27.8399	27.4407	27.5384	28.0598	28.0006	28.0398	28.4295
	Q9I0C2	PA2720	6	40.6	23.816	25.027	5.06E+08	29	212	24.6889	25.0092	24.9141	24.6389	25.0759	25.0346	24.8853	24.5556
teine synth	Q9I0D3	cysK	16	74.1	34.309	249.62	1.5E+10	139	324	28.468	28.3797	28.7046	28.334	28.3503	28.3534	28.5746	28.3386
	Q9I0D5	PA2707	18	58.7	32.205	105.6	7.69E+09	122	281	28.2557	28.5745	28.6569	27.9696	28.19	28.0838	28.118	28.0456
	Q9I0D7	PA2705	4	12	45.584	7.1359	78812000	14	393	22.7192	22.7913	23.3986	23.6442	23.0233	24.1044	22.6566	22.9599
	Q9I0F0	PA2692	4	30.5	19.143	17.448	4.72E+08	22	174	24.3353	24.7044	24.4154	24.7153	24.4984	24.9338	24.6947	24.4536
	Q9I0F5	PA2683	8	46.6	33.957	52.741	7.53E+08	45	320	24.6602	25.0393	25.0807	24.6844	25.0803	24.9123	25.5454	25.1111
	Q9I0H1	PA2667	14	78.6	13.379	120.69	2.75E+10	103	117	29.4301	29.4305	28.8493	29.296	29.5552	29.734	29.6575	29.6367
	Q9I0H9	PA2659	4	60.8	11.179	53.102	6.91E+09	49	102	29.3665	28.5008	28.1828	29.2583	28.3452	29.0982	27.5659	28.9834
	Q9I0I1	PA2657	5	36.8	24.729	25.126	8.23E+08	36	223	25.1744	24.6261	25.1455	24.9657	25.0009	25.3469	25.6088	25.3629
	Q9I0I4	1251;PA26	7	16	77.124	21.574	2.33E+08	22	714	23.8028	24.1887	23.4321	24.002	24.2049	23.7513	22.1503	23.8908
oxidoredu	Q9I0J6	nuoG	43	65.1	98.999	323.31	2.55E+10	382	905	27.5429	27.7949	27.4512	27.5965	28.032	28.1201	27.9488	28.094
oxidoredu	Q9I0J7	nuoF	23	66.5	48.678	225.44	1.52E+10	182	448	28.2766	28.3454	28.3179	28.1963	28.226	28.4734	28.436	28.5204
oxidoredu	Q9I0J8	nuoE	7	61.4	18.088	75.262	8.55E+09	64	166	28.7236	28.3003	27.5489	28.6212	27.9178	28.6908	28.3642	28.9526
oxidoreduc	Q9I0J9	nuoC	20	48.4	68.322	161.01	4.7E+09	119	593	26.2098	26.4187	26.0076	26.2028	26.7551	26.6603	26.5671	26.872
oxidoredu	Q9I0K0	nuoB	8	46.7	25.425	112.75	5.6E+09	63	225	27.7101	28.0591	27.9123	27.6283	27.4488	27.8658	27.6097	27.8958
ocitrate lya	Q9I0K4	PA2634	23	57.1	58.886	205.81	1.09E+10	161	531	27.3584	27.7744	27.7366	27.1985	27.5574	27.7096	27.6873	27.572
	Q9I0K5	PA2633	7	50.5	29.483	81.251	6.37E+08	33	273	25.2853	25.3568	25.1245	25.4841	24.6367	24.8958	24.8274	24.8161
	Q9I0K7	PA2631	6	65.2	16.04	51.003	2.81E+09	39	141	27.7974	27.7305	27.3022	27.7289	27.4462	27.4273	27.6413	27.4502
	Q9I0K8	PA2630	10	39.6	44.057	118.87	2.69E+09	71	389	26.5316	26.616	26.3243	26.4818	26.3847	26.6105	26.5588	26.8506
enization p	Q9I0L1	hflD	6	38.8	23.062	52.471	3.27E+08	20	206	22.6407	22.5625	23.6764	24.8364	24.5132	24.392	24.4241	24.5884
2-thiourid	Q9I0L2	mnmA	16	52	41.886	125.97	1.22E+10	140	375	27.9958	28.1056	28.1455	28.1113	28.3141	28.1626	28.2249	28.059
	Q9I0L4	idh	41	66.9	81.633	323.31	4.72E+10	389	741	28.4847	28.6894	28.4248	28.3727	28.9218	28.9406	28.9395	29.4017
ehydrogen	Q9I0L5	icd	34	71.8	45.577	323.31	9.81E+10	427	418	29.8798	29.9799	30.084	29.6789	30.0945	30.2359	30.2197	30.0354
	Q9I0L8	clpA	38	60.4	84.01	273.03	1.75E+10	261	758	28.0427	28.1252	28.3397	27.5717	27.3338	27.4192	27.6572	27.6507
edoxin redu	Q9I0M2	trxB1	14	79.4	33.818	323.31	7.31E+10	207	316	31.2554	31.2751	31.2774	31.2501	31.0665	31.0108	31.2494	31.0162
lipoprote	Q9I0M4	lolA	13	68.8	23.072	120.81	1.37E+10	107	208	28.0576	28.2942	27.9902	28.1379	28.126	28.2812	28.2922	28.1399
	Q9I0M5	PA2613	14	46.3	49.079	71.144	8.5E+08	41	441	24.9383	24.6363	25.4148	25.0419	24.9528	25.7404	25.4146	25.5378
ne--tRNA li	Q9I0M6	serS	26	73.9	47.231	323.31	2.25E+10	223	426	28.4716	28.3803	28.5415	28.3253	28.5913	28.4543	28.6519	28.4986
rochlorin fe	Q9I0M7	cysG	9	31.4	50.37	42.367	8.26E+08	28	465	25.3004	25.4502	25.1267	25.1566	25.7515	25.7151	26.0628	25.8431
	Q9I0M9	PA2609	8	41.6	36.625	62.363	8.48E+08	42	327	25.2552	25.17	25.0911	25.4977	25.4298	25.4829	25.6338	25.6553
sferase Tus	Q9I0N3	tusD	3	43.5	14.086	37.528	1.28E+09	25	131	26.7626	26.7246	23.899	26.6157	26.8534	27.0782	26.6337	26.3499
	Q9I0Q4	PA2582	12	72.3	19.446	43.958	5.76E+09	60	177	28.0253	28.2979	27.6193	28.2806	28.4231	28.0925	27.976	27.9705
an 2,3-dio:	Q9I0Q7	kynA	5	33	32.931	28.997	5.94E+08	21	288	24.9571	25.1312	22.8638	24.9612	25.2535	25.7844	25.6422	25.4207
	Q9I0S7	PA2557	9	28.5	62.629	28.985	7.95E+08	29	564	26.265	25.9573	26.623	26.0763	25.8112	26.1266	26.5518	23.9149
	Q9I0T3	PA2551	8	41.9	35.144	34.219	2.84E+08	39	310	23.9091	23.8476	23.9946	22.5403	23.8661	23.7648	23.6632	24.0496
	Q9I0T9	xthA	13	59.6	31.003	58.66	3.2E+09	74	270	27.1623	27.2206	27.3531	26.8758	27.0455	27.1406	27.2164	27.1957
	Q9I0U7	PA2537	4	34	23.149	30.26	1.44E+08	14	209	23.8767	23.1014	23.7113	23.7332	23.9507	23.8581	24.0787	23.2069
	Q9I0U9	PA2535	10	49.2	36.262	37.254	5.05E+08	39	331	24.4752	24.713	25.0853	24.3286	24.1049	24.6055	24.329	24.3002

	Q9I0V3	PA2530	9	37.2	48.959	79.317	9.55E+08	45	441	25.7907	25.5357	25.8453	25.2715	26.1856	25.8123	25.8687	25.9544
	Q9I0Z0	mexT	3	12.7	38.222	18.058	2.2E+08	14	347	22.2821	24.4896	24.6342	24.4621	23.0266	24.1063	24.5928	24.3527
	Q9I0Z9	PA2483	9	57.1	36.102	47.687	7.35E+08	48	333	25.0704	24.1521	24.7448	24.5808	24.5456	24.6923	24.6545	25.0705
	Q9I118	PA2464	5	47.1	17.626	14.585	5.65E+08	16	174	24.8516	24.7566	24.1738	25.2107	25.4906	25.1319	24.9509	24.9977
	Q9I127	PA2455	3	22.3	20.332	9.3627	1.85E+08	10	175	23.2455	23.9746	23.542	24.0913	24.3125	23.9538	23.6013	22.8691
vage systen	Q9I136	gcvH1	3	22.8	13.793	11.284	5.21E+09	29	127	28.7609	28.3069	27.1654	28.6564	27.297	28.4705	27.0848	28.1027
genase (de	Q9I137	gcvP1	30	46	103.92	286.25	5.56E+09	167	959	26.197	26.7832	25.5809	26.6041	26.4213	26.4268	25.9185	26.7484
ase 1;Serin	Q9I138	glyA1;glyA2	11	37.6	44.973	143.52	2.83E+09	82	418	26.5257	26.487	26.5922	26.1168	25.6688	26.2123	25.6894	25.851
methyltran	Q9I140	gcvT2	18	65.7	39.882	157.51	2.65E+10	166	373	29.0345	29.1477	29.29	29.0827	28.833	28.9134	28.7915	28.6642
	Q9I192	fpvR	3	15.1	37.022	18.899	3.46E+08	16	331	24.1716	24.3267	24.2908	24.628	24.6189	24.782	24.8057	24.6359
	Q9I1A0	PA2379	6	58.3	16.618	54.083	7.62E+08	34	156	25.1128	24.9608	23.4526	24.8318	25.6325	25.4382	25.4165	25.3217
	Q9I1A1	PA2378	20	42.8	83.665	152	3.49E+09	88	771	26.2319	26.2063	24.5283	26.1066	26.8879	26.5853	26.577	26.5943
	Q9I1F5	PA2323	16	49.5	59.96	107.24	1.98E+09	78	541	26.1361	26.4881	26.1	26.3193	25.6751	25.6439	25.7719	26.1588
	Q9I1F6	gntR	9	32.7	37.147	26.859	4.12E+08	24	343	23.0806	24.7342	25.0573	24.7984	24.3437	24.6901	24.4541	24.6811
	Q9I1F8	PA2317	6	24.5	47.855	30.961	5.71E+08	28	432	25.3604	25.3253	25.1671	24.7271	24.4893	24.4324	22.5685	24.7766
	Q9I1H4	PA2301	6	42.2	19.33	23.787	3.19E+08	25	180	24.2821	24.2122	24.298	24.1017	24.1165	24.3117	24.4012	24.0632
	Q9I1I5	gcd	8	16.6	86.217	45.453	5.19E+08	33	803	24.2431	25.0158	23.6977	24.9942	24.5647	23.0348	23.7121	24.5181
	Q9I1K9	PA2264	10	64.3	26.358	53.068	2.84E+09	40	238	28.0097	27.4373	26.2981	28.1827	26.3749	26.6832	26.1317	26.7574
	Q9I1L6	ansA	7	30.5	34.755	36.784	8.34E+08	32	328	25.212	25.2006	25.1587	25.0187	24.8291	25.3747	25.272	25.5023
poyl dehyd	Q9I1L9	lpdV	29	81	48.588	323.31	6.34E+09	97	464	25.2096	27.0905	28.8968	24.61	24.623	23.1696	25.586	23.7051
	Q9I1P0	PA2229	6	33.5	26.56	53.083	1.37E+09	30	239	26.2076	26.1108	26.0889	26.552	26.3701	26.4874	26.0746	26.4317
	Q9I1R3	PA2204	22	74.6	29.308	173.37	1.03E+10	141	268	27.6846	27.954	27.3361	27.7952	27.5688	27.5181	27.5523	27.5261
	Q9I1R8	PA2199	5	26.8	30.886	53.44	8.86E+08	34	291	25.1191	25.1289	26.5795	25.438	25.7147	25.0948	25.1261	25.2246
	Q9I1S0	PA2197	14	54.2	37.135	81.716	1.99E+09	84	345	25.9912	25.9237	26.1648	25.8022	25.6666	25.6213	25.793	25.4734
17 protein I	Q9I1Z9	PA2116	8	38.1	29.221	57.867	4.12E+09	76	265	27.4819	27.7818	27.9646	27.0689	27.141	27.374	27.6002	27.3418
71 protein I	Q9I203	PA2112	14	82.2	26.334	191.66	1.47E+10	131	247	28.5538	28.5455	28.5458	28.3522	28.1278	28.6722	28.6792	28.5412
	Q9I204	PA2111	10	47.7	26.218	81.769	2.57E+09	62	237	25.6971	26.0111	26.0052	25.8046	25.9836	25.8951	25.8665	25.5984
	Q9I205	PA2110	3	11.8	33.06	13.48	5.96E+08	19	313	24.7717	25.3443	25.4161	24.9424	24.9645	25.2386	25.5399	25.5759
gation facto	Q9I244	fusB	32	65.7	77.573	196.28	6.4E+09	144	702	26.7166	26.8868	27.0842	26.6804	27.4638	27.1896	27.6299	27.1168
	Q9I271	PA2044	33	67.3	70.083	323.31	1.32E+10	218	624	27.776	27.814	28.0698	27.9166	27.6287	27.4101	27.6079	27.4628
	Q9I275	PA2040	9	42.1	50.931	22.956	5.29E+08	15	458	24.9121	24.7487	24.3675	24.9514	25.5606	26.0557	23.4181	26.0321
nosphate u	Q9I291	galU	17	69.9	31.236	195.25	2.22E+10	144	279	29.1758	29.1903	29.1551	29.1685	29.3948	29.574	29.8511	29.7061
	Q9I297	liuB	8	25.6	57.433	28.574	5.44E+08	28	535	24.5101	24.6567	24.5758	24.5896	24.5297	24.2897	24.5102	24.3535
	Q9I299	liuD	13	30.8	71.282	53.263	9.83E+08	35	655	25.6392	25.9642	26.2999	22.5565	25.348	25.1618	25.652	25.4297
	Q9I2A2	fahA	4	16	46.341	25.574	4.88E+08	22	432	24.8962	24.9694	24.9144	24.6105	24.8001	24.8948	24.9835	24.7482
oA acetyltra	Q9I2A8	atoB	20	75.1	40.405	269.78	8.96E+09	168	393	27.2095	27.0705	27.4822	26.8054	26.9144	26.5708	26.8861	26.5057
	Q9I2E0	PA1964	14	48.4	57.917	86.026	1.72E+09	45	521	26.9802	27.2361	26.5482	26.2462	26.2577	27.6952	26.895	27.4311
	Q9I2F4	rbsK	6	27.9	31.846	41.434	3.07E+08	26	308	24.1166	24.3978	24.364	24.2405	23.8949	23.9553	24.1514	23.9222
	Q9I2P3	PA1852	3	52.7	9.97	46.929	5.35E+08	21	91	24.573	23.7877	22.9871	24.9169	24.7117	25.6216	24.9343	25.7335
enesis prot	Q9I2P8	nfuA	6	44.3	21.136	103.42	5.16E+09	51	194	28.5655	28.1706	27.2825	28.8686	27.9725	28.2943	27.8359	28.4545
iionine syn	Q9I2Q2	metH	22	31.4	135.06	89.763	1E+09	78	1234	25.3419	25.073	22.546	25.0926	25.7741	25.6806	25.1891	25.24
	Q9I2Q7	cysI	26	59.2	62.132	189.69	6.14E+09	145	552	26.6417	26.7021	26.2736	26.9575	27.1213	26.9989	26.7419	26.7064
	Q9I2Q8	PA1837	7	57	18.765	150.17	2.5E+09	53	165	26.8707	27.0434	27.163	27.0954	27.4506	27.2749	27.3246	26.9113
	Q9I2R2	PA1833	14	58.5	34.551	120.75	8E+09	100	330	27.6394	27.6466	27.9331	27.4927	27.2893	27.1514	27.2749	27.0376
	Q9I2R5	PA1830	5	63.5	11.11	28.27	1.02E+10	46	104	29.9074	28.2792	27.5519	29.9109	27.6152	29.727	28.7862	29.5747
	Q9I2R7	PA1828	9	49.4	26.919	74.277	2.85E+09	51	255	26.6661	26.8449	26.6407	26.4819	26.3306	26.207	26.4297	26.4816
	Q9I2S3	fimL	16	47.9	60.868	84.927	2.31E+09	87	562	26.1565	25.9268	26.0071	25.8324	25.9651	26.1355	26.0068	26.4558
	Q9I2S4	PA1821	8	41.9	29.989	72.215	1.44E+09	53	270	25.4713	25.6619	25.705	25.0573	25.8998	25.5848	25.6147	25.5965
	Q9I2S7	ldcA	16	33.4	82.756	72.976	9.66E+08	75	751	24.7491	24.8042	24.542	25.0338	24.8819	25.0048	25.0292	25.165
ponuclease	Q9I2S9	rnhA	8	58.1	16.697	67.95	1.18E+09	37	148	25.6251	25.5553	25.5847	25.4986	25.5262	25.6308	25.6526	25.6136
lglutathion	Q9I2T1	gloB	8	45	28.865	41.275	1.12E+09	46	258	25.9131	24.8486	25.3861	25.9331	25.4495	25.5395	25.8472	25.3864
	Q9I2T4	PA1810	27	56.1	70.473	167.73	4.88E+09	148	615	26.2847	26.4328	26.5416	26.5084	26.9543	26.615	26.6026	26.3973
ylprolyl isor	Q9I2T8	ppiD	29	55.7	68.741	290.96	6.96E+09	158	621	27.2728	27.4441	26.9023	27.5318	27.526	27.4103	27.3135	27.631
on proteas	Q9I2T9	lon	51	60.8	88.646	323.31	1.61E+10	332	798	27.3853	27.5126	27.309	27.417	27.4601	27.5443	27.4566	27.5774
otease ATF	Q9I2U0	clpX	24	66	46.991	217.62	3.64E+10	256	426	29.5141	29.6672	29.6633	29.2883	29.521	29.655	29.7338	29.609
protease p	Q9I2U1	clpP1	2	18.8	23.502	46.733	1.64E+10	44	213	29.425	30.0884	28.8562	29.8796	30.0948	30.0549	29.8054	30.0718
rigger facto	Q9I2U2	tig	41	87.4	48.581	323.31	4.15E+11	814	436	32.5307	32.4961	32.5217	32.5613	32.7696	32.6298	32.8644	32.645
e cyclohyd	Q9I2U6	folD	14	69.7	30.549	134.17	1.48E+10	125	284	28.11	27.9514	27.993	28.0787	27.9928	28.1442	27.9922	28.0973
ine--tRNA I	Q9I2U7	cysS	33	68.7	51.276	295.4	2.48E+10	317	460	28.2083	28.3154	28.5139	28.2628	28.5823	28.4333	28.5269	28.2294
nine--tRNA	Q9I2U8	glnS	38	66.4	62.912	323.31	5.45E+10	297	556	29.8639	29.9539	30.0646	29.9531	30.1506	30.0029	30.0987	29.8976
olyl cis-tran	Q9I2U9	ppiB	8	47.3	18.126	108.9	5.54E+10	115	165	30.7328	30.5686	29.6199	30.7133	30.9422	30.7805	30.5527	30.6858
	Q9I2V1	PA1791	6	23.3	48.559	24.371	4.32E+08	20	443	23.3073	25.0247	23.1893	25.1678	25.3517	25.3193	23.5729	25.1089
	Q9I2V2	PA1790	3	17.9	23.344	26.255	4.15E+08	19	207	24.6836	24.9656	24.6009	24.7294	24.6246	24.8518	24.5327	21.9068
tate hydrat	Q9I2V5	acnB	51	65.7	93.627	323.31	8.96E+10	474	869	29.486	29.649	29.386	29.3561	29.577	29.8537	29.6011	30.0176
-methyl-2-(	Q9I2W7	PA1772	13	95.1	17.404	129.83	1.2E+10	120	162	28.8963	28.7485	28.3165	29.0024	28.4013	28.4074	28.3271	28.4151
nolpyruvat	Q9I2W9	ppsA	48	64.6	85.816	323.31	4.35E+10	437	791	28.7116	28.9388	28.8965	28.4502	28.9463	29.202	29.4302	29.4294
ruvate syn	Q9I2X0	PA1769	12	55.5	31.106	69.799	1.17E+09	45	274	26.0493	26.1202	26.4634	25.9364	25.5899	25.858	25.9035	25.8075
	Q9I2X3	PA1766	10	48.3	36.249	61.081	1.7E+09	48	327	25.8171	25.5463	26.1077	25.4141	25.9628	26.0904	26.2148	26.0061
rine phospl	Q9I2Y2	thrH	10	53.7	23.533	76.089	8.14E+09	63	205	28.6948	28.8572	27.2012	28.4043	29.2009	29.1024	28.6855	28.7981
o-3-deoxyh	Q9I2Y7	PA1750	12	47.8	39.132	147.36	9.15E+09	106	358	27.6146	27.4175	27.7593	27.412	27.922	27.9984	27.9579	27.9498
	Q9I2Y8	PA1749	4	25.5	17.671	21.932	2E+09	23	161	27.2954	27.0846	26.276	27.4644	24.3524	27.1707	26.7078	26.8748
	Q9I2Y9	PA1748	4	34.1	24.412	13.585	2.64E+08	19	229	23.8521	24.3275	23.5487	23.0333	24.5441	24.2608	24.1053	24.2185
	Q9I2Z5	PA1742	12	70.4	27.361	118.71	9.8E+09	97	240	28.5017	28.7073	28.2983	28.5726	28.1956	28.1641	28.2632	28.2975
	Q9I308	PA1729	7	55.8	23.959	41.682	3.82E+08	23	215	25.2358	23.1822	24.5194	24.9917	23.3069	24.6057	24.4813	25.011
	Q9I325	pcrH	7	63.5	18.35	53.634	2.05E+09	46	167	26.6684	26.4316	25.7647	27.1884	25.1326	25.9911	23.7758	25.9793
e-phosphat	Q9I340	mtnC	4	26.5	26.472	59.324	1.1E+09	30	249	25.798	25.8568	25.3775	25.8023	25.8589	25.8929	25.6996	25.5143
ctone diox	Q9I341	mtnD	10	78.5	20.552	128.68	6.6E+09	94	181	27.936	28.1011	27.9434	27.9075	27.7836	27.8461	27.7221	27.7406
se-1-phosp	Q9I342	mtnB	6	57.1	22.867	51.331	8.88E+08	28	205	25.6802	26.05	26.0977	25.7789	25.8288	25.8596	26.1423	25.9977
ismate syni	Q9I344	aroC	15	63.6	38.987	118.36	6.04E+09	85	363	27.1304	27.4744	27.2645	27.199	27.4809	27.5819	27.5022	27.5759
L3 glutam	Q9I347	prmB	7	28	34.229	64.758	1.23E+09	51	304	25.0999	25.191	25.0377	25.0125	25.3982	25.4727	25.1605	25.358
	Q9I348	PA1677	9	60.1	21.034	101.79	2.65E+09	73	198	26.1963	25.7313	25.9523	26.1483	26.3157	26.1691	25.9336	26.2126
ycIohydrolа	Q9I351	folE2	10	79	20.236	144.26	4.41E+09	57	181	27.1658	27.4356	27.6407	27.2179	27.4503	27.7524	27.8333	27.7979
	Q9I370	PA1655	5	36.5	22.738	33.118	1.11E+09	20	200	24.36	25.7833	26.1911	26.9714	26.7794	26.4978	26.6583	26.3654
	Q9I381	PA1644	4	33.2	21.838	43.125	7.3E+08	23	202	25.8359	25.9856	26.1343	24.7842	26.2806	25.9756	26.2559	26.0294
lenouridine	Q9I382	selU	10	39	41.845	64.686	1.12E+09	47	369	25.3396	25.0196	25.1017	25.303	25.5106	25.3559	25.529	25.0694
de, water d	Q9I383	selD	12	45.3	36.353	54.079	5.37E+09	71	344	28.0834	28.2405	27.7985	27.9106	28.2486	28.466	28.3502	28.3828
	Q9I385	PA1640	7	27.2	37.61	27.053	3.48E+08	24	345	24.1409	24.0212	24.1689	24.381	24.3638	24.2864	24.3033	24.3486
	Q9I398	PA1624	9	42.9	29.533	81.886	1.73E+09	50	268	26.0357	26.3627	26.458	26.1588	26.4333	26.2627	26.4655	26.2549
	Q9I399	PA1623	9	46.8	24.924	43.637	2.98E+09	34	220	27.2425	27.4125	27.5524	27.4357	27.8851	27.6007	27.598	27.3201
	Q9I3A6	PA1616	4	45.5	16.747	35.418	3.89E+08	15	154	24.4118	22.7683	24.0965	25.2519	24.6882	25.3441	24.5286	25.2232
ate dehydr	Q9I3A8	gpsA	13	55.3	36.773	86.681	1.24E+09	65	340	25.6069	25.1828	25.4671	25.2587	25.3663	25.6427	25.5895	25.7541
	Q9I3B2	fabB	19	62.7	42.806	323.31	7.94E+10	302	405	31.082	31.0013	31.2614	30.9134	30.7857	31.0274	31.0612	31.0075
	Q9I3B3	PA1608	7	18.9	58.237	41.885	2.1E+08	22	541	23.5005	23.8083	24.3871	24.4925	23.7635	22.2202	21.4758	23.8771
	Q9I3B9	PA1602	8	69.3	17.478	73.469	8.68E+08	33	163	25.8393	25.3919	23.6568	25.9213	25.5558	25.4107	25.5125	25.2601
	Q9I3C0	PA1601	15	33.6	81.329	93.584	2.54E+09	88	748	26.4476	26.5216	26.0903	26.4488	26.5786	26.3906	26.1871	26.1814
	Q9I3C4	PA1597	8	60.2	25.791	36.341	1.46E+09	40	241	27.115	25.6797	25.3958	27.0604	25.4753	26.2052	24.2256	25.9801
rone protei	Q9I3C5	htpG	51	76.3	71.667	323.31	1.47E+11	634	634	30.22	30.3245	30.4961	29.9781	30.2342	30.299	30.3554	30.3807
	Q9I3C8	PA1593	4	42	16.817	18.196	4.7E+08	15	157	25.0273	23.044	22.755	25.1133	25.3255	25.4219	25.2959	25.4421
poyl dehyd	Q9I3D1	lpdG	24	63.2	50.164	309.46	5.07E+10	257	478	29.8483	30.109	29.9773	29.6077	29.8824	30.094	29.3759	29.5155
e compone	Q9I3D2	sucB	34	83.4	42.887	323.31	1.79E+11	538	409	31.362	31.27	31.5277	31.0321	31.1856	31.422	31.4748	31.403
	Q9I3D4	sdhB	15	77.4	26.155	137.96	4.49E+10	149	235	30.7692	30.7219	30.1315	30.3715	30.3654	30.7414	30.3813	30.6284
	Q9I3D5	sdhA	30	59.2	63.531	323.31	5.57E+10	298	590	29.8488	29.9753	29.5902	29.571	29.8681	30.1538	29.8427	30.181
erized prot	Q9I3D8	PA1579	13	67.3	22.11	102.88	1.43E+10	123	202	28.193	28.5956	28.006	28.2931	28.3358	28.0244	28.6247	28.1013
	Q9I3E1	PA1576	4	25	30.777	27.954	2.05E+08	17	288	24.1585	24.5563	24.388	25.2146	24.2197	24.3544	23.3888	24.1092
45 protein I	Q9I3E3	PA1574	3	48.4	10.156	35.314	6.06E+09	32	93	28.1088	27.9983	27.3588	28.1919	27.9747	28.3303	28.0903	28.1437
	Q9I3E5	PA1572	4	19.7	40.701	15.532	1.96E+08	14	381	23.7711	22.4094	23.0594	23.7384	23.5946	23.6262	23.9044	24.1996
	Q9I3F2	PA1565	7	24.5	48.176	83.744	1E+09	41	436	25.3947	25.8765	25.1401	25.3995	25.2612	25.3065	25.1642	25.8219
sferase Tus	Q9I3F3	tusA	2	43	8.8652	26.669	1.39E+09	14	79	27.7045	26.9927	22.41	27.7836	22.8452	27.5851	21.6893	27.6474
tate hydrat	Q9I3F5	acnA	23	44.1	99.147	215.38	5.8E+09	170	910	26.4505	26.7757	26.721	26.4217	26.6144	26.5117	26.7354	27.0023
	Q9I3F6	aer	10	34	57.179	53.538	4.59E+08	33	521	23.7823	24.7777	23.7801	24.2372	24.3606	23.6261	24.0074	24.1936
	Q9I3G4	ccoO1	6	40.4	22.758	32.573	9.61E+08	22	203	25.3712	25.2012	24.9392	26.1523	25.6716	25.5994	25.6125	25.9615
chrome c c	Q9I3G5	ccoP1	11	54.4	34.644	65.833	2.64E+09	67	318	26.3282	26.7753	25.6234	26.9705	26.7011	26.5103	26.3224	26.9002
	Q9I3G7	PA1550	5	44.7	20.338	40.44	3.11E+08	20	179	23.6891	24.2066	23.6721	24.1558	24.1054	24.3213	24.4984	24.7678
nation prot	Q9I3H9	recR	6	64.6	21.173	59.528	2.2E+09	26	198	27.1484	23.1984	27.0844	28.0629	27.3005	27.1066	27.4765	27.4749
ociated pro	Q9I3I0	PA1533	2	38	11.877	124.15	4.81E+09	50	108	28.7119	28.3304	27.1454	28.7708	28.3287	28.9863	28.3941	28.8973
	Q9I3I1	dnaX	15	31.7	73.309	75.05	1.78E+09	77	681	25.3522	25.4173	25.243	24.8864	25.4834	25.5385	25.5258	25.9729
DNA ligase	Q9I3I4	ligA	25	40.4	86.767	154.47	3.09E+09	118	794	25.7431	25.8289	25.5251	25.4021	25.9503	26.0591	26.1671	26.2099
	Q9I3I7	PA1526	9	53	25.07	30.223	1.13E+09	55	219	25.7612	25.4347	25.6582	25.6447	25.7754	25.5982	25.6922	25.664
	Q9I3J2	PA1521	14	49.5	48.311	110.14	2.21E+09	97	434	25.6681	25.7794	26.0129	25.6543	26.1019	26.0111	25.9692	25.6112
	Q9I3J3	PA1520	14	69.9	28.646	105.56	7.88E+09	115	259	27.5278	27.8887	28.049	27.4455	27.3698	27.4383	27.3241	27.3777
yisourate I	Q9I3J5	PA1518	4	68.3	13.974	49.297	1.3E+09	24	126	26.6088	26.1901	25.8724	26.4335	25.1538	26.0042	25.702	26.1613
	Q9I3J7	PA1516	8	64.3	18.896	64.387	1.18E+09	35	171	26.3673	26.2663	25.5593	26.3088	25.6972	25.8372	25.7831	25.8174
loglycolate	Q9I3J9	allA	5	52.7	18.982	25.73	3.48E+08	22	169	24.5906	24.2278	22.3879	24.3564	24.8648	24.8834	24.4804	24.8956
n monopho	Q9I3K7	moaA2	5	20.2	37.214	10.757	9.45E+08	21	331	26.4098	26.6925	26.8397	26.8576	26.513	26.291	26.6796	26.5015

	Q9I3K8	PA1504	5	31	24.179	21.556	3E+08	13	216	24.293	24.5967	24.5134	23.6687	24.5598	23.8824	24.1926	24.4329
pyruvate is	Q9I3L1	PA1501	4	27.7	29.203	4.4535	86237000	15	260	23.8058	23.1161	23.4936	24.1089	23.2818	23.4504	22.8969	23.5341
	Q9I3L8	PA1494	21	50.3	60.65	156.75	4.14E+09	112	551	26.1124	26.1685	26.4059	26.3697	26.4065	26.1813	26.9909	26.0447
	Q9I3L9	cysP	12	50.9	36.517	110.04	1.69E+09	76	332	25.7083	25.3836	22.8155	25.7582	26.123	25.789	24.015	25.5947
s ATP-bindi	Q9I3N7	ccmA	6	39.5	24.995	72.111	8.21E+08	37	233	24.9798	25.027	24.4601	24.803	25.2933	25.1131	25.0752	25.3772
	Q9I3N8	PA1474	10	42.3	52.688	71.004	4.64E+08	49	492	25.1181	24.5594	24.9839	24.0464	23.5919	24.5373	24.2997	24.5628
	Q9I3Q7	PA1440	5	51.8	22.382	61.935	5.84E+09	57	195	28.7281	28.1593	28.5463	28.8119	28.4836	28.47	28.2763	28.6698
	Q9I3S7	PA1417	11	39.2	57.29	36.609	6.01E+08	34	533	24.4042	23.9764	24.5784	24.3898	24.6147	24.6031	24.2148	24.5974
	Q9I3T4	PA1410	8	32	40.451	27.164	4.88E+08	32	363	24.8465	24.7507	24.937	24.6003	24.6822	24.8224	25.028	25.0097
	Q9I3T5	aphA	10	51.2	37.953	56.679	8.44E+08	35	346	24.8274	24.9948	24.7743	24.8543	25.3613	25.3555	25.0107	25.2341
	Q9I3U2	PA1402	8	53.4	23.753	27.311	1.06E+09	36	221	26.5189	26.47	26.5881	26.4399	26.1585	26.3937	26.8765	26.0479
phosphate	Q9I3W9	pdxB	7	26.6	41.001	35.805	1.32E+09	40	380	25.5914	25.6851	25.8743	25.9463	26.2538	26.0736	26.2519	25.8396
carrier-pro	Q9I3X1	fabF2	8	34.6	43.485	27.118	4.39E+08	25	422	25.3005	24.8237	25.1523	24.4212	25.0344	24.7343	24.9588	25.0457
	Q9I400	PA1344	11	48.1	27.397	81.45	2.52E+09	51	264	26.2632	26.7551	26.9866	26.2448	26.5437	26.3536	26.9504	26.2117
	Q9I405	1339;PA41	10	52.9	26.916	47.219	1.83E+09	54	244	26.3073	26.4709	26.5613	26.0378	26.1472	26.0755	25.985	26.4598
peptidase	Q9I406	ggt	24	50.4	59.873	181.51	1.01E+10	168	557	28.111	27.9919	27.5444	28.1736	27.9468	27.9276	27.5404	27.8437
	Q9I437	PA1307	8	43.6	28.544	80.707	1.83E+09	45	257	26.6655	26.8087	26.7461	26.6621	26.8227	26.5224	26.6406	26.4057
50 protein I	Q9I445	PA1299	3	36.2	17.234	15.535	1.84E+08	14	149	24.0685	22.4678	23.9876	24.6201	24.1642	23.8425	23.1396	24.047
	Q9I446	PA1298	2	38.5	10.023	11.652	2.53E+08	12	91	24.8587	23.1192	25.0678	23.3329	24.304	24.1272	24.679	24.6585
ontaining p	Q9I449	PA1295	2	49.5	11.361	20.929	1.86E+09	17	97	27.2933	26.3401	25.4938	27.2527	26.572	27.3016	26.7222	27.6438
bonuclease	Q9I450	rnd	16	61.8	43.098	141.08	3.65E+09	90	374	25.8967	26.0097	26.0165	25.9933	26.1793	26.1139	26.4599	26.2059
	Q9I451	PA1293	5	28.6	38.794	24.365	1.77E+08	16	353	22.652	22.7405	24.0559	24.1665	24.1945	24.0017	24.1439	24.3111
otopyruvate	Q9I452	sseA	7	45.8	30.597	70.935	1.44E+09	36	284	26.807	26.5464	26.6409	26.5154	23.0418	26.1128	26.0031	26.2962
	Q9I461	PA1283	3	24.7	20.307	7.6533	2.51E+08	15	186	22.8064	24.7967	25.0167	24.7366	25.3664	25.2515	22.9477	23.0452
balamin bi	Q9I466	cobP	8	64.2	18.862	60.848	8.8E+08	35	173	24.6857	24.5306	24.6447	24.8831	24.9725	25.0559	24.9408	24.8582
:-diamide a	Q9I472	cobO	5	36.9	22.292	31.866	2.29E+09	27	203	26.5901	26.8924	26.9702	26.7191	26.9633	27.1385	27.1083	26.8937
	Q9I4A4	PA1234	3	33.5	18.46	13.724	5.56E+08	20	167	25.7486	25.4152	25.4853	25.7889	25.5318	23.4713	25.4957	25.4949
	Q9I4B2	PA1226	7	50	22.308	44.483	5.86E+08	40	202	24.4849	24.6965	24.3331	24.4839	24.3502	24.5092	24.4656	24.649
	Q9I4D2	PA1206	9	79.6	17.69	84.821	3.85E+09	73	157	26.5966	26.5777	26.0081	26.4695	26.4074	26.6262	26.5474	26.5598
:in 2,3-diox	Q9I4D3	PA1205	6	31.4	34.455	34.388	7.03E+08	30	315	25.8718	26.069	26.0157	26.1397	25.3683	25.4782	25.7404	23.8187
ent FMN re	Q9I4D4	PA1204	4	40.5	20.224	47.987	1.34E+09	21	185	26.2767	26.0395	26.4083	26.1724	26.1578	25.7421	25.6786	25.5395
	Q9I4D5	PA1203	3	26.7	14.681	13.362	5.86E+08	18	135	23.2713	25.4981	22.9827	26.2299	24.8758	25.6307	24.5437	25.5728
ent protei	Q9I4E1	cobB2	4	27	27.686	28.846	2.69E+08	17	256	24.3481	24.3075	23.8557	24.0624	22.7994	24.2912	24.1553	23.9847
ne biosynth	Q9I4E6	ttcA	6	27.7	31.26	30.696	7.89E+08	40	274	24.9029	24.8074	25.242	24.6537	25.1231	25.311	25.1032	25.4149
stidine kina	Q9I4F8	phoQ	4	12.7	50.28	17.185	84879000	15	448	23.1251	23.4832	23.6455	22.5679	23.2236	23.6534	23.1262	22.9372
	Q9I4F9	phoP	18	75.1	25.65	323.31	4.73E+10	216	225	29.7703	29.6675	29.9369	29.64	30.1879	30.2748	30.4041	30.1587
	Q9I4H3	PA1164	4	27.4	28.898	29.615	5.3E+08	21	270	25.1474	25.1756	25.1493	25.0452	24.9458	25.0257	22.3095	25.176
nopimelate	Q9I4H5	dapE	13	53.3	41.077	128.53	1.02E+10	119	383	27.5207	27.6707	27.5833	27.611	27.7507	27.8669	27.9872	27.7323
	Q9I4H6	rrmA	8	36.7	29.644	16.209	4.34E+08	20	267	24.5689	24.376	24.5666	24.3968	24.4717	24.3244	24.6733	24.3815
	Q9I4H7	PA1160	6	79.5	14.042	53.816	1.49E+09	34	127	26.5212	26.0957	25.6019	26.6893	26.2552	26.3656	25.9451	26.256
	Q9I4H8	PA1159	4	50.7	7.7246	40.226	2.12E+10	44	69	29.6866	29.727	29.4484	29.7408	29.9562	29.9839	30.1019	29.7582
osphate re	Q9I4I2	nrdB	20	69.9	47.386	252.44	1.54E+10	141	415	28.5579	28.5455	27.8782	28.9056	29.0811	28.9691	28.8284	28.8371
	Q9I4J5	PA1140	6	43.9	31.614	58.921	1.84E+09	43	278	25.7937	26.2375	25.9709	25.3868	26.1024	25.9624	25.9736	25.8734
	Q9I4J6	PA1139	4	32.6	29.336	28.53	4.16E+08	14	282	25.9375	23.9299	23.0807	25.8824	22.287	24.8363	23.809	24.5616
	Q9I4K8	PA1127	22	76.6	36.474	275.62	2.3E+10	202	329	29.4182	29.5193	29.8739	29.6292	29.4535	29.2982	29.5299	28.9969
	Q9I4L8	PA1117	5	72.6	13.589	20.361	2.46E+08	21	124	23.8236	23.7692	23.2204	24.347	24.1885	24.2483	23.6431	23.7296
	Q9I4L9	PA1116	6	29	31.308	23.884	3.84E+08	20	279	24.6928	24.2552	24.9419	24.9581	24.8207	25.0196	25.0092	24.5939
specific AT	Q9I4N1	fliI	11	36.4	48.6	33.706	3.6E+08	25	451	22.5848	24.5393	24.6537	24.4154	24.732	24.7307	23.5937	24.1806
	Q9I4N3	fleR	17	51.8	51.234	83.763	1.93E+09	83	473	25.7942	25.8224	26.0961	25.6743	25.8134	25.9552	25.891	25.9083
lar P-ring p	Q9I4P5	flgI	14	57.2	38.18	72.387	2.26E+09	78	369	25.5801	25.6291	25.4228	25.7382	25.4432	25.7118	25.4105	25.6837
	Q9I4P9	flgE	9	50.4	48.335	113.93	1.44E+09	55	462	25.4602	25.6532	25.3303	25.9012	25.9917	25.9397	26.1108	25.9392
	Q9I4Q0	flgD	4	38.8	24.85	42.265	4.53E+08	25	237	25.1871	25.0089	23.844	25.1911	25.2177	24.7987	25.2215	25.2025
	Q9I4Q4	PA1075	3	58.1	11.337	17.359	5.76E+08	25	105	25.6107	23.2683	23.7947	26.2314	24.4008	25.7601	24.9137	25.5242
	Q9I4Q5	PA1069	12	25.4	86.114	35.952	3.75E+08	37	764	23.6312	23.9046	24.377	24.1056	24.9862	24.3296	24.7095	24.2127
	Q9I4R2	PA1062	5	38.2	19.155	31.771	4.6E+08	22	173	24.5813	24.7448	22.8429	24.5488	24.7195	24.5352	24.9261	24.4312
	Q9I4R3	PA1061	11	55.9	39.952	40.886	4.88E+08	38	365	23.5807	23.5423	23.2701	24.6021	24.6461	24.5874	24.1219	24.2189
	Q9I4S1	PA1053	5	39.6	15.649	91.019	2.34E+09	37	154	26.0219	26.4124	25.4205	26.6047	26.676	26.6048	26.3716	26.7857
	Q9I4S2	PA1052	9	39.6	38.62	75.037	9.19E+08	36	381	25.0176	25.0472	24.9528	22.7593	25.9307	25.392	25.8041	25.7162
	Q9I4T1	PA1043	10	53.5	27.275	60.146	2.15E+09	60	269	26.3974	26.3964	26.4932	26.2416	26.0179	25.6789	25.938	25.9572
	Q9I4T8	PA1036	5	38.2	19.537	30.344	8.72E+08	40	186	25.5942	25.4815	25.1295	25.7635	24.9091	25.2159	24.8817	24.8453
	Q9I4V8	PA1015	7	38	28.853	33.823	5.83E+08	30	266	24.7981	24.9213	25.1486	24.5946	24.6195	24.5205	25.2397	24.8427
dazole-succ	Q9I4W0	purC	17	74.6	26.83	117.28	2.71E+10	123	236	29.1395	29.4491	29.3648	29.1574	29.1262	29.1859	29.2358	29.2194
hydrodipic	Q9I4W3	dapA	16	67.8	31.449	222.59	4.31E+10	163	292	30.0166	29.9864	29.6393	30.0301	30.0537	30.0908	29.9269	29.9602
	Q9I4W5	bcp	10	78.3	17.352	44.634	1.09E+10	76	157	29.3017	29.4037	28.9067	29.2626	29.4324	29.3067	29.3689	29.1607
	Q9I4W7	PA1006	2	57.8	9.1423	21.569	3.77E+08	18	83	24.7047	24.0027	23.3174	24.8597	23.9072	24.589	24.2116	24.9287
assembly-	Q9I4W8	PA1005	15	38.4	52.994	142.43	2.29E+09	77	477	25.6892	25.9691	25.8992	25.8019	25.8517	25.6771	26.0171	25.7088
linate syntl	Q9I4W9	nadA	11	62.5	38.418	121.89	6.34E+09	112	352	27.531	27.3652	27.3649	27.4896	27.5376	27.8312	27.6847	27.6587
leazaguani	Q9I4Z2	queC	5	45.5	23.9	37.255	9.8E+08	24	224	25.989	26.0006	25.2507	25.7927	26.2013	25.888	26.1016	25.8647
deazaguan	Q9I4Z3	queE	6	23.9	29.556	17.979	4.41E+08	20	264	24.6439	23.8829	23.9895	24.8167	24.6799	24.5726	24.0376	24.4837
	Q9I4Z5	PA0968	2	31.1	16.672	23.108	1.08E+09	18	148	24.536	22.2943	27.2268	26.5013	27.0974	26.7425	27.2702	26.6451
in SlyX hor	Q9I4Z9	slyX	2	46.4	7.8206	9.6929	3.85E+08	15	69	25.6019	23.8624	23.4461	25.5625	23.9454	25.2879	23.0674	24.7965
ne--tRNA li	Q9I502	proS	30	72.3	63.103	323.31	2.56E+10	214	571	28.6355	28.5589	28.597	28.4194	28.986	28.9877	28.9028	28.9032
ylphosphat	Q9I504	acyP	4	64.8	10.313	38.267	5.73E+08	17	91	25.7799	25.6065	23.0019	25.5549	25.5628	25.9129	25.8563	25.6018
nate reduc	Q9I508	PA0950	6	59	12.79	54.565	4.3E+09	55	117	27.8076	26.8823	26.221	27.746	27.1562	27.6854	26.7878	27.4895
hydrogena:	Q9I509	PA0949	7	46.5	20.762	43.246	1.88E+09	43	198	26.8542	26.6524	26.3757	26.528	26.5136	26.6865	26.5455	26.6524
	Q9I511	PA0947	9	46.2	26.843	31.507	7.96E+08	43	234	24.7696	25.0791	25.2682	25.0609	25.1597	25.2896	25.2145	25.3007
	Q9I512	PA0946	9	55.7	36.776	208.36	9.58E+09	96	345	28.307	28.2199	27.7631	28.3919	28.417	28.3169	28.4215	28.1125
mylglycinai	Q9I513	purM	14	50.7	37.122	212.81	3.36E+10	145	353	29.7827	29.7154	29.4984	29.7686	29.6831	29.914	29.814	29.781
ycinamide f	Q9I514	purN	6	46.8	24.734	43.165	1.18E+09	36	222	26.0626	25.9075	26.0419	25.9314	25.9596	25.8834	26.0408	26.1183
	Q9I515	PA0943	14	52.1	27.183	80.575	6.28E+09	69	238	27.6091	27.8969	28.1335	26.8832	27.2975	27.7424	28.4425	27.47
	Q9I516	PA0942	3	23.1	20.622	8.8753	3.58E+08	13	186	24.5779	24.6599	25.0394	24.9505	21.6708	24.7579	24.8438	24.2156
	Q9I517	PA0941	4	62.3	8.2845	18.518	8.83E+08	28	77	26.4986	25.9477	25.2368	26.2795	24.5446	25.8022	25.309	25.4016
	Q9I521	PA0937	8	38.5	20.789	42.992	4.79E+09	53	179	26.9505	27.0869	27.0284	27.0674	27.3655	27.4092	27.7099	27.3313
	Q9I523	PA0935	7	35.5	31.25	32.75	1.41E+09	20	276	26.7827	26.8908	26.8403	27.1307	27.0384	27.2404	27.3468	22.8741
3)-C(5))-me	Q9I525	rlmD	5	23.1	49.07	18.399	1.01E+09	14	450	23.8911	26.7468	23.4403	27.0259	27.2449	26.8555	27.1477	24.0083
eine syntha	Q9I526	cysM	21	68.6	32.448	259.47	1.18E+10	182	299	27.6539	27.5265	26.7722	27.6885	27.929	27.8787	27.2134	27.8132
	Q9I531	PA0926	5	46.8	15.829	40.473	1.44E+09	33	141	25.9898	25.8791	25.3689	26.1035	25.8912	26.0422	26.1578	26.036
12 methylt	Q9I541	rimO	16	58.4	48.96	109.42	2.78E+09	88	440	26.2445	26.2498	26.0529	26.1141	26.267	26.5193	25.7013	26.5339
m transpo	Q9I544	mgtE	6	23.7	53.82	26.959	2.93E+08	24	482	23.4992	24.2214	23.9427	23.9616	24.6967	24.1777	23.4945	24.7493
	Q9I578	PA0863	8	38.9	33.368	44.451	8.35E+08	32	319	24.9605	25.197	25.2942	25.1622	24.8325	24.6439	24.7164	24.4411
	Q9I582	PA0859	4	37	21.466	49.805	3.41E+08	25	200	24.4397	24.236	23.7186	24.5847	24.3549	24.3341	24.1415	24.7446
	Q9I584	bolA	4	76.2	11.179	41.08	5.72E+09	30	101	27.9458	27.5428	26.5905	28.2209	28.1403	28.426	27.9395	28.3722
	Q9I585	PA0856	9	45.6	19.998	124.61	3.37E+10	102	182	30.1727	30.0719	29.3849	30.3712	30.5257	30.2947	30.4259	30.4797
	Q9I5A0	PA0840	19	57.3	40.333	111.64	5.62E+09	114	370	27.2278	27.2483	27.3892	27.3116	27.0735	26.8847	27.1195	26.6978
hione pero	Q9I5A2	PA0838	7	81.9	17.263	41.114	2.94E+09	36	160	28.4026	27.6971	27.4416	28.3271	27.2223	28.1494	27.5014	27.942
	Q9I5A8	PA0832	10	73.7	23.482	81.664	1.04E+09	28	213	25.6452	24.1974	24.7536	25.8654	24.5806	25.598	24.504	25.621
	Q9I5D4	PA0804	6	39.2	30.017	45.846	4.45E+08	28	273	24.577	24.0101	25.0338	24.5163	24.4517	24.1292	24.7867	24.3054
ylisocitrate	Q9I5E2	prpB	14	65.1	32.137	119.11	3.17E+09	107	298	26.2968	26.4873	26.6713	26.0672	26.0165	26.0704	25.9976	25.9748
rate syntha	Q9I5E3	prpC	11	43.2	41.689	81.573	1.55E+09	68	375	25.3767	25.4856	25.5712	25.7584	25.2491	25.4091	25.3804	25.5398
	Q9I5E4	PA0794	18	34.8	94.89	51.832	1.02E+09	50	868	25.187	25.2392	25.1821	25.3459	25.1325	25.3141	25.1895	25.2142
	Q9I5E5	PA0793	9	38.7	41.709	44.083	1.02E+09	40	395	25.5714	26.0222	26.0437	25.3769	25.7093	25.6729	25.7227	25.4802
	Q9I5F6	putA	55	62.5	115.63	323.31	2.58E+10	330	1060	28.3405	28.7889	28.1944	28.489	28.8726	28.8763	28.6408	29.196
on proteas	Q9I5F9	lon	30	44.6	88.574	231.03	9.63E+09	220	799	27.0837	27.147	27.1357	26.8882	27.2728	27.4565	27.3051	27.2981
J34)-methy	Q9I5G3	cmoA	5	25.2	27.64	28.235	1.5E+09	39	246	25.3796	25.5364	25.5493	25.0028	25.7653	25.8299	25.6375	25.8239
5'-phospha	Q9I5G5	pdxJ	6	36.7	27.26	83.964	9.16E+08	23	248	24.9616	25.2652	24.4267	25.7941	26.1391	25.7309	25.713	25.7095
nal peptida	Q9I5G7	lepB	6	27.1	32.103	13.713	2.36E+08	13	284	23.9134	22.5075	24.5377	22.3948	24.7488	24.303	23.4439	24.795
gation fact	Q9I5G8	lepA	19	42.2	66.294	177.13	4.74E+09	104	599	26.849	27.0313	26.5203	26.6906	26.7186	27.0017	26.6432	27.237
erized prot	Q9I5G9	PA0760	2	20.2	10.147	21.969	1.72E+08	9	84	24.4002	22.1813	23.5915	24.5884	24.2467	23.6789	24.0733	24.1289
	Q9I5H0	PA0759	13	63.1	33.667	267.21	6.75E+09	89	314	27.6879	27.7915	28.0875	27.6302	27.6784	27.6676	27.7717	27.6477
	Q9I5H1	PA0758	12	59.4	30.875	112.02	7.79E+09	93	278	28.1045	28.0998	27.5038	28.1475	28.0162	28.1595	27.7638	28.1306
-DNA glyco	Q9I5H9	ung	5	37.2	26.009	35.546	1.56E+09	34	231	26.6794	26.5588	26.7646	26.5138	26.4474	26.6356	26.4812	26.5411
	Q9I5I3	PA0746	5	22.7	42.553	29.434	2.31E+08	19	387	23.7006	24.0717	24.5483	23.0461	23.9014	22.801	23.9187	23.0048
	Q9I5I4	PA0745	15	52.9	29.904	83.847	2.52E+09	49	272	26.7975	26.8618	25.4286	26.5579	26.6256	26.1613	26.3235	26.0848
	Q9I5I5	PA0744	4	17.2	40.589	17.603	1.7E+08	18	367	24.8045	23.0325	23.9268	24.3665	23.2998	23.7728	22.7518	22.996
	Q9I5J9	PA0729	5	37.4	12.985	18.643	1.1E+09	25	115	26.6863	26.6017	25.6801	26.638	26.1238	26.8238	26.8241	26.6995
ne--tRNA li	Q9I5Q3	tyrS2	22	66.4	44.129	197.71	2.2E+10	179	399	29.0296	28.9248	29.2184	28.9357	29.1811	29.1096	29.1554	29.1175
etylmuram	Q9I5Q5	anmK	6	29.8	39.119	13.815	4.85E+08	19	363	23.2299	25.1196	24.7797	23.639	23.3399	25.0603	25.0682	25.2748
ter insertic	Q9I5Q6	erpA	5	68.1	12.484	49.473	2.95E+09	46	116	28.2988	27.3062	26.6806	28.1895	27.5806	28.0669	27.4703	28.0611
	Q9I5Q7	PA0664	7	74.3	14.822	59.715	4.69E+09	64	140	27.3558	27.2779	27.1067	27.3847	27.2564	27.083	27.2347	27.1636
lutamyl-ph	Q9I5Q9	argC	10	44.5	36.684	36.871	7.96E+08	47	344	24.7894	24.6927	23.3043	24.4208	24.5145	25.3281	24.4715	24.9692
	Q9I5R1	PA0660	10	39.8	34.814	66.534	1.71E+09	61	322	25.7435	25.7491	26.05	25.4929	25.423	25.6558	25.7736	25.6195
nine decarl	Q9I5R7	speD	11	43.2	30.478	147.01	1.48E+10	101	264	28.7194	28.6625	27.448	28.4833	28.8761	29.0623	28.6423	28.8454
	Q9I5S9	PA0623	7	61.1	17.939	28.615	6.52E+08	22	167	25.4054	25.2569	26.1241	25.5718	22.2646	24.9309	24.858	24.0655
nosphate 3	Q9I5T1	rpe	14	81.7	24.095	147.69	1.22E+10	116	224	28.1738	27.8614	25.6989	28.0472	28.7443	28.747	27.8698	28.7993

	Q9I5T7	PA0601	7	55.7	22.9	25.107	3.07E+08	21	210	23.9538	24.454	24.4723	24.1725	23.5834	24.0023	23.35	23.931
	Q9I5T9	PA0599	3	14.2	37.979	15.382	4.83E+08	16	353	25.4032	25.7658	22.6316	23.1989	23.6117	25.4332	25.1656	25.4255
	Q9I5U0	PA0597	3	36.6	24.149	35.129	2.53E+09	43	224	27.0747	26.6779	26.014	26.8918	26.5317	26.689	26.5262	26.955
	Q9I5U1	PA0596	13	50	38.185	127.78	7.78E+09	112	338	27.0854	27.092	27.1293	27.4319	27.2232	27.1088	27.3256	26.9937
aperone Su	Q9I5U3	surA	28	71.7	46.953	323.31	3.92E+10	288	417	29.3845	29.444	28.5672	29.4552	29.5226	29.442	29.5494	29.4393
-4-phospha	Q9I5U4	pdxA1	7	43	34.923	46.967	9.13E+08	40	328	25.3236	25.4265	25.5736	25.1847	24.8648	25.4031	25.8979	25.4795
ll subunit r	Q9I5U5	rsmA	7	45.5	30.058	61.528	1.84E+09	54	268	26.0928	26.0087	26.1609	26.1005	25.8885	26.3579	26.3285	26.3116
rotein Apa	Q9I5U6	apaG	3	48.4	13.622	51.355	2.19E+09	32	126	27.993	27.2791	26.738	27.9811	27.9372	27.4968	27.3626	27.9161
etraphosph	Q9I5U7	apaH	14	61.8	32.018	61.108	1.83E+09	64	283	25.7243	25.9207	25.876	25.7179	25.7571	25.6452	25.7518	25.7096
CA-adding e	Q9I5V3	cca	10	36.1	45.639	58.179	4.41E+08	41	410	24.3976	24.211	24.5281	24.7035	23.9086	23.7382	24.0386	23.9612
	Q9I5V4	PA0583	7	53.3	20.091	30.596	3.97E+08	19	180	24.2392	23.8186	23.8908	24.5364	23.2581	24.559	22.8166	24.3455
threonylca	Q9I5V7	tsaD	6	28.7	36.561	40.475	5.52E+08	28	341	24.2636	24.6684	24.3018	24.2801	24.715	24.7058	24.9475	24.7853
osomal pro	Q9I5V8	rpsU	6	46.5	8.4848	48.034	1.71E+10	83	71	29.1701	29.5367	28.8689	29.5754	30.1347	29.8021	29.5643	29.9246
	Q9I5V9	PA0578	3	22.8	16.842	20.136	8.68E+08	23	149	25.4743	25.9632	24.7388	25.6038	25.9275	25.8397	25.9801	25.8
DNA primas	Q9I5W0	dnaG	8	19.6	74.175	25.353	1.29E+08	20	664	22.5641	23.7606	23.8966	22.353	23.929	24.0199	23.8631	23.4807
	Q9I5X4	PA0562	8	45.5	24.232	97.125	1.66E+09	56	224	25.8426	25.9703	25.9217	25.6896	26.0551	25.8671	26.0177	25.7664
	Q9I5X7	PA0559	7	21.4	42.618	35.682	9.37E+08	37	392	25.1952	25.2128	25.1439	25.4514	25.5251	25.2247	25.4015	25.0739
isphosphat	Q9I5Y1	fba	24	64.7	38.573	323.31	8.36E+10	323	354	30.4805	30.7633	30.6889	30.3853	30.7102	30.8141	30.722	30.6466
oglycerate	Q9I5Y4	pgk	20	69.3	40.404	278.67	6.49E+10	258	387	29.6175	29.7232	29.8875	29.6972	30.0733	30.0287	30.1822	29.895
phosphate	Q9I5Y5	epd	11	62	38.743	139.42	1.97E+09	62	353	25.8173	25.6735	25.63	25.8016	26.2859	26.2606	26.5172	26.1647
pyruvate is	Q9I5Y6	PA0550	11	57	29.834	54.922	9.04E+08	40	265	24.9108	24.8918	24.9344	24.9071	24.8367	24.6216	24.6899	24.6121
ransketolas	Q9I5Y8	tktA	35	73.1	72.219	323.31	2.12E+10	262	665	27.7123	27.9528	27.2295	28.3417	28.5504	28.4608	28.1385	28.3515
lmethionin	Q9I5Z0	metK	27	85.4	42.709	299.53	1.15E+11	361	396	30.9925	31.01	31.082	30.8672	31.1995	31.1226	31.0723	30.9536
	Q9I5Z4	PA0542	4	23	14.767	85.396	4.72E+09	37	139	27.9586	28.464	28.8915	28.2546	28.0576	27.5297	28.5342	27.949
	Q9I610	8;PA4199;F	13	34.6	63.628	48.913	2.19E+09	66	592	26.2556	26.3857	25.981	26.1987	26.2988	26.2531	26.3783	25.9484
	Q9I612	PA0506	23	50.7	65.358	219.55	9.06E+09	140	601	28.1562	28.1567	27.7983	27.9482	27.967	28.0016	27.8789	28.0353
ethiobiotir	Q9I614	bioD	4	28.9	23.337	23.965	5.39E+08	25	228	25.6994	25.589	25.2681	25.307	25.4918	25.6802	25.2259	25.5334
	Q9I616	PA0502	5	24.6	26.494	53.148	6.91E+08	28	240	25.4377	24.3916	24.8204	25.0895	25.2224	25.0725	24.588	24.871
oxononanoa	Q9I617	bioF	10	50.6	42.237	68.691	4.96E+08	36	401	24.9458	23.5966	24.3677	24.8134	24.6169	24.7409	24.6488	25.2326
otin syntha	Q9I618	bioB	21	71.3	39.113	270.04	1.17E+10	167	352	27.9853	28.0508	28.1502	28.0292	28.5399	28.3476	28.4956	28.3669
	Q9I622	PA0496	12	59.7	34.635	143.3	2.03E+09	68	325	25.75	25.7871	26.4475	25.4042	25.2281	25.0312	25.5542	25.2141
	Q9I623	PA0495	3	18.5	32.951	15.295	3.43E+08	15	292	23.8993	24.9864	25.1751	24.3635	22.1316	22.7538	24.8713	24.8075
	Q9I624	PA0494	10	40.8	50.07	46.89	3.31E+08	35	458	23.3295	24.0817	25.0819	22.3689	24.0172	24.0949	23.5677	23.8214
71 protein I	Q9I626	PA0492	8	50	27.559	36.002	7.7E+08	47	252	25.0646	24.9575	25.0655	24.8615	25.1128	24.8032	25.145	24.6681
	Q9I631	PA0487	6	29.4	26.948	29.108	7.28E+08	25	252	25.2899	25.3045	25.8382	25.5514	24.855	25.2617	25.3354	25.3065
	Q9I632	PA0486	3	16	36.968	16.574	1.91E+08	17	324	24.0603	23.7203	23.832	23.7654	23.6749	23.8502	23.9296	24.0529
ate syntha:	Q9I636	glcB	31	64.4	78.659	323.31	3.16E+10	261	725	29.3333	29.4352	29.5647	29.296	29.5737	29.4084	29.4751	29.2839
	Q9I645	PA0473	6	39	28.326	27.539	7.54E+08	29	249	25.6699	25.9871	25.9129	25.6929	25.842	25.9633	25.8549	25.6548
	Q9I649	PA0469	19	68.4	32.137	224.26	4.65E+09	107	285	26.4297	26.5332	26.7242	26.3961	26.0401	26.0596	26.3013	25.9234
	Q9I650	PA0468	9	53.8	33.594	121.29	2.65E+09	71	316	26.5244	26.1227	25.6672	26.7849	25.666	25.7414	25.2484	26.0738
	Q9I651	PA0467	9	61.2	23.301	64.532	9.39E+08	38	206	25.3171	25.5838	25.3304	25.6474	25.5463	25.3695	25.2422	25.2407
	Q9I655	creB	7	38.9	25.573	61.837	7.43E+08	30	229	25.2341	25.3756	25.4581	25.329	24.9867	25.1372	25.0354	24.9038
	Q9I656	PA0462	5	21.8	26.262	15.538	9.71E+08	19	234	23.2085	25.9894	25.748	25.8279	26.6683	26.0499	26.6808	26.2991
	Q9I658	PA0460	8	77.6	18.727	69.193	1.14E+10	80	192	28.7929	27.7093	27.2045	28.1478	27.7364	29.3726	28.6938	28.9425
	Q9I662	PA0456	7	82.6	7.7066	82.635	2.36E+11	100	69	34.6019	34.1945	33.1272	34.57	34.0861	34.3375	34.2926	34.167
	Q9I663	dbpA	6	24	49.784	30.143	3.27E+08	19	458	24.7385	24.3657	24.5446	24.3558	24.1192	23.9379	22.4701	23.9501
ylhomocys	Q9I685	ahcY	35	80.6	51.399	323.31	9.56E+10	390	469	30.4843	30.6287	30.6476	30.3925	30.3453	30.4495	30.4996	30.4124
	Q9I686	PA0431	5	63.6	20.639	16.905	4.74E+08	36	184	24.8322	24.631	23.9072	25.0595	25.1711	24.9477	24.6649	24.9196
rahydrofol	Q9I687	metF	16	66.6	32.228	132	1.52E+10	142	290	28.9474	29.0653	29.0519	28.8248	28.7964	28.9832	28.8641	28.7722
dent RNA h	Q9I689	rhlE	26	49.9	70.111	220.45	6.77E+09	166	639	26.6285	27.1533	26.4339	27.155	27.3776	27.081	26.8627	27.3612
	Q9I692	PA0421	26	63.1	54.207	285.88	6.48E+09	155	496	27.1694	27.3133	27.4701	27.1297	27.1251	27.2311	27.2752	27.2587
no-7-oxonc	Q9I693	bioA	19	59.3	52.477	83.102	2.31E+09	90	467	26.2009	26.2426	26.0621	26.179	25.7187	25.6037	25.6167	25.6967
ll subunit r	Q9I694	PA0419	6	42.1	26.468	108.57	2.26E+09	51	240	27.0447	27.1152	27.0057	26.9796	26.9283	27.0199	26.7948	26.9378
	Q9I695	PA0418	7	25.1	52.172	23.636	3.94E+08	33	471	24.6252	24.3367	24.6118	24.5772	24.5302	24.3861	24.755	24.4482
	Q9I696	chpA	39	24.5	268.57	199.36	1.87E+09	123	2472	24.8912	25.451	24.6655	25.4916	25.0323	24.7949	24.4237	24.9984
hione synt	Q9I697	gshB	18	70.3	35.705	233.49	1.04E+10	129	317	27.2793	27.457	27.644	27.1404	27.6201	27.4114	27.696	27.2386
	Q9I6A0	PA0400	10	49.7	42.786	132.52	4.86E+09	81	394	27.4081	27.1052	27.6863	27.06	27.2925	27.3298	27.5219	27.5042
	Q9I6A1	PA0399	14	52.1	49.17	145.23	1.77E+09	77	457	25.846	25.7426	26.1384	25.3219	25.0892	25.4097	25.6014	25.4919
	Q9I6A6	PA0389	5	42.2	23.773	31.717	4.45E+08	29	206	24.4707	24.1198	23.8481	24.7337	24.1676	24.4509	24.7436	25.1638
	Q9I6A7	PA0388	10	69.8	15.302	128.83	1.38E+10	91	139	29.0394	28.9407	28.4262	29.2595	28.9708	28.8851	28.7822	28.6886
urine NTP p	Q9I6A8	PA0387	11	76.6	21.242	114.52	8.99E+09	118	197	27.6902	27.5697	27.1018	27.8218	27.5198	27.6779	27.5319	27.6026
-N(7)-)-met	Q9I6B3	trmB	9	50.4	27.595	73.335	3.34E+09	78	244	26.4804	26.6702	26.6905	26.4756	26.4848	26.7236	26.6725	26.6319
azole synth	Q9I6B4	thiG	16	66	28.234	204.35	1.39E+10	129	265	28.273	28.005	27.9857	28.1156	28.4512	28.5505	28.4687	28.3716
	Q9I6B5	PA0380	4	90.9	7.0579	24.762	6.63E+08	24	66	26.0142	23.2901	23.2348	25.7535	23.568	25.7791	24.0195	24.9915
	Q9I6C0	ftsE	6	41.3	24.612	51.01	1.68E+09	40	223	26.1901	26.3195	26.364	26.0848	26.3993	26.083	26.5543	26.0561
ion particle	Q9I6C1	ftsY	28	68.6	48.391	272.14	1.38E+10	185	455	27.8612	27.9928	28.0072	27.8971	28.0085	27.9182	27.9459	27.8508
	Q9I6C2	PA0372	28	71.8	51.975	323.31	1.53E+10	198	465	28.2076	28.3007	28.4902	28.1777	28.6785	28.6342	28.9039	28.5704
	Q9I6C3	PA0371	14	46.1	53.14	85.545	1.79E+09	87	495	25.7407	25.766	25.887	25.8422	25.891	25.8959	26.0864	25.9111
ll subunit r	Q9I6C4	PA0370	8	60.1	21.748	41.861	7.45E+08	43	198	24.7057	24.1691	24.8812	24.8643	24.7315	24.9085	25.108	25.0298
	Q9I6C7	PA0367	6	51.6	24.54	54.05	1.6E+09	43	215	26.2584	26.0052	26.3456	26.0278	26.0704	25.7355	26.2415	25.3386
yl aldehyde	Q9I6C8	calB	18	50	52.875	124.84	2.63E+09	83	476	26.0504	26.3542	26.6188	26.234	25.7164	25.9293	25.8811	25.8938
heine ader	Q9I6D1	coaD	9	76.1	17.771	102.22	7.11E+09	90	159	27.3468	27.4096	27.4649	27.321	27.275	27.4549	27.2967	27.2894
	Q9I6D3	PA0361	9	23.7	62.061	46.629	8.04E+08	40	577	25.7442	25.7002	25.3386	25.6195	24.5922	25.1747	25.2227	25.1402
	Q9I6D7	PA0356	10	57.7	30.392	68.125	2.16E+09	63	274	26.1617	26.2837	26.5099	26.256	26.0971	25.9815	25.9101	25.9235
	Q9I6D8	pfpI	7	71.5	19.245	92.335	2.09E+09	51	179	26.1099	26.0321	26.152	26.1302	25.9553	25.7263	26.358	25.692
	Q9I6D9	PA0354	8	31.7	43.77	49.194	1.38E+09	57	398	25.0012	25.2596	25.4678	24.8688	25.0131	25.1018	25.2643	25.2874
xy-acid deh	Q9I6E0	ilvD	28	58.8	65.159	281.77	1.54E+10	184	612	27.7002	27.9851	27.8157	27.7378	27.9402	27.8492	27.8114	27.747
ofolate red	Q9I6E3	folA	6	58.3	18.203	82.231	2.59E+09	51	168	27.0307	27.1331	26.7347	27.2111	26.64	27.0199	26.9324	26.8987
	Q9I6E9	PA0344	6	19.4	49.98	17.555	2.54E+08	17	459	23.9922	23.514	24.636	24.0655	24.231	24.2004	24.4313	23.4072
idylate syn	Q9I6F1	thyA	13	60.2	30.016	57.482	3.06E+09	88	264	26.3996	26.4382	26.0281	26.6428	26.7551	26.5802	26.4284	26.3236
	Q9I6F6	PA0335	13	79.7	24.385	141.39	5.54E+09	110	217	26.7323	26.8449	26.7199	26.811	26.7262	26.6407	26.5016	26.4479
onine dehyc	Q9I6G0	ilvA1	21	60.9	55.351	165.73	6.35E+09	147	504	26.8787	26.99	26.8616	26.4254	26.7129	27.0147	26.7762	26.925
hosphate is	Q9I6G1	rpiA	14	79.8	23.721	212.6	1.73E+10	141	223	28.8864	28.9841	28.5487	28.9607	28.7127	28.8176	28.6877	28.7334
39 protein I	Q9I6G2	PA0329	8	60.9	11.773	132.14	1.45E+10	93	110	28.8145	29.0337	29.7736	28.6768	29.2052	28.9902	29.4787	28.6143
	Q9I6H3	PA0318	8	61.1	23.843	104.87	1.34E+10	94	221	28.9859	28.5916	27.9727	28.8167	28.1204	28.4809	28.2408	28.4941
	Q9I6H4	PA0317	19	49.8	51.286	106.35	6.94E+09	132	464	27.1652	27.2844	27.1887	26.857	27.2825	27.5754	27.3388	27.4455
	Q9I6H5	serA	23	73.3	44.216	230.47	2.68E+10	198	409	29.294	29.4493	29.5217	29.034	29.3688	29.5096	29.651	29.6361
	Q9I6H6	PA0315	11	69.7	15.463	103.28	4.01E+10	151	145	29.7778	28.6694	27.228	29.762	30.0594	30.9401	29.7909	30.8558
	Q9I6H7	PA0314	13	50.8	28.005	110.09	1.12E+10	119	256	27.5682	27.7104	27.3741	27.5461	27.3842	27.5302	27.514	27.3594
	Q9I6I2	PA0309	16	64.4	27.021	182.57	3.06E+09	75	250	26.9135	25.5653	24.9832	26.84	26.1377	26.8597	26.6944	26.6923
-transporti	Q9I6I9	spuF	10	39.8	42.783	73.106	4.11E+09	63	384	27.1077	27.3828	27.4944	26.4286	26.9417	27.1252	27.2839	27.2076
ng periplas	Q9I6J1	spuD	19	57.2	40.629	229.48	2.97E+10	161	367	29.4773	30.186	29.8588	29.0403	29.1271	29.3297	29.9163	29.3555
	Q9I6J2	spuC	17	59.4	50.452	174.07	1.66E+10	152	456	29.4112	29.4071	29.5807	29.0758	28.9482	28.8542	28.9956	28.9567
	Q9I6J3	spuB	17	41.2	50.965	114.94	4.15E+09	114	452	26.9048	27.0621	27.1252	26.5676	26.1224	26.2335	26.4303	26.6773
	Q9I6J4	spuA	8	58	26.477	128.28	1.72E+09	47	250	26.7939	24.4275	23.6417	26.8602	24.2184	25.525	22.6051	25.0272
	Q9I6J5	spuI	13	52.2	50.907	154.21	3.14E+09	80	458	26.2902	26.3602	26.0113	26.1514	26.5034	26.4668	26.4055	26.882
nport ATP-	Q9I6L0	cysA	11	62.3	36.795	184.41	4.01E+09	75	329	26.7803	26.8655	27.0978	26.3654	26.6244	26.5933	26.9929	26.9084
te aminotra	Q9I6M4	davT	24	83.1	45.22	301.57	1.2E+11	323	426	31.1566	31.1268	31.273	30.9816	31.3405	31.4049	31.1713	31.1523
lehyde deh	Q9I6M5	davD	28	76.2	51.622	323.31	5.68E+10	319	483	29.8347	30.0217	30.1799	29.7656	30.1441	30.2565	30.5184	30.4756
	Q9I6M8	PA0261	4	39.4	18.391	50.024	5.67E+08	19	165	25.3617	25.4726	25.1773	25.4386	25.0119	24.8348	24.8208	24.9219
	Q9I6N9	PA0250	7	79.2	15.667	127.93	1.88E+09	73	144	26.0147	25.452	26.2339	25.9223	25.7899	25.5179	25.7482	25.4457
	Q9I6Q2	PA0236	7	44.4	27.978	45.807	9.7E+08	38	259	25.2865	25.1655	25.4845	25.5676	25.4845	25.4014	25.4863	25.4748
	Q9I6Q7	pcaD	6	34.6	28.017	41.083	6.24E+08	24	263	25.2846	25.259	24.7098	25.2628	25.1485	25.0821	24.7749	25.0077
d phosphat	Q9I6U4	PA0190	6	45.2	26.067	26.098	7.28E+08	27	241	25.065	24.5385	22.6748	25.171	25.3315	25.8972	25.2941	25.9855
	Q9I6W2	PA0170	3	46	14.553	23.567	2.46E+08	14	126	23.8529	22.719	23.5389	24.4684	22.8201	24.7296	24.3957	24.19
	Q9I6W8	PA0164	7	19.3	58.453	19.118	2.55E+08	17	538	24.2126	23.4321	22.6684	24.082	22.8973	23.7696	23.4635	24.0464
	Q9I6X3	PA0159	10	59	34.715	87.498	6.68E+08	43	312	24.7756	24.6317	24.8743	24.5981	24.4991	24.9767	24.8825	24.93
	Q9I6X7	pcaR	5	33.3	30.581	28.999	6.37E+08	30	279	24.9009	24.7288	25.3923	25.156	25.2489	25.5762	25.4573	25.3913
nine deami	Q9I6Y4	PA0148	13	58.2	36.163	137.99	5.6E+09	89	316	27.413	27.3649	27.3669	27.6875	27.5889	27.2565	27.3924	27.1883
	Q9I6Y9	nuh	7	36.6	37.542	23.096	5.39E+08	28	350	22.6517	24.9571	25.2366	25.3035	23.9215	24.9028	25.0545	24.9185
	Q9I6Z3	ahpC	15	88.2	20.541	120.16	2.22E+11	189	187	32.9254	33.0532	32.931	32.6678	32.8812	32.7794	32.92	32.7491
yruvate an	Q9I700	bauA	21	60.3	48.382	242.18	1.11E+10	148	448	27.194	27.1834	26.6298	26.7908	27.3175	27.5536	27.0525	28.0776
ropanoate	Q9I702	bauC	23	71.4	53.322	323.31	3.8E+10	252	497	29.3624	29.4087	29.611	28.901	28.3161	28.9313	28.6711	29.3016
	Q9I704	PA0128	5	37.2	12.164	58.955	7.2E+09	55	113	28.58	28.7228	28.2634	28.7027	28.2834	28.4561	28.7004	28.5444
	Q9I707	PA0125	3	44	8.5685	16.375	5.65E+08	24	75	25.9567	24.518	23.7552	25.9358	24.3395	26.1807	24.8285	25.5431
	Q9I708	PA0124	2	40.9	10.462	53.13	8.9E+08	16	93	26.5191	26.3682	26.306	26.3312	26.5206	26.5956	26.5427	26.7851
	Q9I712	PA0120	4	32	25.463	22.409	2.37E+08	17	228	24.4342	22.3735	24.2933	24.6416	23.1267	23.052	23.8236	23.7449
	Q9I714	PA0118	3	23.1	21.473	6.691	1.24E+08	19	195	23.5512	23.3803	23.0529	23.8521	22.5087	23.3049	23.9389	23.3309
	Q9I716	PA0116	3	27.4	18.212	12.204	1.27E+08	16	157	23.8575	23.5395	24.0754	24.1305	22.9595	24.2599	23.6839	24.08
onic anhyc	Q9I730	PA0102	12	61.6	26.622	158.88	1.33E+10	93	242	28.9063	28.79	28.8667	28.6818	29.1766	29.1242	29.6883	29.0704
	Q9I740	PA0092	4	42.6	10.678	10.274	5.34E+08	23	94	24.9418	24.4221	23.0145	25.0572	24.2312	24.986	24.3553	24.8284
	Q9I761	PA0071	7	22.8	61.923	47.86	4.31E+08	39	570	23.6728	23.7768	24.0229	23.7907	23.8825	23.7987	24.3063	23.9788
	Q9I765	prlC	31	60.4	76.043	307.03	1.28E+10	216	681	27.6853	27.8321	27.6	27.9369	27.9469	27.8034	27.6294	27.5734
-nucleotida	Q9I767	PA0065	7	48.9	24.951	30.584	2.8E+08	18	221	24.0394	24.2953	22.704	23.2869	24.1057	24.3264	23.9486	23.9571

	Q9I777	PA0055	5	44.6	18.433	56.346	1.84E+09	38	168	25.9139	26.2003	26.4769	26.4108	26.0687	25.5993	25.8459	25.5812
	Q9I793	PA0039	5	52	7.9233	15.099	1.18E+09	21	75	27.1886	24.7741	22.441	27.0389	23.2179	26.6051	23.8016	26.0596
rbamoyl-AN	Q9I7A5	tsaC	6	58.9	20.371	75	8.24E+08	30	185	25.1804	25.1025	25.2122	25.1521	24.8303	25.319	25.1165	24.7405
	Q9I7A7	PA0020	12	52.2	37.586	151.99	5.94E+09	69	341	27.6214	27.6424	27.8749	27.4849	26.9802	26.863	27.0621	27.3852
ide deform	Q9I7A8	def	10	86.9	19.365	135.45	9.46E+09	121	168	27.9725	28.0161	27.7762	28.0861	27.5284	27.5928	27.6581	27.4052
	Q9I7B0	trkA	13	45.7	49.926	64.181	1.45E+09	56	457	25.6637	25.6492	25.9374	25.0927	25.4329	25.5473	25.7747	25.7818
NA ligase al	Q9I7B7	glyQ	9	47.3	36.14	104.53	8.94E+09	81	315	28.6488	28.4745	28.0671	28.8089	29.009	28.7784	28.2638	28.178
NA ligase b	Q9I7B8	glyS	31	50	73.973	314.82	9.54E+09	206	684	26.9537	26.9435	27.0282	26.7406	26.891	27.0431	27.0938	27.3102
ptose-1,7-b	Q9I7C0	gmhB	6	54.5	19.115	58.057	3.74E+09	52	178	28.1234	28.2345	27.4334	28.337	28.122	28.3798	27.8591	28.285
gyrase subu	Q9I7C2	gyrB	36	61.9	90.188	255.39	8.55E+09	197	806	27.1171	27.4305	26.8451	27.2076	27.6822	27.5334	27.2535	28.087
nerase III su	Q9I7C4	dnaN	17	54	40.694	323.01	4.15E+10	188	367	29.9261	30.0943	30.3271	29.8024	29.8841	29.9323	30.1214	29.861
ication init	Q9I7C5	dnaA	21	59.1	57.754	152.48	3.33E+09	100	514	25.9654	26.1182	26.054	25.9588	26.1603	26.186	26.1044	26.3383
5-phosphat	Q9KGU6	dxr	15	54.8	42.508	149.97	2.17E+09	68	396	26.1922	26.0069	26.0723	26.0411	26.4133	26.4887	26.4005	26.3649
ose-5-phos	Q9KGU7	dxs	17	33.3	68.049	88.225	1.64E+09	72	627	25.7329	25.8707	25.5501	25.5206	25.871	26.1685	25.936	26.3196
nslocase su	Q9LCT3	secA	41	57	103.85	323.31	2.28E+10	356	916	28.3758	28.5279	28.5871	28.41	28.8102	28.8357	28.8671	29.0657
e--D-alanin	Q9LCT6	ddlB	14	61.4	34.363	109.23	5.04E+09	104	319	26.9503	27.0276	27.0785	27.016	26.988	27.2589	27.3297	27.2903
ycolate pho	Q9S586	gph1	5	32.4	29.812	12.379	3.74E+08	17	272	24.3106	23.9755	24.1717	24.176	24.7531	24.8347	24.3834	24.8992
nogluconol	Q9X2N2	pgl	9	48.3	25.571	80.76	1.25E+09	44	238	25.3603	25.1244	24.9394	25.2435	25.3502	25.4189	25.5306	25.32
Thioredoxin	Q9X2T1	trxA	8	74.1	11.87	82.25	1.03E+11	174	108	32.5341	31.5223	30.8505	32.7362	31.5396	32.3863	31.5319	32.1547
P-N-acetylg	Q9X6P4	lpxA	10	52.3	28.009	52.269	3.51E+09	60	258	27.1533	27.2629	27.2575	26.9702	27.3176	27.3113	27.2687	27.0865
ninopropyl	Q9X6R0	speE1	16	64.7	32.238	106.89	6.78E+09	101	286	27.4346	27.7155	27.535	27.2248	27.5844	27.6482	27.7621	28.0246
50 protein I	Q9X6V8	PA3998	6	82.8	10.338	101.17	5.19E+09	56	93	27.7737	27.2087	27.1685	27.6667	27.6378	27.4569	27.5957	27.6542
noyltransfe	Q9X6V9	lipB	8	73.3	23.856	33.215	9.58E+08	34	217	25.3359	25.0525	25.4787	23.1592	25.5247	25.2813	25.7854	25.7687
nate--tRNA	Q9XCL6	gltX	27	70.6	56.749	197.49	1.29E+10	218	494	27.5661	27.7968	27.2295	28.0505	28.2287	28.0898	27.8949	28.1813
GTPase Era	Q9XCX8	era	17	64.3	34.549	95.359	7.8E+09	92	305	28.1332	28.2931	28.3282	28.1819	27.8055	28.0692	27.9389	27.9833
bonuclease	Q9XCX9	rnc	10	43.7	25.506	39.538	2.42E+09	60	229	26.1629	26.1391	26.4527	26.2062	26.2334	26.135	26.1119	25.8564
protein] red	Q9ZFE4	fabI	12	63	28.006	71.88	2.03E+09	56	265	25.5828	25.8858	25.9854	25.4195	26.2178	26.0101	25.9326	25.454
xyphospho	Q9ZFK4	kdsA	19	75.1	31.143	97.321	1.45E+10	151	281	28.1427	28.2784	28.3899	28.0465	28.1441	28.3256	28.1825	28.1627
